# Implications and risk of new *versus* persisting intraductal papillary mucinous neoplasms after pancreatic surgery: meta-analysis

**DOI:** 10.1093/bjsopen/zrag087

**Published:** 2026-07-13

**Authors:** Anouk J van Bodegraven, Katharina M Marstaller-Walz, Virginia Padoan, Danhui Heo, Giampaolo Perri, George L Burchell, Martin Loos, Christoph W Michalski, Marc G Besselink, Umberto Cillo, Ingmar F Rompen, Giovanni Marchegiani

**Affiliations:** Department of Surgery, Amsterdam UMC, University of Amsterdam, Amsterdam, the Netherlands; Department of Surgery, Cancer Center Amsterdam, Amsterdam, the Netherlands; Department of General, Visceral, and Transplantation Surgery, Heidelberg University Hospital, Heidelberg, Germany; Department of Hepato-Bilio-Pancreatic Surgery and Liver Transplant Surgery, Department of Surgery, Oncology and Gastroenterology (DiSCOG), Padova University, Padova, Italy; Doctoral School of Experimental and Preventive Medicine, Albert Szent-Györgyi Medical School, University of Szeged, Szeged, Hungary; Department of Hepato-Bilio-Pancreatic Surgery and Liver Transplant Surgery, Department of Surgery, Oncology and Gastroenterology (DiSCOG), Padova University, Padova, Italy; Department of Surgery, Amsterdam UMC, University of Amsterdam, Amsterdam, the Netherlands; Department of General, Visceral, and Transplantation Surgery, Heidelberg University Hospital, Heidelberg, Germany; Department of General, Visceral, and Transplantation Surgery, Heidelberg University Hospital, Heidelberg, Germany; Department of Surgery, Amsterdam UMC, University of Amsterdam, Amsterdam, the Netherlands; Department of Surgery, Cancer Center Amsterdam, Amsterdam, the Netherlands; Department of Hepato-Bilio-Pancreatic Surgery and Liver Transplant Surgery, Department of Surgery, Oncology and Gastroenterology (DiSCOG), Padova University, Padova, Italy; Department of Surgery, Amsterdam UMC, University of Amsterdam, Amsterdam, the Netherlands; Department of Surgery, Cancer Center Amsterdam, Amsterdam, the Netherlands; Department of General, Visceral, and Transplantation Surgery, Heidelberg University Hospital, Heidelberg, Germany; Department of Hepato-Bilio-Pancreatic Surgery and Liver Transplant Surgery, Department of Surgery, Oncology and Gastroenterology (DiSCOG), Padova University, Padova, Italy

**Keywords:** IPMN, pancreatic cystic lesions, recurrence patterns, postoperative recurrence, risk factors

## Abstract

**Background:**

Interpretation of recurrence following resection of intraductal papillary mucinous neoplasms (IPMNs) is hindered by inconsistent terminology and outcome reporting. A distinction between metachronous/*de novo* IPMN development, progression of residual disease, and true recurrence of invasive entities is rarely considered.

**Methods:**

A systematic review and meta-analysis were conducted following PRISMA and Cochrane guidelines (PROSPERO-ID: 1009399). The primary endpoint was postoperative recurrence of non-invasive IPMN and IPMN-derived pancreatic cancer (PC). Recurrence following resection of non-invasive IPMN was reclassified as progression of persistent cysts or *de novo* metachronous IPMN. Secondary endpoints comprised risk-factors for recurrence.

**Results:**

Sixty-six articles with 11 464 patients were included. After a median follow-up of 26 (interquartile range (i.q.r.) 18.0–53.0) to 72 (i.q.r. 5–318) months, the recurrence rate of IPMN-derived PC was 41.9% (1646/3925 patients), with a 5-year pooled recurrence-free survival of 46.6%. Systemic recurrence was most common (1034/1646; 62.8%), followed by locoregional (430/1646; 26.1%). Secondary treatments were administered in 655/1646 patients (39.8%) presenting with recurrence and included chemotherapy (65.5%), surgery (22.1%) and radiation (5.8%). Lymph node involvement (hazard ratio 2.87, 95% confidence interval 1.51 to 5.43) and tubular subtype (hazard ratio 1.68, 1.16 to 2.43) were identified as independent predictors of recurrence-free survival. The overall recurrence rate following pancreatic resection of non-invasive IPMN was 11.2% (831/7446), after median follow-up of 28 (i.q.r. 1–153) to 114 (i.q.r. 12–204) months. Among these, 408 (49.1%) developed as *de novo* lesions in the remnant pancreas and 174 (20.9%) as progression of pre-existing cyst detected at the time of index surgery; the remaining 249 patients (30.0%) could not be reclassified. Non-invasive recurrence was more common (308; 37.1%) than IPMN-derived PC (118; 14.2%), whereas the type of recurrence was unspecified in 405 patients (48.7%). Secondary treatment data were available for 265 patients, of whom 131 (49.4%) underwent reoperation.

**Conclusion:**

The recurrence rate of IPMN-derived PC is high and warrants close surveillance policies. The majority of ‘recurring’ non-invasive IPMNs are *de novo* lesions. Standardized reporting, distinguishing true recurrence from *de novo* development and progression of residual disease, is essential to stratify recurrence risk and optimize surveillance protocols accurately.

## Teaser

Recurrence rate of intraductal papillary mucinous neoplasms (IPMN)-derived pancreatic cancer is high and warrants close surveillance policies. The majority of ‘recurring’ non-invasive IPMNs are *de novo* , metachronous lesions. Standardized reporting, distinguishing true recurrence from *de novo* development and progression of residual disease is essential to stratify recurrence risk and optimize surveillance protocols accurately.

## Introduction

Intraductal papillary mucinous neoplasms (IPMNs) of the pancreas have the potential to progress from low- to high-grade dysplasia, and ultimately to invasive carcinoma (IC)^1^. Therefore, they require careful management, including radical surgical resection with the aim to remove high-grade dysplasia (HGD) and IC^2^. Despite this, many IPMNs are resected while still presenting low-grade dysplasia (LGD), reflecting the persistent challenges of accurate preoperative assessment and limited specificity in the available guidelines^1,3^. Although the goal of surgery is the complete removal of suspicious lesions, recurrence has been described for non-invasive IPMN even after margin-negative resection^4–6^. Once cysts have progressed into IPMN-derived pancreatic cancer (PC), the likelihood of disease recurrence is significantly higher, with reported rates ranging from 41% to 65%^3,7–11^. Patterns and frequency of recurrence are known to be largely dependent on epithelial subtypes, surgical margin status and lymph node involvement^12,13^.

As IPMNs are primarily managed through partial pancreatectomies, routine assessment of recurrence in the pancreatic remnant is essential. Due to the lack of standardized terminology in current literature and the unanimous use of ‘recurrence’ and ‘progression’ without explicit distinction, it is often unclear whether lesions have newly developed or were already present at index surgery. From a biological standpoint, only IPMN-derived PC has the potential to recur^4^. This conceptual unclarity reflects the complex biology of IPMN, often presenting with multifocal lesions^4,14^. The underlying cause is hypothesized to be a pancreatic ‘field defect’, resulting in predisposition for metachronous *de novo* IPMN development in the remnant pancreas^4,14–16^. Consequently, the terms ‘recurrence’ and ‘progression’ are often applied indiscriminately across biologically distinct scenarios, not adequately reflecting their different clinical implications. Once a resection for a non-invasive entity is performed, it is important to distinguish the occurrence of a new IPMN during follow-up from the persistence or progression of residual cysts already present at the time of index surgery^4,6^.

To manage the risk of recurrent disease, current guidelines recommend regular clinical visits and radiological imaging over a period of 5–10 years, with possible extension to lifelong surveillance in surgically fit patients. Significant differences in long-term outcome according to the degree of dysplasia are however not yet reflected in these recommendations^1,3,17,18^.

This study aims to identify and summarize the existing literature on the actual recurrence rates and patterns after surgical resection of both invasive and non-invasive IPMNs. Reclassifying ‘recurrence’ as either the progression of a persistent cyst or the metachronous development of a *de novo* IPMN would enable a more nuanced understanding of disease behaviour. This approach may ultimately facilitate tailored surveillance, improving long-term patient outcomes and quality of care.

## Methods

**Fig. 1:**
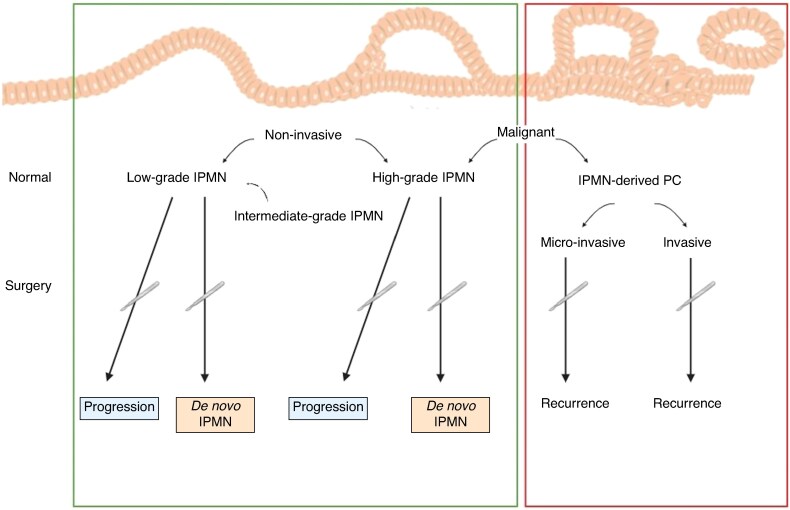
Reclassification system for recurrence following resection of non-invasive IPMN and IPMN-derived PC Progression of a persistent cyst in remnant pancreas, possible after margin+ resection; metachronous/*de novo* IPMN: growth of a new separate cyst not present at time of index surgery, possible after margin resection. IPMN, intraductal papillary mucinous neoplasm; PC, pancreatic cancer.

The objective of this systematic review was to assess the rate of recurrence after resection of IPMN-derived PC and non-invasive IPMN separately (*Fig. 1*). A meta-analysis investigating risk factors for recurrence was conducted in the cohort of patients with IPMN-derived PC. The criteria of the *Cochrane Handbook for Systematic Reviews of Interventions*^19^ were adhered to and the PRISMA guidelines^20^ followed; the PRISMA checklist is provided in *Table S1*. The study protocol was previously registered and published on PROSPERO (ID: 1009399). The search was constructed based on a population, intervention, control, and outcomes format (*Table S2*). The study population comprised patients who underwent pancreatic resection for IPMN. The primary outcome was defined as the rate of recurrence in the form of newly developed cysts or progression of persisting disease in the remnant pancreas after surgical resection. Secondary outcomes comprised possible predictors of recurrence.

### Literature search strategy

A systematic search of PubMed™ (National Library of Medicine, Bethesda, MD, USA), Embase™ (Elsevier, Amsterdam, Netherlands) and the Wiley Online Library™/Cochrane Library™ (John Wiley & Sons Ltd, Hoboken, NJ, USA) was performed. The database search covered the period from inception to 25 March 2025 and was conducted by G.L.B. and A.v.B. The search was conducted in English and included keywords and free text terms for (synonyms of) ‘splenectomy’ combined with (synonyms of) ‘pancreatectomy’ and ‘pancreatic intraductal neoplasms’ (*Table S3*). No limitations on date were applied in the search, and duplicates were removed. Conference abstracts, papers, reviews, notes, errata, chapters, and letters were excluded. To expand the search, additional articles were sought by direct citation searching.

### Study selection

Assessment for inclusion eligibility was independently performed by two authors (A.v.B. and K.M.) with the use of Rayyan™ (Rayyan Systems Inc., Doha, Qatar)^21^. Discrepancies were resolved by consensus of a third reviewer (V.P.). Articles were eligible for inclusion when radiographically suspected or histologically confirmed recurrence, persistence, or growth of new IPMN lesions were reported following resection for either non-invasive IPMN or IPMN-derived PC (*Fig. 1*). Studies reporting on both entities were only included if the primary outcome measurement could be extracted separately. Studies using the term ‘malignancy’ were also included. Data were subsequently reclassified as either non-invasive or IPMN-derived PC according to current guidelines^1,22,23^. Aiming to apply the classification of IPMN recurrence presented in *Fig. 1*, studies lacking data on recurrence, progression, or persistence rates were excluded. Studies were further excluded if patients did not undergo surgical resection as primary treatment or if articles were not written in English. Since the World Health Organization formally adopted the term ‘IPMN’ in 2000, distinguishing these lesions from other cystic pancreatic neoplasms, studies published before 2000 were excluded^24^. Studies with a median follow-up of under 2 years were excluded, as this duration was deemed insufficient to evaluate disease recurrence. Furthermore, non-original research, case reports, and small case-series reporting on fewer than ten patients with non-invasive IPMN or IPMN-derived PC were excluded. When multiple studies were published by the same authors or were based on the same patient collective, only one was included for further analysis to minimize potential bias due to overlapping cohorts.

### IPMN classification and outcomes of interest

IPMN-derived PC was defined as IPMN histologically presenting with IC, including colloid, tubular, and oncocytic type^1,25^. Microinvasive IPMN (superficial, < 5 mm invasive, T1a) was considered a subtype of IPMN-derived PC, but documented separately due to the difference in prognostic value^26,27^. Non-invasive IPMN comprised LGD (including adenoma, moderate dysplasia, and borderline) and HGD (including carcinoma *in situ* and severe dysplasia)^1,23^. Data reported on intermediate-grade dysplasia (including borderline) were recategorized as LGD, in accordance with current guidelines (*Fig. 1*)^1,28^.

The primary outcome of interest was recurrence rate following surgical resection for both non-invasive and IPMN-derived PC. For non-invasive IPMN, ‘recurrence’ after margin-positive resections was considered as disease persistence. Detection of cysts after margin-negative resection or radiological description of secondary cyst development in an unrelated section of the pancreatic gland (for example, in the tail after index surgery for a cyst in the head) was considered metachronous/*de novo* IPMN development (*Fig. 1*). The reclassification was conducted by two independent authors (A.v.B. and V.P.) and discrepancies were resolved by a senior author (G.M.). If not provided in the article, the recurrences were reclassified in the previously mentioned categories, aligning with the original source data. After index surgery for IPMN-derived PC, recurrence was determined by the presence of solid mass, main pancreatic duct (MPD) dilation > 5 mm or cyst growth on imaging as described in the Kyoto guidelines (CQ3-3)^1^, including development of metachronous pancreatic ductal adenocarcinoma (PDAC) in the remnant pancreas.

In case of recurrence, the time to recurrence (months) and location were analysed. Recurrence was assessed via radiologic imaging or confirmed histologically if available. If the diagnostic modality for recurrence was not reported but detailed site information was available, recurrence assessment was considered to have been based on radiologic follow-up. The recurrence location was classified as systemic, locoregional, or not specified. Locoregional recurrence comprised local persistence in margin-positive cases or skip progression to the remnant pancreas in margin-negative situations^29^. Multisite recurrences including distant metastases were described as systemic recurrence. Recurrences reported as ‘extra-pancreatic’ in the original source and not clearly specified as locoregional (that is, regional lymph nodes or mesenteric vessels) were classified as systemic. Histological recurrence type was categorized as non-invasive IPMN, IPMN-derived PC, metachronous PDAC, or otherwise marked as not specified. Secondary outcomes comprised recurrence-free survival (RFS), its potential predictors, and overall survival (OS).

### Recorded variables

Data on patient demographics (age, sex, American Society of Anesthesiologists classification^30^), morphological and surgical characteristics, final histopathology from index surgery, if applicable from secondary surgery or biopsy, and follow-up information were reported.

Morphological characteristics at time of index surgery detailed IPMN location (head/neck, body/tail, or other), type (main-duct (MD), mixed-type (MT), or branch-duct (BD)), cyst size (cm), and presence of multifocality. Additionally, worrisome features (WF) and high-risk stigmata (HRS) at the time of index surgery were extracted, following the Kyoto guidelines^1^. For the subgroup of IPMN-derived PC, tumour size was also recorded^3^.

Surgical characteristics included type of resection (pancreatoduodenectomy (PD), left pancreatectomy (LP), total pancreatectomy (TP), parenchyma-sparing resection (for example, enucleations, other)), and surgical approach (open or minimally invasive surgery). In cases of index surgery for IPMN-derived PC, the use of adjuvant therapy was documented and specified.

Regarding histopathology, the highest grade of dysplasia, cyst size, and epithelial subtype for non-invasive IPMN (pancreatobiliary, gastric, intestinal, or oncocytic) were documented^3^. For IPMN-derived PC, histological subtype (colloid, tubular, oncocytic, other) and tumour node metastasis stage, including number of positive lymph nodes, margin status, lymphovascular invasion (LVI), and perineural invasion (PNI), were reported^25^. Margin status was considered positive when lower or higher degrees of dysplasia were found.

Follow-up information included median follow-up time (months), sample size, secondary therapy after recurrence (surgical re-resection, chemotherapy, palliative care, other), DFS, and OS.

A detailed overview of the recorded variables is provided in *Table S4*.

### Risk of bias assessment

The risk of bias was assessed with the Risk of Bias in Non-randomized Studies—of Exposure (ROBINS-E) tool^31^, and subsequently summarized and presented visually.

### Statistical analysis

Statistical significance was set at two-sided *P* < 0.05 for all analysis. Summary estimates are presented with 95% confidence intervals (c.i.). Between-study heterogeneity was assessed using the *I*^2^ statistic, with *I*^2^ values of 25%, 50%, and 75% representing low, moderate, and high heterogeneity, respectively^32^. When confidence intervals were not reported, 95% c.i. were estimated using binomial approximations based on sample size. Proportions were pooled using logit transformation and inverse-variance weighting to stabilize variance and account for between-study heterogeneity, with confidence intervals for pooled estimates derived using the Q-profile method.

The primary outcome was postoperative recurrence. Crude recurrence proportions were synthesized using a single-arm meta-analytic approach. For each included study, the recurrence rate was derived by dividing the number of patients who developed postoperative recurrence by the total number of patients receiving follow-up. These proportions were pooled with an inverse-variance weighted random-effects model using logit transformation, and between-study variance was estimated with the DerSimonian and Laird method.

Recurrence was further assessed as a time-to-event outcome using RFS. In patients with IPMN-derived PC, a single-arm meta-analysis was performed to estimate pooled RFS rates at predefined postoperative time points (1, 3, 5, and 10 years). RFS estimates were extracted directly from published survival data or reconstructed from Kaplan–Meier curves when necessary. When studies reported cumulative recurrence rates instead of RFS, RFS was calculated as the complement (1–cumulative recurrence rate). DFS and progression-free survival metrics were considered equivalent to RFS when clearly referring to the absence of recurrence. The number of recurrence-free patients at each time point was estimated by applying the reported proportion to the study population. Censoring and loss to follow-up were addressed by extracting hazard ratios (HRs) from multivariable Cox proportional hazards models reported in the original studies.

To explore heterogeneity and assess the robustness of pooled estimates, sensitivity and subgroup analyses were performed for the 5- and 10-year RFS outcomes. For the sensitivity analyses, a leave-one-out method and an analysis including only studies with a low–moderate risk of bias was conducted to evaluate the influence of individual studies on pooled estimates and *I*^2^ values. Additionally, subgroup analyses were carried out for the 5- and 10-year RFS according to publication year (before 2020 *versus* 2020 and after) and centre type (single-centre *versus* multicentre). These stratifications were selected to account for potential differences in surgical standardization and data consistency over time and across institutional settings.

Analyses of factors associated with recurrence were conducted using HRs derived from Cox proportional hazards models. HRs for recurrence were obtained from multivariable Cox regression models reported in the included studies. Although outcomes defined as recurrence, RFS, or cumulative risk of distant recurrence present distinct clinical and statistical entities, they were treated as equivalent for the purposes of analysis due to their related clinical contexts and overlapping implications. In studies^32^ that reported HRs without corresponding 95% c.i., standard errors and confidence intervals were estimated using the log (HR) divided by the *Z* value derived from the reported *P* value.

All analyses were conducted using R^®^ (version 3.6.1; R Foundation for Statistical Computing, Vienna, Austria) and Review Manager 5.4™ (The Cochrane Collaboration, Copenhagen, Denmark). A large language model (OpenAI^®^ GPT-5™ (OpenAI, San Francisco, CA, USA), accessed August 2025) was used to assist for language refinement when drafting portions of the manuscript. All content generated by the model was reviewed and verified by the authors. Artificial intelligence was not used for data collection, analysis, or interpretation of study findings.

## Results

### Literature search and risk of bias assessment

**Fig. 2:**
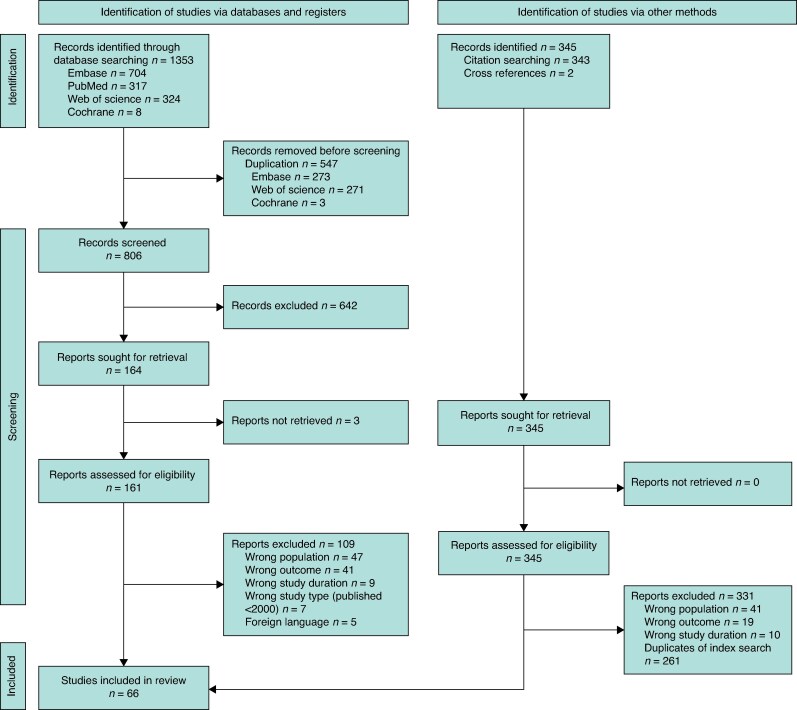
PRISMA flow chart

A total of 1698 records were initially identified (*Fig. 2*). Sixty-six articles met the inclusion criteria, all of which were retrospective single- or multicentre studies. Of these, 27 studies reported solely on the recurrence rate following surgical resection of non-invasive IPMN, whereas 10 studies provided data on IPMN-derived PC only. The remaining 29 studies reported on both disease entities. Forty-three studies included patients who underwent index surgery before the year 2000 (*Table S5*).

Risk of bias assessment was scored low for confounding in 22 studies, some concerns in 35, and serious risk in 9. Twenty-two studies did not deliver enough information to assess the risk of bias in all domains adequately. Of these, 12 were scored with a high risk of bias in at least one domain. Seventeen studies had a high risk of bias in one of the domains, whereas 28 studies only had some concerns of bias (*Fig. S1*).

### Study population

Overall, 66 articles reporting on 11 464 patients undergoing index surgery for either non-invasive IPMN or IPMN-derived PC were included. Within this population, 7514 patients (65.5%) underwent surgical resection for non-invasive lesions and 3950 patients (34.5%) for IPMN-derived PC. Within the non-invasive subgroup, LGD was found in 4907 of 7514 (65.3%) and HGD in 1796 (23.9%) patients. The grade of dysplasia grade was not specified in 811 (10.8%) resected non-invasive IPMN (*Tables S5, S6*).

#### IPMN-derived PC

##### Patients’ characteristics

A total of 39 studies reported data on 3950 patients undergoing surgical resection for IPMN-derived PC. Overall, sex was specified for 1538 (38.9%) patients, comprising 56.7% men and 43.3% women. The median age ranged from 46.0 (interquartile range (i.q.r.) 44.0–48.5) years^33^ to 72 (i.q.r. 64–77) years^9^ (*Table S8*).

##### IPMN characteristics at time of index surgery

In the resected specimen, IPMN-colloid carcinoma was found in 772 of 3950 (19.5%), oncocytic in 61 (1.5%), and tubular carcinoma in 1573 (39.8%) patients. In 1544 patients (39.1%) the histological type was not specified (*Table S8*).

Eight articles specified cyst location for a total of 832 patients (21.1%). IPMNs located in the head/neck were most prevalent (519 of 832; 62.4%), followed by lesions in the body/tail (230; 27.6%). In 83 patients (10.0%), cysts were located diffusely (62 of 832; 7.5%) or within the whole gland (21 of 832; 2.5%). Multifocality was reported in seven articles (413 of 3950; 10.5%), out of which 69 patients (16.7%) presented with multifocal cysts before surgery.

Most IPMNs (1109 of 3950; 28.1%) were MT, and cyst size ranged from a mean(standard deviation (s.d.)) of 1.6(1.0) cm^34^ up to 4.9(0.6) cm^35^, and the mean(s.d.) tumour size from 2.9(0.3) cm^35^ to 4.9(3.8) cm^34^ (*Table S8*).

##### Surgical and pathological characteristics

The type of surgical resection was specified in 16 articles, for a total of 2633 of 3950 patients (66.7%). Of these, PD was performed in 1529 (58.1%), LP in 604 (22.9%), TP in 455 (17.3%) and partial pancreatectomy in 45 (1.7%) patients. For the remaining 1317 patients (33.3%), the operative procedure was not specified (*Table S9*). In terms of oncologic treatment, the use of adjuvant therapy was specified in 15 studies (2650 of 3950; 67.1%). Adjuvant therapy was recommended or started for 1749 of 2650 patients (66%), without specifying the completion for 19 patients (0.7%) (*Table S9*).

Details on the resection margin status were documented in 1675 of 3950 patients (42.4%), with positive margins in 356 (21.3%) of these and negative in 1319 (78.7%) (*Table S9*). Lymph node status was specified in 28 articles, reporting on 3491 of 3950 patients (88.4%). Of these, positive lymph nodes were found in 1204 patients (34.5%), whereas 2287 patients (65.5%) were node-negative (*Table S9*). Information on LVI was reported in 12 articles (2274 of 3950; 57.6%), and LVI was present in 910 of 2274 patients (40.0%). PNI was reported in 14 articles (2377 of 3950; 60.2%) and found positive in 1018 of 2377 patients (42.8%) (*Table S9*).

##### Recurrence

**Fig. 3:**
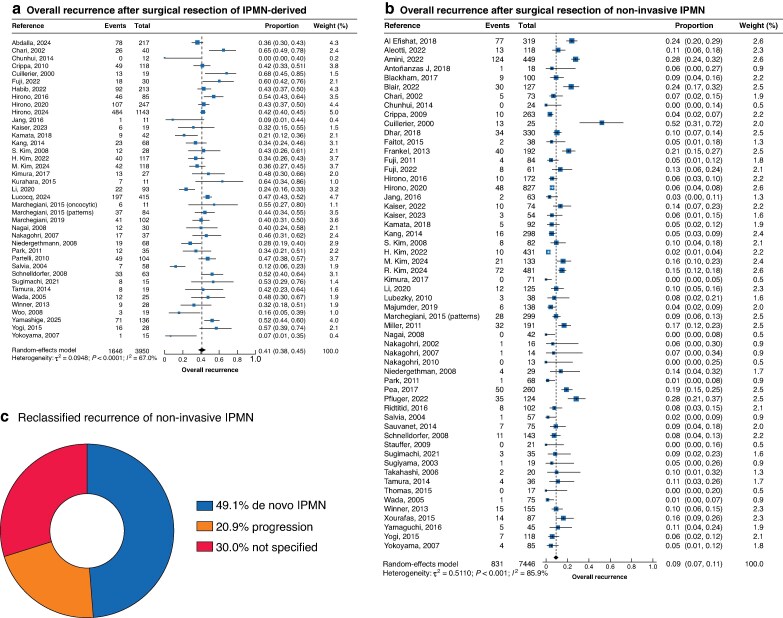
Polarized visualization of overall recurrence following surgical resection of IPMN-derived PC and non-invasive IPMN, and distribution of reclassified recurrence types of resected non-invasive IPMN **a** IPMN-derived PC (1646), **b** non-invasive IPMN (831), **c** distribution of reclassified recurrence types of resected non-invasive IPMN (831). Error bars represent 95% confidence intervals. IPMN, intraductal papillary mucinous neoplasm; PC, pancreatic cancer.

As seven studies failed to report on follow-up data for a subset of patients (25 of 3950; 0.6%), follow-up information after resection of IPMN-derived PC was available for 3925 patients (99.4%). The overall recurrence rate following surgery for IPMN-derived PC was 41.9% (1646 of 3925), with a median follow-up of 26 (i.q.r. 18.0–53.0) months^33^ to 72 (i.q.r. 5–318) months^36^ (*Table 1*, *Fig. 3*, *Table S10*). In 3039 (77.4%) patients, the recurrence was confirmed histologically, whereas the diagnosis for the remaining 886 (22.6%) was solely based on radiological imaging (*Table S11*).

**Table 1:** Recurrence patterns and characteristics following surgical resection of non-invasive and invasive IPMN

Outcomes	Number of patients/Follow-up time [months]
**Resected non-invasive IPMN**	7514
Total follow-up sample size	7446 (99.1%)
Follow-up time (months), median (i.q.r.)	4.7 (0.1–10.6)–114 (12–204)
Overall recurrence	831 (11.2%)
*De novo* /metachronous IPMN	408 (49.1%)
*De novo*/metachronous IPMN, % of follow-up population	5.5%
Progression persistent cyst	174 (20.9%)
Progression persistent cyst, % of follow-up population	2.3%
Not specified recurrence	249 (30.0%)
Not specified recurrence, % of follow-up population	3.3%
Type	
Non-invasive	308 (37.1%)
IPMN-derived PC	118 (14.2%)
Not specified	405 (48.7%)
Location	
Systemic	71 (8.5%)
Locoregional	368 (44.3%)
Not specified	392 (47.2%)
**Resected IPMN-derived PC**	3950
Total follow up sample size	3925 (99.4%)
Follow-up time (months), median (i.q.r.)	26 (18.0–53.0)–72 (5–318)
Overall recurrence	1646 (41.9%)
Type	
Non-invasive	35 (2.1%)
IPMN-derived PC	469 (28.5%)
Metachronous PDAC	36 (2.2%)
Not specified	1106 (67.2%)
Location	
Systemic	1034 (62.8%)
Locoregional	430 (26.1%)
Not specified	182 (11.1%)

Values are *n* (%) unless otherwise stated. IPMN, intraductal papillary mucinous neoplasm; i.q.r., interquartile range; PC, pancreatic cancer; PDAC, pancreatic ductal adenocarcinoma.

**Fig. 4:**
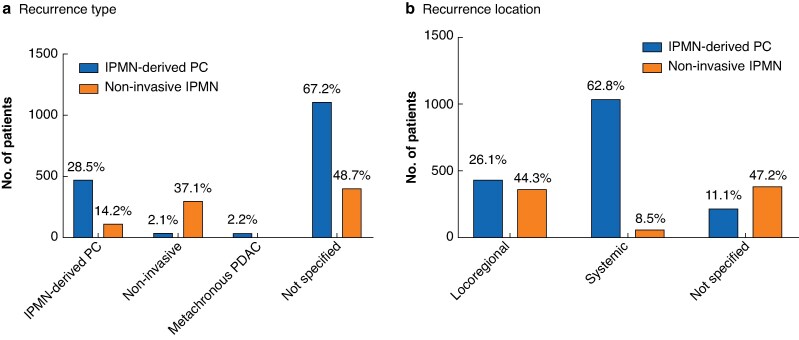
Recurrence types and location following surgical resection of IPMN-derived PC and non-invasive IPMN **a** Recurrence type**, b** recurrence location. IPMN, intraductal papillary mucinous neoplasm; PC, pancreatic cancer; PDAC, pancreatic ductal adenocarcinoma.

In terms of recurrence types, 35 of 1646 (2.1%) were non-invasive and 469 (28.5%) were IPMN-derived PC. Additionally, 36 patients (2.2%) presented with metachronous PDAC, whereas the histologic type of recurrence was not specified for the remaining 1106 patients (67.2%) (*Table 1*, *Fig. 4a*, *Table S10*). Systemic metastasis was the most common recurrence type (1034; 62.8%), followed by locoregional recurrence (430; 26.1%). In 182 patients (11.1%) the recurrence location was not specified (*Table 1*, *Fig. 4b*, *Table S10*).

Secondary treatment was performed in 655 of 1646 patients (39.8%) presenting with disease recurrence. Of these, most patients underwent adjuvant chemotherapy (429; 65.5%), followed by secondary surgery (145; 22.1%), and radiotherapy (38; 5.8%). Forty-three patients (6.6%) received either palliative care or not further specified conservative treatment (*Table S10*). The 5-year OS ranged from 31%^35^ to 91.3%^37^ and the 5-year DFS from 45.4%^34^ to 71.4%^37^ (*Table S10*).

**Fig. 5:**
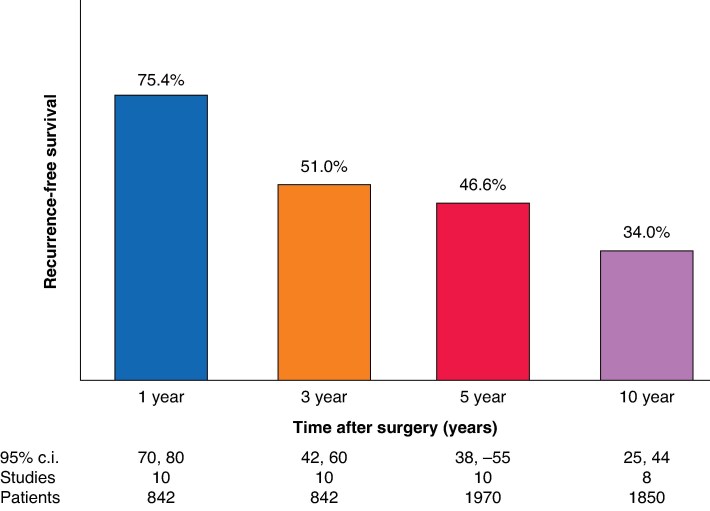
Summary of 1-, 3-, 5-, and 10-year pooled recurrence-free-survival analysis for IPMN-derived PC c.i., confidence interval; IPMN, intraductal papillary mucinous neoplasm; PC, pancreatic cancer.

Pooled RFS estimates for IPMN-derived PC demonstrated a progressive decline over time. At 1 year, the pooled RFS was 75.4% (95% c.i. 70.0 to 80.0) across ten studies (842 of 3950 patients; 21.3%) with moderate heterogeneity (*I*^2^ = 61.9%, *P* = 0.005) (*Fig. 5*, *Fig. S2a*). After 3 years, the pooled RFS declined to 51.0% (95% c.i. 42.0 to 60.0) across the same ten studies (842), with high heterogeneity (*I*^2^ = 78.2%, *P* < 0.0001) (*Fig. 5*, *Fig. S2b*). At 5 years, the pooled RFS was 46.6% (95% c.i. 38.0 to 55.0) across ten studies (1970 of 3950; 49.9%), with high heterogeneity (*I*^2^ = 89.2%, *P* < 0.0001) (*Fig. 5*, *Fig. S2c*). Finally, a 10-year RFS of 34.0% (95% c.i. 25.0 to 44.0) was found across eight studies (1850 of 3950; 46.8%), with high heterogeneity (*I*^2^ = 93.5%, *P* < 0.0001) (*Fig. 5*, *Fig. S2d*). The leave-one-out analysis demonstrated consistent pooled 5- and 10-year RFS estimates, identifying the study conducted by Hirono *et al*.^7^ to have the most influence without significant change in overall results (*Fig. S3*). When studies were stratified by publication year (< 2020 *versus* ≥ 2020), heterogeneity decreased slightly but remaining high for 5- and 10-year RFS (89.2% to 28.2% and 93.5% to 73.6%, respectively) (*Fig. S4*), without affecting the pooled estimates. Single-centre subgroup analysis (excluding multicentre studies^7,37^) (*Fig. S5*), as well as including only studies with low–moderate risk of bias, did not demonstrate a significant reduction in heterogeneity or affect pooled RFS estimates significantly (*Fig. S6*).

##### Predictors of recurrence

Predictors of recurrence following surgery for IPMN-derived PC were sought in all 39 articles reporting on this disease entity. Three articles reported HRs from multivariable Cox regression analyses specifically addressing this outcome measure. Three studies (1271 of 3950; 32.2%) identified both lymph node positivity and tubular histological subtype as predictors of recurrence in IPMN-derived PC and were eligible for quantitative synthesis.

**Fig. 6:**
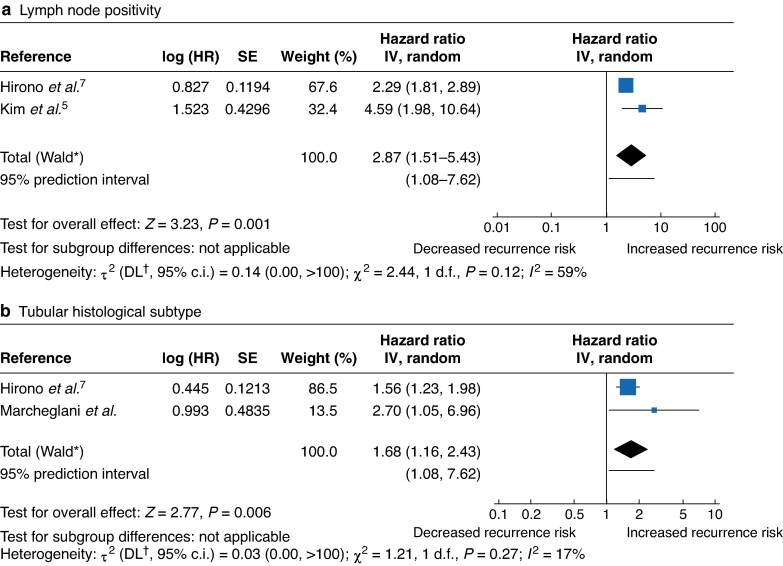
Analysis of predictors for recurrence following surgical resection of IPMN-derived PC **a** Lymph node positivity**, b** tubular histological subtype. Error bars represent 95% confidence intervals. IPMN, intraductal papillary mucinous neoplasm; PC, pancreatic cancer; HR, hazard ratio; SE, standard error; DL, DerSimonian and Laird; c.i., confidence interval; d.f., degrees of freedom. *Confidence interval calculated using Wald-type method; †τ calculated using DerSimonian and Laird method.

One study assessed overall recurrence, whereas another focused on systemic recurrence. Despite differences in outcome definitions, both were pooled to allow for quantitative synthesis, given the limited number of available studies. Meta-analysis suggested an association between lymph node metastases and a significant increase in recurrence risk (pooled HR 2.87, 95% c.i. 1.51 to 5.43; *P* = 0.001), with moderate heterogeneity (*I*^2^ = 59%) (*Fig. 6a*, *Table S12*). Similarly, pooled analysis indicated tubular IPMN carcinoma as a predictor of recurrence (pooled HR 1.68, 95% c.i. 1.16 to 2.43; *P* = 0.006, *I*^2^ = 17%) (*Fig. 6b*, *Table S12*), suggesting a more favourable outcome for the non-tubular subtypes.

#### Non-invasive IPMN

##### Patients’ characteristics

Fifty-six studies reported data on 7514 patients who underwent surgical resection for non-invasive IPMN. Their baseline characteristics are summarized in *Table S5*. For 3314 of these patients (44.1%), sex was not specified. When available, sex distribution of the study population was balanced (men 2114 of 4200; 50.3%; women 2086 of 4200; 49.7%). Median age across the included studies ranged from 45.5 (i.q.r. 38.0–50.0) years^33^ to 72.3 (i.q.r. 37.6–89.2) years^38^.

##### IPMN characteristics at time of index surgery

Cyst location was specified in 21 studies, reporting on 2103 of 7514 patients (28.0%). The most common location was the head/neck of the pancreas (1222 of 2103; 58.1%), followed by the body/tail (738; 35.1%). In 143 patients (6.8%) there was multifocal or diffuse scattered branch duct involvement. (*Table S5*).

The morphological subtype of IPMN was reported for 3997 of 7514 patients (53.2%). A total of 1011 of 3997 patients (25.3%) were diagnosed with MD-IPMN, 2102 (52.6%) with BD-IPMN, and 884 (22.1%) with MT-IPMN.

Reported cyst sizes ranged from a median of 2.2 (i.q.r. 0.2–1) cm^39^ up to 3.9 (i.q.r. 1–16.5) cm^40^. Multifocality was described in 16 articles, including a total of 2492 of 7514 patients (33.2%), of which 567 patients (22.8%) presented multifocal cysts before index surgery.

In terms of histologically determined grade of dysplasia, LGD (4907 of 7514; 65.3%) was predominant, followed by HGD (1796; 23.9%), which included 30 microinvasive dysplasia (30 of 1796; 1.7%) for which data could not be further extracted. For 811 of 7514 (10.8%) patients with non-invasive IPMN, the degree of dysplasia was not specified (*Table S5*).

##### Surgical and pathological characteristics

The type of surgical resection was specified in 32 articles for 4547 of 7514 patients (60.5%). Among these, PD was performed in 2620 patients (57.6%), LP in 979 patients (21.5%), and TP in 140 patients (3.1%). Enucleation was reported for a total of 645 patients (14.2%) and other types of resections such as partial and central pancreatectomy for 163 patients (3.6%). For the remaining 2967 patients (39.5%), the surgical approach was not specified (*Table S6*).

A total of 34 studies, involving 4550 of 7514 patients (60.6%) who underwent surgical resection for non-invasive IPMN, reported data regarding resection margin status. Among these, positive surgical margins were identified in 955 patients (21.0%), indicating the presence of residual disease at the resection margin, whereas in 3595 patients (79.0%) the surgical margins were negative (*Table S6*).

##### Recurrence

In 56 studies, reporting on 7446 of 7514 patients (99.1%) information regarding follow-up was documented. Ten articles failed to report on follow-up data for a subset of the population, totalling 68 patients (68 of 7514; 0.9%) (*Table 1*, *Table S13*). The overall recurrence rate following pancreatic resection of non-invasive IPMN was 11.2% (831 of 7446), with a median follow-up of 28 (i.q.r. 1–153) months^41^ to 114 (i.q.r. 12–204) months^39^ (*Fig. 3b*, *Table 1*, *Table S7*). In 2954 (39.7%), the patients the recurrence was confirmed histologically, whereas the diagnosis for the majority of patients (4492; 60.3%) was solely based on radiological imaging (*Table S13*).

Reclassification of recurrences (*Fig. 1*) was possible in 37 studies for a total of 582 of 831 (70.0%) patients. Development of metachronous/*de novo* IPMN was the most common type of recurrence, accounting for 408 of 831 (49.1%) recurrences, and 408 of 7,446 (5.5%) of the total follow-up population (*Fig. 3c*). The progression of a persistent cyst at the time of index surgery was documented for 174 of 831 (20.9%) reported recurrences, accounting for 174 of 7446 (2.3%) of the follow-up population (*Fig. 3c*). For the remaining 249 of 831 patients (30.0%), the recurrence type could not be reclassified, representing 249 of 7446 (3.3%) of the follow-up population (*Fig. 3c*, *Table 1*, *Table S13*).

Overall, non-invasive recurrence was more common (308 of 831; 37.1%) than IPMN-derived PC (118; 14.2%). The type of recurrence was not further specified in 405 patients (48.7%) (*Fig. 4a*, *Table S13*). In terms of location, 368 patients (44.3%) presented with locoregional and 71 (8.5%) with systemic recurrence. For the remaining 392 patients (47.2%), recurrence location was not further specified (*Fig. 4b*, *Table S13*).

Secondary treatment for disease recurrence was reported in 25 studies (265 of 831; 31.9%). Of the 265 patients, secondary surgery was performed in 131 patients (49.4%), not otherwise specified conservative treatment in 119 (44.9%), chemotherapy in 7 (2.6%), and palliative care in 5 patients (1.9%). For the remaining three patients (1.1%), secondary treatment was not further detailed (*Table S13*). The 5-year OS ranged from 85%^11^ to 100%^42^ (*Table S13*).

## Discussion

In this systematic review of available literature, a significantly higher recurrence rate was observed when IC was present at final histopathology (41.9%) than after resection of a non-invasive IPMN (11.2%). For IPMN derived-PC, the calculated pooled RFS showed a steady decline from 75.4% at 1 year to 34.0% at 10 years. Meta-analysis further suggested lymph node positivity and tubular histology as independent risk factors of recurrent disease, although these findings should be considered exploratory, given the limited number of contributing studies. Reclassification of recurrence patterns after resection for non-invasive IPMN revealed 49.1% metachronous, *de novo* cyst development and 20.9% progression of persistent disease, whereas the remaining 30.0% of the patients could not be reclassified.

The presence of IC radically worsens the long-term prognosis of IPMNs, as reflected by the markedly higher recurrence rate of IPMN-derived-PC^5,43^. From a pathophysiological standpoint, as cells progress from LGD to HGD and eventually into IC, they gain the ability to breach the ductal basement membrane and invade the surrounding pancreatic tissue. Once this occurs, cells may gain further access to vascular and lymphatic channels facilitating systemic spread of the disease^26,28^. Accordingly, lymph node involvement was identified as an independent risk factor for recurrence in this meta-analysis. Other reports^13^ previously described a 76% recurrence rate among patients with positive lymph nodes, compared with 30% in node-negative cases. Kim and colleagues^5^ indeed described nodal positivity as a predictor of metachronous distant recurrence, along with elevated CA 19-9 levels, multifocality, advanced T categories, and perineural, LVI, or venous invasion. In terms of histological features, the presence of tubular adenocarcinoma in IPMN-derived PC is considered less favourable compared with oncocytic or colloid phenotypes^12^. The tubular phenotype has been associated not only with an increased risk of disease recurrence but also with worse OS, comparable to conventional PDAC^44^. Supporting this observation, this meta-analysis indicated the tubular phenotype to be an independent risk factor for recurrence in IPMN-derived PC. Therefore, histological subtype as well as nodal involvement at final pathology are crucial for clinical decision-making and determining treatment pathways.

The possible benefit of adjuvant chemotherapy in patients with resected IPMN-derived PC is debated. Marchegiani *et al*.^36^ reported no significant improvement in disease-specific survival (DSS) compared with surgery alone in the whole patient cohort. Nonetheless, a positive effect on DSS was observed in the subset of patients who are node-positive and tubular carcinomas. Similarly, in a large retrospective multicentre study, Habib and colleagues^45^ reported a significant survival benefit of adjuvant chemotherapy solely for patients with nodal involvement or elevated CA19-9. Moreover, a recently published systematic review and meta-analysis^46^ confirmed the lack of survival benefit of adjuvant treatment in the overall population of IPMN-derived PC. However, subgroup analysis indicated a benefit for certain high-risk constellations, namely patients with positive lymph nodes and elevated CA 19-9 levels, as well as advanced T category and poor tumour differentiation. These findings underscore the biological heterogeneity of IPMN-derived PC and the resulting differences in recurrence patterns and propensity for development of metachronous systemic disease. However, this knowledge has not yet been translated into tailored treatment recommendations for IPMN-derived PC, and protocols and regimes derived from conventional PDAC are still commonly applied. Aside from IPMN-derived PC presenting with high-risk features (such as tubular type and nodal positivity), there is no clear evidence supporting a benefit of adjuvant systemic therapy, and close postoperative surveillance for disease recurrence may suffice^36,45,47^.

Regarding the duration of follow-up after resection of IPMN-derived PC, lifelong surveillance is widely recommended^6^. In support of this approach, the present analysis showed a continuous decline in the pooled RFS after the index surgery. After 10 years, the pooled RFS was only at 34.0%, supporting the concept that recurrence may occur with a long latency. However, heterogeneity was high, largely due to substantial differences in methodology and outcome reporting.

Contrary to IPMN-derived PC, non-invasive cysts showed a substantially low overall recurrence rate of 11.2%. However, this figure warrants closer examination, as the term ‘non-invasive’ covers a wide biological spectrum of lesions. As epithelium progresses from LGD to HGD, the propensity for malignant transformation increases as well. Although this process has not yet been fully understood, several key genetic drivers have been identified in the sequential progression from non-invasive epithelium to invasive carcinoma. These include KRAS and GNAS in the early, and TP53 and SMAD4 in the late phase. The duration of this process is estimated at approximately 3 years^48,49^. Accordingly, authors^5,50^ have previously reported a significant increase in recurrence risks for HGD *versus* LGD, highlighting the necessity of cyst removal before malignant progression. Accurately identifying these high-risk lesions before surgery remains a major challenge, reflected in the markedly higher proportion of LGD (65.3%) compared with HGD (23.9%) found in this systematic review following index surgery.

Aside from the various degrees of dysplasia, considering the underlying pathophysiological mechanisms is critical for evaluating recurrence risk in non-invasive IPMN. In this systemic review, the universally applied but inaccurate term ‘recurrence’ was reclassified in distinct categories, resulting in the finding that most cysts (49.1%) detected during postoperative surveillance represent newly formed, metachronous lesions. One possible explanation is a widespread epithelial susceptibility within the pancreatic ductal system, leading to the emergence of multiple, clonally unrelated lesions over time^4,36,45,51,52^. Supporting this theory, Pea and colleagues^4^ demonstrated differences in genetic alterations between the index IPMN and progressive neoplasia (either IPMN or PDAC) in the pancreatic remnant after initial margin-negative resection. In a molecular analysis of 13 patients with multifocal BD-IPMN, Matthaei *et al*.^16^ further showed substantial differences in genetic profile between lesions, suggesting the presence of a pancreatic ‘field defect’ that generates genetically distinct lesions. Such metachronous, *de novo* IPMNs developing in the remnant pancreas after margin negative resection need to be distinguished from the progression of previously existent lesions. Mechanistically, the latter are thought to be the result of residual disease found after margin positive index surgery and better termed as IPMN persistence or progression. Analysing the available literature, 20.9% of ‘recurrent’ non-invasive IPMNs were found to be more accurately described as the progression of a persisting cyst. Given the non-oncological characteristics of non-invasive IPMNs and the established pathophysiological classification into metachronous/*de novo* IPMNs and IPMN persistence based on margin status, the use of the term ‘recurrence’ warrants reconsideration. Its applicability may be limited or inappropriate in this specific context. Once the differentiation into neoplastic cells has occurred, a third possible mechanism has been described: the intraductal or intraparenchymal dissemination of lesions that are physically separate, but genetically related^4,52^. Supporting this theory, Tamura *et al*.^53^ analysed KRAS and GNAS mutation patterns, and demonstrated clonal similarities of multisegmented MD-IPMN. This monoclonal skip progression was frequently observed in malignant IPMN, giving rise to intraparenchymal metastases that can appropriately be classified as true disease recurrence. Additional support for this concept emerges from studies investigating the biology of PDAC recurrence. In a landmark genomic analysis of PDAC metastases, Makohon-Moore *et al*.^54^ demonstrated that most metachronous lesions originate from a single dominant clone of the primary tumour. In light of these findings, the term ‘recurrence’ in IPMN should be reserved for metachronous lesions that exhibit confirmed clonal relatedness to the primary neoplasm. However, the term is often applied indiscriminately, leading to potential misclassification of lesions that may instead represent independent primary tumours arising from field-defect phenomena, or progression of residual disease at the transection margin. This was reflected in the authors’ inability to reclassify the remaining 30.0% of ‘recurrences’. Future studies should adopt more standardized and precise outcome reporting to assess metachronous development and persistence rates after resection of non-invasive IPMN accurately. This will help clarify their respective prognostic significance and facilitate integration into clinical treatment guidelines.

Given recent evidence highlighting the propensity of IPMN to occur in a multifocal pattern due to a widespread epithelial susceptibility within the pancreatic ductal system, the potential need for a total pancreatectomy has been questioned^4,52^. This would arguably reduce the risk of metachronous/*de novo* IPMN, as well as disease progression of persisting lesions to a minimum^4^. Most recent multicentre data however did not reveal an OS benefit of total pancreatectomy in comparison to partial pancreatic resections in patients with multifocal or diffuse IPMN-associated cancer. The only possible exception might be represented by young and otherwise healthy patients with sufficient life expectancy to benefit from the reduced risk of local recurrence^14^. Thus, clinical decisions need to be highly individualized, balancing the potential oncologic benefit against the significant morbidity and lifelong metabolic consequences of total pancreatectomy^14^. Particularly for non-invasive, multifocal IPMN, the standard of care involves partial pancreatectomy targeting the highest-risk lesions, combined with ongoing surveillance of the remnant pancreas, as the risk of malignant transformation seems to be predominantly linked to the most advanced cyst^55,56^.

Another important variable to consider is the status of surgical margin. Once HGD or IC is found at the transection level, further resection to achieve ‘R0’ should be pursued if technically feasible, as this has shown significant impact on long-term survival^36,57^. The presence of LGD at the surgical margin on the other hand does not influence survival negatively and does not warrant further resection^58^. Accordingly, recommendations of the National Comprehensive Cancer Network, as well as international consensus guidelines, discourage prophylactic total pancreatectomy in multifocal IPMN and positive resection margins unless high-risk features persist and further resection is impossible^59^. Partial pancreatectomy, followed by ongoing surveillance of the remnant pancreas, remains the established standard of care for non-invasive IPMN.

Finally, the duration of postoperative follow-up continues to be a controversy. Notably, recurrence can still occur beyond 5 years after surgery, with a higher recurrence risk in patients with HGD, IC, or a family history of PC^3,17,18^. Given that surveillance is resource-intensive and healthcare budgets are constrained, strategies for safely discontinuing surveillance are currently under investigation. In an international multicentre study, Marchegiani and colleagues^60^ reported a risk of PDAC development similar to the general population in patients with presumed BD-IPMN without WFs or HRS and 5 years of surveillance. The authors concluded that discontinuation of surveillance could be justified after 5 years of stability in low-risk patients (age > 75 with cysts < 30 mm or age > 65 with cysts **≤** 15 mm). In contrast, Assawasirisin *et al*.^61^ report a ninefold increase in PDAC risk after 10 years of surveillance in 316 retrospectively analysed patients with BD-IPMN compared with age-matched controls. Future studies providing more robust evidence are needed to solve this controversy. The first fundamental step in this process is the standardization of pathology reporting in IPMNs, which would improve reliability and enable a more accurate stratification of the risk of recurrence.

This study has several limitations. A major challenge during data extraction was the absence of standardized and structured outcome reporting across the literature. Furthermore, a substantial proportion of studies did not differentiate outcomes between IPMN-derived PC and non-invasive IPMN. For key clinicopathological parameters, including WF or HRS, data availability was limited^62^. Marked heterogeneity was also observed in the definition of positive resection margins, and the inconsistent use of the term ‘malignancy’ further complicated outcomes interpretation. Whereas some studies classify both HGD and IC as ‘malignant’ IPMN, others restricted the term exclusively to IC^63^. Furthermore, the definition of recurrence was inconsistent among the included studies. Whereas some considered a dilated main duct as recurrence of a MD-IPMN, other interpreted this as a benign consequence of anastomotic stenosis^36,64–66^. Notably, although MPD dilatation may raise suspicion for recurrence, only 0.8% of these patients ultimately develop invasive carcinoma in the pancreatic remnant^36,64–66^. Distinguishing between recurrence due to IPMN-derived carcinoma and IPMN-concomitant carcinoma remains challenging, yet these represent distinct entities with different prognostic implications^67,68^.

In conclusion, IPMN-derived PC demonstrates a high recurrence rate after the resection, with a declining RFS over time. Adjuvant therapy and close surveillance may be warranted for high-risk lesions, such as those with lymph node involvement and tubular subtype. After pancreatectomy for non-invasive IPMN, 11.2% of patients present with new or progressive cystic disease, 49.1% of which are metachronous/*de novo* lesions. Structured reporting of recurrence, distinguishing ‘true’ recurrences, formation of metachronous/*de novo* cysts, and progression of residual disease is necessary to facilitate more accurate stratification of recurrence risk in the future. As current guidelines are evolving at a fast pace, preoperative risk assessment, clinical decision-making, and management and surveillance of IPMN are complex and should be reserved for specialized institutions offering the necessary multidisciplinary expertise to ensure optimal treatment.

## Supplementary Material

zrag087_Supplementary_Data

## Data Availability

Data supporting the findings of this study are available from the corresponding author upon reasonable request.
