# Supplementary material for: Implications and risk of new versus persisting intraductal papillary mucinous neoplasms after pancreatic surgery: meta-analysis
Source: BJS Open. 2026 Jul 13;10(4):zrag087. doi: 10.1093/bjsopen/zrag087 (PMC13361978; doi:10.1093/bjsopen/zrag087)
Supplement: zrag087_Supplementary_Data [file zrag087_supplementary_data.docx]

Rethinking Recurrence: Implications and Risk of New vs Persisting Intraductal Papillary Mucinous Neoplasms After Pancreatic Surgery

*A Systematic Review and Metanalysis*

*Anouk J. van Bodegraven^1^, *Katharina M. Marstaller-Walz MD^3^, Virginia Padoan MD^4^, Danhui Heo MD^5^, Giampaolo Perri MD^4^, George L. Burchell^1^, Martin Loos MD^3^, Christoph W. Michalski MD^3^, Marc G. Besselink MD PhD^1,2^, Umberto Cillo^4^, Ingmar F. Rompen MD^1,2,3^, Giovanni Marchegiani MD PhD ^4^

^*^These authors contributed equally to this work and share first authorship.

^1^Amsterdam UMC, location University of Amsterdam, Department of Surgery, Amsterdam, the Netherlands; ^2^Cancer Center Amsterdam, Amsterdam, the Netherlands;

^3^Department of General, Visceral, and Transplantation Surgery, Heidelberg University Hospital, Heidelberg, Germany;

^4^Department of Hepato-bilio-pancreatic Surgery and Liver Transplant Surgery, Department of Surgery, Oncology and Gastroenterology (DiSCOG), Padova University, Padova, Italy;

^5^Doctoral School of Experimental and Preventive Medicine, Albert Szent-Györgyi Medical School, University of Szeged, Hungary;

**Corresponding author**:

Giovanni Marchegiani, MD, PhD

giovanni.marchegiani@unipd.it

Hepato Biliary Pancreatic (HPB) and Liver Transplant Surgery

Padova University Hospital

Via Giustiniani 2, 35128 Padova

**ORCID ID**: 0000-0002-6824-4533

**Twitter** @Gio_Marchegiani

**Supplementary Materials-Index**

| **Supplementary Tables** |  |
| --- | --- |
| Supplementary Table 1 | *pag. 3* |
| Supplementary Table 2 | *pag. 6* |
| Supplementary Table 3  Supplementary Table 4  Supplementary Table 5  Supplementary Table 6  Supplementary Table 7  Supplementary Table 8 | *pag. 6*  *pag. 9*  *pag. 11*  *pag. 15*  *pag. 18*  *pag. 22* |
| Supplementary Table 9  Supplementary Table 10  Supplementary Table 11  Supplementary Table 12  Supplementary Table 13  Supplementary Table 14  **Supplementary Figures**  Supplementary Figure 1  Supplementary Figure 2  Supplementary Figure 3  Supplementary Figure 4  Supplementary Figure 5  Supplementary Figure 6 | *pag. 25*  *pag. 27*  *pag. 30*  *pag. 31*  *pag. 32*  *pag. 38*  *pag. 40*  *pag. 41*  *pag. 42*  *pag. 43*  *pag. 43*  *pag. 44* |
| **Supplementary References** | *pag. 45* |
|  |  |

**Supplementary Tables**

Supplementary Table 1: PRISMA 2020 Checklist.

| **1Section and Topic** | **Item #** | **Checklist item** | **Location where item is reported** |
| --- | --- | --- | --- |
| **TITLE** | | |  |
| Title | 1 | Identify the report as a systematic review. | 1 |
| **ABSTRACT** | | |  |
| Abstract | 2 | See the PRISMA 2020 for Abstracts checklist. | 3 |
| **INTRODUCTION** | | |  |
| Rationale | 3 | Describe the rationale for the review in the context of existing knowledge. | 4 |
| Objectives | 4 | Provide an explicit statement of the objective(s) or question(s) the review addresses. | 4 |
| **METHODS** | | |  |
| Eligibility criteria | 5 | Specify the inclusion and exclusion criteria for the review and how studies were grouped for the syntheses. | 5, 6, Supplementary table 2 |
| Information sources | 6 | Specify all databases, registers, websites, organisations, reference lists and other sources searched or consulted to identify studies. Specify the date when each source was last searched or consulted. | 5 |
| Search strategy | 7 | Present the full search strategies for all databases, registers and websites, including any filters and limits used. | 5, 6, Supplementary table 3 |
| Selection process | 8 | Specify the methods used to decide whether a study met the inclusion criteria of the review, including how many reviewers screened each record and each report retrieved, whether they worked independently, and if applicable, details of automation tools used in the process. | 5, 6 |
| Data collection process | 9 | Specify the methods used to collect data from reports, including how many reviewers collected data from each report, whether they worked independently, any processes for obtaining or confirming data from study investigators, and if applicable, details of automation tools used in the process. | 5, 6 |
| Data items | 10a | List and define all outcomes for which data were sought. Specify whether all results that were compatible with each outcome domain in each study were sought (e.g. for all measures, time points, analyses), and if not, the methods used to decide which results to collect. | 6, 7, Supplementary Table 4 |
|  | 10b | List and define all other variables for which data were sought (e.g. participant and intervention characteristics, funding sources). Describe any assumptions made about any missing or unclear information. | 6, 7, Supplementary Table 4 |
| Study risk of bias assessment | 11 | Specify the methods used to assess risk of bias in the included studies, including details of the tool(s) used, how many reviewers assessed each study and whether they worked independently, and if applicable, details of automation tools used in the process. | 7 |
| Effect measures | 12 | Specify for each outcome the effect measure(s) (e.g. risk ratio, mean difference) used in the synthesis or presentation of results. | 6, 7 |
| Synthesis methods | 13a | Describe the processes used to decide which studies were eligible for each synthesis (e.g. tabulating the study intervention characteristics and comparing against the planned groups for each synthesis (item #5)). | 8, 9 |
|  | 13b | Describe any methods required to prepare the data for presentation or synthesis, such as handling of missing summary statistics, or data conversions. | 8, 9 |
|  | 13c | Describe any methods used to tabulate or visually display results of individual studies and syntheses. | 8, 9 |
|  | 13d | Describe any methods used to synthesize results and provide a rationale for the choice(s). If meta-analysis was performed, describe the model(s), method(s) to identify the presence and extent of statistical heterogeneity, and software package(s) used. | 8, 9 |
|  | 13e | Describe any methods used to explore possible causes of heterogeneity among study results (e.g. subgroup analysis, meta-regression). | 8, 9 |
|  | 13f | Describe any sensitivity analyses conducted to assess robustness of the synthesized results. | 8, 9 |
| Reporting bias assessment | 14 | Describe any methods used to assess risk of bias due to missing results in a synthesis (arising from reporting biases). | 7, 8, 9 |
| Certainty assessment | 15 | Describe any methods used to assess certainty (or confidence) in the body of evidence for an outcome. | 7, 8, 9 |
| **RESULTS** | | |  |
| Study selection | 16a | Describe the results of the search and selection process, from the number of records identified in the search to the number of studies included in the review, ideally using a flow diagram. | 10, Figure 2 |
|  | 16b | Cite studies that might appear to meet the inclusion criteria, but which were excluded, and explain why they were excluded. | 10, Figure 2 |
| Study characteristics | 17 | Cite each included study and present its characteristics. | 10, 13, Supplementary table 5-8, Supplementary table 11 and 13 |
| Risk of bias in studies | 18 | Present assessments of risk of bias for each included study. | Supplementary Figure 1 |
| Results of individual studies | 19 | For all outcomes, present, for each study: (a) summary statistics for each group (where appropriate) and (b) an effect estimate and its precision (e.g. confidence/credible interval), ideally using structured tables or plots. | Supplementary table 7-10, Supplementary table 13 |
| Results of syntheses | 20a | For each synthesis, briefly summarise the characteristics and risk of bias among contributing studies. | 10, 11, 12, 13, 14, Figure 3, Figure 4 |
|  | 20b | Present results of all statistical syntheses conducted. If meta-analysis was done, present for each the summary estimate and its precision (e.g. confidence/credible interval) and measures of statistical heterogeneity. If comparing groups, describe the direction of the effect. | 12, 13, Supplementary Figure 3-6, Figure 5, Figure 6 |
|  | 20c | Present results of all investigations of possible causes of heterogeneity among study results. | 12, 13, 14, Figure 3, Figure 4, Figure 5, Figure 6 |
|  | 20d | Present results of all sensitivity analyses conducted to assess the robustness of the synthesized results. | 12, 13, Figure 5, Figure 6 |
| Reporting biases | 21 | Present assessments of risk of bias due to missing results (arising from reporting biases) for each synthesis assessed. | 12, 13 |
| Certainty of evidence | 22 | Present assessments of certainty (or confidence) in the body of evidence for each outcome assessed. | 12, 13 |
| **DISCUSSION** | | |  |
| Discussion | 23a | Provide a general interpretation of the results in the context of other evidence. | 15, 16, 17, 18 |
|  | 23b | Discuss any limitations of the evidence included in the review. | 18, 19 |
|  | 23c | Discuss any limitations of the review processes used. | 18, 19 |
|  | 23d | Discuss implications of the results for practice, policy, and future research. | 19 |
| **OTHER INFORMATION** | | |  |
| Registration and protocol | 24a | Provide registration information for the review, including register name and registration number, or state that the review was not registered. | 3, 5 |
|  | 24b | Indicate where the review protocol can be accessed, or state that a protocol was not prepared. | 3, 5 |
|  | 24c | Describe and explain any amendments to information provided at registration or in the protocol. | - |
| Support | 25 | Describe sources of financial or non-financial support for the review, and the role of the funders or sponsors in the review. | 1 |
| Competing interests | 26 | Declare any competing interests of review authors. | 1 |
| Availability of data, code and other materials | 27 | Report which of the following are publicly available and where they can be found: template data collection forms; data extracted from included studies; data used for all analyses; analytic code; any other materials used in the review. | 1 |

Supplementary Table 2: PICO-Table

| P (population) | Patients with either non-invasive IPMN or IPMN-derived invasive cancer in the pancreas |
| --- | --- |
| I (intervention) | Surgical IPMN resection |
| C (comparison) | - |
| O (outcome) | Recurrence/persistence rate |

Supplementary Table 3: **a** Overview of search terms (last updated on 25-03-2025) and search results on **b** Pubmed, **c** Embase, **d** Web of science and **e** Cochrane

**a** Overview of search terms

| **Database** | **Result** | **After deduplication** |
| --- | --- | --- |
| PubMed | 317 | 317 |
| Embase | 704 | 431 |
| Web of Science | 324 | 53 |
| Cochrane | 8 | 5 |
| **Total** | 1353 | 806 |

**b** PubMed (317)

| **Search** | **Query** | **Results** |
| --- | --- | --- |
| #4 | Search: **#1 AND #2 AND #3** | [317](https://pubmed.ncbi.nlm.nih.gov/?term=%231+AND+%232+AND+%233&ac=no&sort=relevance) |
| #3 | Search: "Pancreatic Intraductal Neoplasms"[Mesh] OR "Intraductal Papillar*"[tiab] OR "IPMN"[tiab] OR "neoplasm of the pancreas*"[tiab] OR "Pancreatic Intraductal neoplasm*"[tiab] OR "Pancreatic Intraductal tumor*"[tiab] OR "Pancreatic Intraductal tumour*"[tiab] OR "Pancreatic Intraductal cystic neoplasm*"[tiab] OR "Pancreatic Intraductal cystic tumor*"[tiab] OR "Pancreatic Intraductal cystic tumour*"[tiab] OR "Intraductal Papillary-Mucinous Neoplasm"[tiab] OR "mucinous neoplasm*"[tiab] | [7,035](https://pubmed.ncbi.nlm.nih.gov/?term=%22Pancreatic+Intraductal+Neoplasms%22%5BMesh%5D+OR%0A%0A%E2%80%9CIntraductal+Papillar%2A%E2%80%9D%5Btiab%5D+OR%0A%E2%80%9CIPMN%E2%80%9D%5Btiab%5D+OR%0A%0A%E2%80%9Cneoplasm+of+the+pancreas%2A%E2%80%9D%5Btiab%5D+OR%0A%E2%80%9CPancreatic+Intraductal+neoplasm%2A%E2%80%9D%5Btiab%5D+OR%0A%E2%80%9CPancreatic+Intraductal+tumor%2A%E2%80%9D%5Btiab%5D+OR%0A%E2%80%9CPancreatic+Intraductal+tumour%2A%E2%80%9D%5Btiab%5D+OR%0A%0A%E2%80%9CPancreatic+Intraductal+cystic+neoplasm%2A%E2%80%9D%5Btiab%5D+OR%0A%E2%80%9CPancreatic+Intraductal+cystic+tumor%2A%E2%80%9D%5Btiab%5D+OR%0A%E2%80%9CPancreatic+Intraductal+cystic+tumour%2A%E2%80%9D%5Btiab%5D+OR%0A%0A%22Intraductal+Papillary-Mucinous+Neoplasm%22%5Btiab%5D+OR%0A%E2%80%9Cmucinous+neoplasm%2A%E2%80%9D%5Btiab%5D&ac=no&sort=relevance) |
| #2 | Search: ( ("Postoperative Period"[Mesh] OR "Postoperative*"[tiab] OR "Post operative*"[tiab] OR "post surger*"[tiab] OR "after surger*"[tiab] OR "Postsurgical*"[tiab] OR "After operation*"[tiab] OR "Following surger*"[tiab] OR "After operation"[tiab]) AND (pancrea*[tiab] OR pancreaticoduodenectom*[tiab] OR pancreatectom*[tiab] OR Whipple[tiab] OR pancreatoduodenectom*[tiab] OR "tail resection" OR "distal resection"[tiab] OR "left resection"[tiab]) ) | [26,210](https://pubmed.ncbi.nlm.nih.gov/?term=%28%0A%28%22Postoperative+Period%22%5BMesh%5D+OR+%0A%22Postoperative%2A%22%5Btiab%5D+OR%0A%22Post+operative%2A%22%5Btiab%5D+OR%0A%E2%80%9Cpost+surger%2A%E2%80%9D%5Btiab%5D+OR%0A%E2%80%9Cafter+surger%2A%E2%80%9D%5Btiab%5D+OR%0A%22Postsurgical%2A%22%5Btiab%5D+OR%0A%22After+operation%2A%22%5Btiab%5D+OR%0A%22Following+surger%2A%22%5Btiab%5D+OR%0A%22After+operation%22%5Btiab%5D%29%0AAND%0A%28pancrea%2A%5Btiab%5D+OR+pancreaticoduodenectom%2A%5Btiab%5D+OR+pancreatectom%2A%5Btiab%5D+OR%0AWhipple%5Btiab%5D+OR+pancreatoduodenectom%2A%5Btiab%5D+OR+%E2%80%9Ctail+resection%E2%80%9D+OR+%E2%80%9Cdistal+resection%E2%80%9D%5Btiab%5D+OR+%E2%80%9Cleft+resection%E2%80%9D%5Btiab%5D%29+%0A%29&ac=no&sort=relevance) |
| #1 | Search: "Recurrence"[Mesh] OR "Neoplasm Recurrence, Local"[Mesh] OR Recurren*[tiab] OR Relapse*[tiab] OR Recrudescence*[tiab] OR Reoccurrence*[tiab] OR Reappearance*[tiab] OR "Return of dis*"[tiab] OR "Regrowth"[tiab] OR "Reemergence*"[tiab] OR Persisten*[tiab] OR Growth*[tiab] OR progress*[tiab] OR "Expansion"[tiab] OR "Enduring"[tiab] OR "Residual disease"[tiab] OR Proliferation[tiab] | [5,114,055](https://pubmed.ncbi.nlm.nih.gov/?term=%22Recurrence%22%5BMesh%5D+OR+%0A%22Neoplasm+Recurrence%2C+Local%22%5BMesh%5D+OR%0ARecurren%2A%5Btiab%5D+OR%0ARelapse%2A%5Btiab%5D+OR%0ARecrudescence%2A%5Btiab%5D+OR%0AReoccurrence%2A%5Btiab%5D+OR%0AReappearance%2A%5Btiab%5D+OR%0A%22Return+of+dis%2A%22%5Btiab%5D+OR%0A%E2%80%9CRegrowth%E2%80%9D%5Btiab%5D+OR%0A%22Reemergence%2A%22%5Btiab%5D+OR%0A%0A%0APersisten%2A%5Btiab%5D+OR%0AGrowth%2A%5Btiab%5D+OR%0Aprogress%2A%5Btiab%5D+OR%0A%22Expansion%22%5Btiab%5D+OR%0A%22Enduring%22%5Btiab%5D+OR%0A%22Residual+disease%22%5Btiab%5D+OR%0AProliferation%5Btiab%5D&ac=no&sort=relevance) |

**c** Embase (704)

| **Search** | **Query** | **Results** |
| --- | --- | --- |
| #5 | **#4 NOT ('chapter'/it OR 'conference abstract'/it OR 'conference paper'/it OR 'conference review'/it OR 'erratum'/it OR 'letter'/it OR 'note'/it OR 'short survey'/it)** | 704 |
| #4 | **#1 AND #2 AND #3** | 1,214 |
| #3 | 'pancreatic intraductal neoplasia'/exp OR 'intraductal papillar*':ti,ab,kw OR 'ipmn':ti,ab,kw OR 'neoplasm of the pancreas*':ti,ab,kw OR 'pancreatic intraductal neoplasm*':ti,ab,kw OR 'pancreatic intraductal tumor*':ti,ab,kw OR 'pancreatic intraductal tumour*':ti,ab,kw OR 'pancreatic intraductal cystic neoplasm*':ti,ab,kw OR 'pancreatic intraductal cystic tumor*':ti,ab,kw OR 'pancreatic intraductal cystic tumour*':ti,ab,kw OR 'intraductal papillary-mucinous neoplasm':ti,ab,kw OR 'mucinous neoplasm*':ti,ab,kw | 26,173 |
| #2 | (('postoperative period'/exp OR 'postoperative*':ti,ab,kw OR 'post operative*':ti,ab,kw OR 'post surger*':ti,ab,kw OR 'after surger*':ti,ab,kw OR 'postsurgical*':ti,ab,kw OR 'after operation*':ti,ab,kw OR 'following surger*':ti,ab,kw OR 'after operation':ti,ab,kw) AND ('pancrea*':ti,ab,kw OR 'pancreaticoduodenectom*':ti,ab,kw OR 'pancreatectom*':ti,ab,kw OR 'whipple':ti,ab,kw OR 'pancreatoduodenectom*':ti,ab,kw OR 'tail resection' OR 'distal resection':ti,ab,kw OR 'left resection':ti,ab,kw)) | 49,144 |
| #1 | 'recurrent disease'/exp OR 'tumor recurrence'/exp OR 'recurren*':ti,ab,kw OR 'relapse*':ti,ab,kw OR 'recrudescence*':ti,ab,kw OR 'reoccurrence*':ti,ab,kw OR 'reappearance*':ti,ab,kw OR 'return of dis*':ti,ab,kw OR 'regrowth':ti,ab,kw OR 'reemergence*':ti,ab,kw OR 'persisten*':ti,ab,kw OR 'growth*':ti,ab,kw OR 'progress*':ti,ab,kw OR 'expansion':ti,ab,kw OR 'enduring':ti,ab,kw OR 'residual disease':ti,ab,kw OR 'proliferation':ti,ab,kw | 6777,900 |

**d** Web of Science (324)

| **Search** | **Query** | **Results** |
| --- | --- | --- |
| #4 | **#1 AND #2 AND #3** | 324 |
| #3 | TS=( “intraductal papillar*” OR “ipmn” OR “neoplasm of the pancreas*” OR “pancreatic intraductal neoplasm*” OR “pancreatic intraductal tumor*” OR “pancreatic intraductal tumour*” OR “pancreatic intraductal cystic neoplasm*” OR “pancreatic intraductal cystic tumor*” OR “pancreatic intraductal cystic tumour*” OR “intraductal papillary-mucinous neoplasm” OR “mucinous neoplasm*”) | 9,212 |
| #2 | TS=(  (“postoperative*” OR “post operative*” OR “post surger*” OR “after surger*” OR “postsurgical*” OR “after operation*” OR “following surger*” OR “after operation”)  AND  (“pancrea*” OR “pancreaticoduodenectom*” OR “pancreatectom*” OR “whipple” OR “pancreatoduodenectom*” OR “tail resection” OR “distal resection” OR “left resection”)  ) | 23,904 |
| #1 | TS=(“recurren*” OR “relapse*” OR “recrudescence*” OR “reoccurrence*” OR “reappearance*” OR “return of dis*” OR “regrowth” OR “reemergence*” OR “persisten*” OR “growth*” OR “progress*” OR “expansion” OR “enduring” OR “residual disease” OR “proliferation”) | 8,796,101 |

**e** Cochrane (8)

| **Search** | **Query** | **Results** |
| --- | --- | --- |
| #4 | **#1 AND #2 AND #3** | 8 |
| #3 | (intraductal NEXT papillar* OR ipmn OR neoplasm NEXT of NEXT the NEXT pancreas* OR pancreatic NEXT intraductal NEXT neoplasm* OR pancreatic NEXT intraductal NEXT tumor* OR pancreatic NEXT intraductal NEXT tumour* OR pancreatic NEXT intraductal NEXT cystic NEXT neoplasm* OR pancreatic NEXT intraductal NEXT cystic NEXT tumor* OR pancreatic NEXT intraductal NEXT cystic NEXT tumour* OR intraductal NEXT papillary-mucinous NEXT neoplasm OR mucinous NEXT neoplasm*):ti,ab,kw | 139 |
| #2 | (  (postoperative* OR post NEXT operative* OR post NEXT surger* OR after NEXT surger* OR postsurgical* OR after NEXT operation* OR following NEXT surger* OR after NEXT operation)  AND  (pancrea* OR pancreaticoduodenectom* OR pancreatectom* OR whipple OR pancreatoduodenectom* OR tail NEXT resection OR distal NEXT resection OR left NEXT resection)  ):ti,ab,kw | 3550 |
| #1 | (recurren* OR relapse* OR recrudescence* OR reoccurrence* OR reappearance* OR return NEXT of NEXT dis* OR regrowth OR reemergence* OR persisten* OR growth* OR progress* OR expansion OR enduring OR residual NEXT disease OR proliferation):ti,ab,kw | 321880 |

Supplementary Table 4: Detailed overview of the recorded variables

| **Domain** | **Variable** | **Details** |
| --- | --- | --- |
| **Baseline Characteristics** | Sex | Male / Female, number of patients (%) |
|  | Age at time of surgery | Years (mean ± SD) |
|  | Index surgery for IPMN | Non-invasive vs IPMN-derived PC, number of patients |
| — If malignant |  | Categorize as high-grade dysplasia (HGD, non-invasive) or invasive carcinoma (IC), or malignant if not specific |
| — For non-invasive IPMN |  | Categorize as low-grade dysplasia (LGD) or HGD, reclassify intermediate-grade dysplasia (IGD) as LGD (Figure 1) |
|  | IPMN location | Head/neck, Body/tail, other |
|  | Morphological type | Main-duct / Mixed-type / Branch-duct IPMN |
|  | IPMN cyst size | In centimeters |
|  | Invasive tumor size | In centimeters |
|  | Multifocality | Number of patients (%) |
|  | Worrisome features / High-risk stigmata | Based on Kyoto guidelines at time of index surgery |
| **Surgery Characteristics** |  |  |
|  | Type of pancreatic resection | Pancreatoduodenectomy (PD), Left pancreatectomy (LP), Total pancreatectomy (TP), Non-oncological (if non-invasive; enucleation, limited resections, partial pancreatectomy), others (central/middle pancreatectomy) |
| — For IPMN derived PC | Adjuvant therapy | Number of patients (%)  Specify type (radio- and/or chemotherapy) |
| **Final Histopathology** |  |  |
|  | Highest grade of dysplasia | LGD, HGD, micro-invasive IPMN, IC |
|  | IPMN cyst size | In centimeters |
| — For IPMN-derived PC | IPMN subtype | Colloid, Tubular, Oncocytic, not specified |
|  | TNM stage | Pathologic TNM classification |
|  | Number of resected lymph nodes | Mean absolute number ±SD |
|  | \| Number of positive lymph nodes \| \| --- \| | (mean ± SD) |
|  | \| Margin status \| \| --- \| | Number of patients (%), positive margin (>LGD on margin) |
|  | \| Lymphovascular invasion (LVI) \| \| --- \| | Number of patients (%) |
|  | Perineural invasion (PNI) | Number of patients (%) |
| **Follow-Up** |  |  |
|  | Median follow-up time | In months (IQR) |
|  | Method of follow-up | Radiologic imaging (CT, EUS, MRI) and/or Histologic confirmation and/or Serum Tumor markers |
|  | Follow-up population | Number of patients (% of study population) |
|  | Recurrence | Number of patients (%) |
|  | Recurrence interval | Median months from surgery to recurrence (IQR) |
|  | Recurrence location | Locoregional (extra-pancreatic or remnant), distant or other |
|  | Recurrence type | Non-invasive IPMN, IPMN derived PC, other |
|  | Secondary therapy post-recurrence | Surgery, Chemotherapy, Palliative care, Observation, other |
|  | Histopathology after secondary resection | Grade of dysplasia, IPMN cyst size (in millimeters), TNM-stage, IPMN subtype |
|  | Overall survival | % or median |
|  | Disease free survival | % or median |

SD, standard deviation; IPMN, intraductal papillary mucinous neoplasm; IQR, interquartile range; CT, Computed Tomography; EUS, Endoscopic UltraSonography; MRI , Magnetic Resonance Imaging

Supplementary Table 5: Overall results for both non-invasive and IPMN derived PC (n = number of patients)

| Study | Study design | Study period | Study setting | Sample size  non-invasive IPMN (n) | Sample size  IPMN-derived PC (n) |
| --- | --- | --- | --- | --- | --- |
| \| Abdalla, 2024 ^1^ | retrospective | 2000 - 2021 | single center |  | 217 |
| \| Al Efishat, 2018 ^2^ | retrospective | 1989 - 2015 | single center | 319 |  |
| \| Aleotti, 2022 ^3^ | retrospective | 01/2009 - 12/2018 | single center | 133 |  |
| \| Amini, 2022 ^4^ | retrospective | 01/1995 - 01/2018 | single center | 449 |  |
| \| Antoñanzas J, 2018 ^5^ | retrospective | 08/1993 - 12/2016 | single center | 18 |  |
| \| Blackham, 2017 ^6^ | retrospective | 1999 - 2015 | single center | 100 |  |
| \| Blair, 2022 ^7^ | retrospective | 2004 - 2016 | single center | 127 |  |
| \| Chari, 2002 ^8^ | retrospective | 1987 - 2001 | single center | 73 | 40 |
| \| Chunhui, 2014 ^9^ | retrospective | 05/2001 - 07/2011 | single center | 24 | 12 |
| \| Crippa, 2010 ^10^ | retrospective | 01/1988 - 12/2006 | multi center | 271 | 118 |
| \| Cuillerier, 2000 ^11^ | retrospective | 1980 - 1996 | multi center | 26 | 19 |
| \| Dhar, 2018^12^ | retrospective | Not reported (before 2018) | single center | 330 |  |
| \| Faitot, 2015 ^13^ | retrospective | 1998 - 2011 | single center | 38 |  |
| \| Frankel, 2013 ^14^ | retrospective | 1990 – 2010 | single center | 192 |  |
| \| Fuji, 2011 ^15^ | retrospective | 07/1991 - 12/2009 | single center | 84 | 30 |
| \| Fuji, 2022 ^16^ | retrospective | 05/2007 - 12/2014 | single center | 61 |  |
| \|Habib, 2022 ^17^ | retrospective | 1996 – 2018 | single center |  | 213 |
| \| Hirono, 2016 ^18^ | retrospective | 07/1999 - 03/2014 | single center | 172 | 85 |
| \| Hirono, 2020 ^19^ | retrospective | 01/1996 - 12/2014 | multi center | 827 | 247 |
| \| Hirono, 2024 ^20^ | retrospective | 1996 - 12/2018 | multi center |  | 1143 |
| \| Jang, 2016 ^21^ | retrospective | 01/2004 - 06/2014 | single center | 63 | 11 |
| \| Kaiser, 2022 ^22^ | retrospective | 2004 – 2014 | single center | 74 |  |
| \| Kaiser, 2023 ^23^ | retrospective | 2004 - 09/2020 | single center | 59 | 19 |
| \| Kamata, 2018 ^24^ | retrospective | 04/2009 - 03/2015 | single center | 92 | 42 |
| \| Kang, 2014 ^25^ | retrospective | Since 1995 | single center | 298 | 68 |
| \| S. Kim, 2008 ^26^ | retrospective | 08/1994 - 12/2004 | single center | 82 | 28 |
| \| H. Kim, 2022 ^27^ | retrospective | 2000 – 2018 | single center | 431 | 117 |
| \| M. Kim, 2024 ^28^ | retrospective | 01/2010 - 12/2019 | single center | 133 | 118 |
| \| R. Kim, 2024 ^29^ | retrospective | 2013 – 2018 | multi center | 481 |  |
| \| Kimura, 2017 ^30^ | retrospective | 1994 – 2015 | single center | 71 | 27 |
| \| Kurahara, 2015 ^31^ | retrospective | 01/1998 - 09/2013 | single center |  | 11 |
| \| Li, 2020 ^32^ | retrospective | 01/2013 - 06/2019 | multi center | 125 | 93 |
| \| Lubezky, 2010 ^33^ | retrospective | 2002 - 2008 | single center | 39 |  |
| \| Lucocq, 2024 ^34^ | retrospective | 2010 – 2020 | multi center |  | 415 |
| \| Majumder, 2019 ^35^ | retrospective | 1997 – 2014 | single center | 138 |  |
| \| Marchegiani, 2015 (Oncocytic) ^36^ | retrospective | 03/1990 - 01/2013 | single center |  | 11 |
| \| Marchegiani, 2015 (patterns) ^37^ | retrospective | 08/1990 - 01/2013 | single center | 316 | 84 |
| \| Marchegiani, 2019 ^38^ | retrospective | 1990 – 2016 | single center |  | 102 |
| \| Miller, 2011 ^39^ | retrospective | 09/1991 - 01/2010 | single center | 191 |  |
| \| Nagai, 2008 ^40^ | retrospective | 01/1984 - 05/2006 | single center | 42 | 30 |
| \| Nakagohri, 2002 ^41^ | retrospective | 11/1982 - 12/1997 | single center | 16 |  |
| \| Nakagohri, 2007 ^42^ | retrospective | 04/1994 - 10/2006 | single center | 14 | 37 |
| \| Nakagohri, 2010 ^43^ | retrospective | 07/1994 - 01/2007 | multi center | 13 |  |
| \| Niedergethmann, 2008 ^44^ | retrospective | 1996 – 2006 | multi center | 29 | 68 |
| \| Park, 2011 ^45^ | retrospective | 03/1995 - 07/2009 | single center | 68 | 35 |
| \| Partelli, 2010 ^46^ | retrospective | 01/1990 - 12/2006 | multi center |  | 104 |
| \| Pea, 2017 ^47^ | retrospective | 01/1996 - 01/2014 | single center | 260 |  |
| \| Pfluger, 2022 ^48^ | retrospective | 1995 – 2009 | single center | 126 |  |
| \| Ridtitid, 2016 ^49^ | retrospective | 2001 – 2013 | single center | 117 |  |
| \| Salvia, 2004 ^50^ | retrospective | 1990 – 2002 | multi center | 57 | 58 |
| \| Sauvanet, 2014 ^51^ | retrospective | 01/1999 - 06/2011 | single center | 77 |  |
| \| Schnelldorfer, 2008 ^52^ | retrospective | 1992 – 2005 | single center | 145 | 63 |
| \| Stauffer, 2009 ^53^ | retrospective | 01/2002 - 01/2008 | single center | 21 |  |
| \| Sugimachi, 2021 ^54^ | retrospective | 2005 – 2020 | single center | 35 | 15 |
| \| Sugiyama, 2003 ^55^ | retrospective | Not reported (before 2001) | single center | 19 |  |
| \| Takahashi, 2006 ^56^ | retrospective | 03/1992 - 01/2004 | single center | 20 |  |
| \| Tamura, 2014 ^57^ | retrospective | 1987 - 2012 | single center | 36 | 19 |
| \| Thomas, 2015 ^58^ | retrospective | 01/2008 - 03/2013 | single center | 17 |  |
| \| Wada, 2005 ^59^ | retrospective | 1998 - 2003 | single center | 75 | 25 |
| \| Winner, 2013 ^60^ | retrospective | 1994 - 2011 | single center | 155 | 28 |
| \| Woo, 2008 ^61^ | retrospective | 01/1999 - 08/2006 | single center |  | 19 |
| \| Xourafas, 2015 ^62^ | retrospective | 07/2002 - 2012 | single center | 87 |  |
| \| Yamaguchi, 2016 ^63^ | retrospective | 10/2004 - 12/2013 | single center | 45 |  |
| \| Yamashige, 2025 ^64^ | retrospective | 01/1996 - 12/2024 | multi center |  | 136 |
| \| Yogi, 2015 ^65^ | retrospective | 1988 - 2014 | single center | 118 | 28 |
| \| Yokoyama, 2007 ^66^ | retrospective | 10/1979 - 09/2005 | single center | 85 | 15 |
|  |  |  |  | 7,514 | 3,950 |

Supplementary Table 6: Demographics and morphology of patients undergoing resection for non-invasive IPMN (N = number, % = percentage, y = year, IQR = interquartile range, cm = centimeter, nr = not reported,)

| Study | Total sample size non-invasive, N | Sample size, N (%) | | | Sex, N (%) | | Median age, y (IQR) | ASA-score | IPMN location, N (%) | | | Morphological subtype, N (%) | | | IPMN cyst size, cm | Multi-focality, N (%) |
| --- | --- | --- | --- | --- | --- | --- | --- | --- | --- | --- | --- | --- | --- | --- | --- | --- |
|  |  | LGD | HGD | Other | M | F |  |  | Head/  neck | Body/  Tail | Other | MD-IPMN | BD-IPMN | Mixed IPMN |  |  |
| Al Efishat, 2018 ^2^ | 319 | 203 (63.6) | 94 (29.5) | 19 microinvasive  3 NR | 150 (47) | 169 (53) | 68 | nr | 213 (66.7) | 100 (31.3) | 6 (1.8) nr | 116 (37.5) | 166 (52.5) | 34 (11) | mean 2.9 (IQR 0.7-9.2) | nr |
| Aleotti, 2022 ^3^ | 133 | 133 (100) | 0 (0) | 0 (0) | 71 (53.4) | 62 (46.6) | 68 (38-83) | nr | 67 (50.4) | 52 (39.1) | 14 (10.5) diffuse | nr | nr | nr | nr | nr |
| Amini, 2022 ^4^ | 449 | 319 (71.1) | 130 (28.9) | 0 (0) | 216 (48.1) | 233 (51.9) | 69 (61-75) | nr | nr | nr | nr | 57 (12.7) | 231 (51.5) | 161 (35.8) | Median 2.6 (IQR 1.8-3.6) | 164 (40.9) |
| Antoñanzas J, 2018 ^5^ | 18 | 18 (100) | 0 (0) | 0 (0) | 11 (61) | 7 (39) | 60 (55-72) | 12 (67) I/II; 6 (33) III/IV | 12 (67) | 4 (22) | 2 (11) diffuse | 1 (6) | 10 (56) | 7 (39) | nr | nr |
| Blackham, 2017 ^6^ | 100 | 0 (0) | 100 (100) | 0 (0) | 50 (50) | 50 (50) | 72.3 (37.6-89.2) | nr | 64 (64) | 26 (26) | 10 (16) nr | 15 (15) | 58 (58) | 27 (27) | Median 2.5 (IQR 1-6.1) | 50 (50) |
| Blair, 2022 ^7^ | 127 | 78 (61) | 49 (39) | 0 (0) | 55 (43) | 61 (48) | 73 [67–79] 67[60–75] | nr | nr | nr | 47 diffuse (37)  80 nr | 37(29) | 0 (0) | 90 (71) | nr | nr |
| Chari, 2002 ^8^ | 73 | 56 (76.7) | 17 (23.3) | 0 (0) | 38 (52.1) | 35 (47.9) | 64 | nr | nr | nr | nr | nr | nr | nr | nr | nr |
| Chunhui, 2014 ^9^ | 24 | 18 (75) | 6 (25) | 0 (0) | nr | nr | nr | nr | nr | nr | nr | 7 (29.2) | 8 (33.3) | 9 (37.5) | nr | nr |
| Crippa, 2010 ^10^ | 271 | 271 (100) | 0 (0) | 0 (0) | nr | nr | nr | nr | nr | nr | nr | 42 (15.5) | 142 (52.4) | 87 (32.1) | nr | nr |
| Cuillerier, 2000 ^11^ | 26 | nr | nr | 26 nr | nr | nr | 63 (35-81) | nr | nr | nr | nr | nr | nr | nr | nr | 1 (3.8) |
| Dhar, 2018 ^12^ | 330 | 265 (80.3) | 65 (19.7) | 0 (0) | 162 (49.1) | 168 (50.9) | 67 (59-73) | 3 (II-IV) | 192 (56.1) | 100 (30.3) | 38 (11.5) | 115 (40.1) | 98 (34.1) | 74 (25.8) | nr | 78 (24.9) |
| Faitot, 2015 ^13^ | 38 | nr | nr | 38 nr | nr | nr | nr | nr | nr | nr | nr | 0 | 38 (100) | 0 | nr | nr |
| Frankel, 2013 ^14^ | 192 | 132 (68.8) | 60 (31.3) | 0 (0) | 86 (45) | 106 (55) | 68 | nr | nr | nr | nr | 48 (25) | 94 (49) | 50 (26) | Mean 2.7 (SD ±2.3) | 10 (5.2) |
| Fuji, 2011 ^15^ | 84 | nr | nr | 84 nr | nr | nr | nr | nr | 84 (100) | 0 (0) | 0 (0) | nr | nr | nr | nr | nr |
| Fuji, 2022 ^16^ | 61 | 42 (68.8) | 19 (31.2) | 0 (0) | nr | nr | nr | nr | nr | nr | nr | nr | nr | nr | nr | nr |
| Hirono, 2016 ^18^ | 172 | 85 (33.1) | 87 (33.8) | 0 (0) | nr | nr | nr | nr | nr | nr | nr | nr | nr | nr | nr | nr |
| Hirono, 2020 ^19^ | 827 | 487 (58.9) | 340 (41.4) | 0 (0) | nr | nr | 69 (26-90) | nr | nr | nr | nr | nr | nr | nr | nr | nr |
| Jang 2016 ^21^ | 63 | 42 (79.2) | 21 (39.6) | 0 (0) | nr | nr | nr | nr | Nr | nr | nr | nr | nr | nr | nr | nr |
| Kaiser, 2022 ^22^ | 74 | 71 (96.0) | 3 (4.0) | 0 (0) | 22 (37) | 52 (64) | 63 (56.3-71) | nr | 44 (59.5) | 19 (25.7) | 11 (14.9) multifocal | nr | 74 (100) | nr | nr | 11 (14.9) |
| Kaiser, 2023 ^23^ | 59 | 43 (72.9) | 16 (27.1) | 0 (0) | 25 (42.4) | 34 (57.6) | 45.5 (38-50) | nr | 35 (59.3) | 16 (27.1) | 8 multifocal  (13.5) | 6 (10.2) | 28 (47.7) | 25 (42.4) | nr | 8 (13.6) |
| Kamata, 2018 ^24^ | 92 | 56 (42) | 36 (27) | 0 (0) | 69 (51) | 65 (49) | 69.8 | nr | nr | nr | nr | nr | nr | nr | nr | nr |
| Kang, 2014 ^25^ | 298 | 253 (84.9) | 45 (15.1) | 0 (0) | nr | nr | 63.7 | nr | nr | nr | nr | nr | nr | nr | Mean 3.1 (SD ± 1.4) | nr |
| S. Kim, 2008 ^26^ | 82 | 74 (90.2) | 8 (9.7) | 0 (0) | nr | nr | nr | nr | nr | nr | nr | nr | nr | nr | Mean 3.9 (SD±2.7) | nr |
| H. Kim, 2022 ^27^ | 431 | 353 (82.0) | 78 (18.1) | 0 (0) | nr | nr | nr | nr | 207 (48.1) | 210 (48.7) | 14 diffuse  (3.2) | 29 (6.7) | 279 (64.7) | 123 (28.5) | nr | 57 (13.2) |
| M. Kim, 2024 ^28^ | 133 | 133 (100) | 0 (0) | 0 (0) | 86 (64.6) | 47 (35.3) | 71.8 (41-88) | nr | 74 (55.6) | 59 (44.4) | 0 (0) | 8 (6) | 124 (93.2) | 1 (0.7) | nr | 48 (36.1) |
| R. Kim, 2024 ^29^ | 481 | 285 (59.2) | 196 (40.7) | 0 (0) | 268 (55.7) | 213 (44.3) | 66 | nr | nr | nr | nr | 274 (56.9) | 207 (40) | 0 (0) | nr | nr |
| Kimura 2017 ^30^ | 71 | 58 (81.7) | 13 (18.3) | 0 (0) | 38 (53.5) | 33 (46.5) | 67 | nr | 27 (38) | 40 (56.3) | 4 (5.6) diffuse | 14 (19.7) | 42 (59.1) | 15 (21.2) | nr | 4 (5.6) |
| Li 2020 ^32^ | 125 | nr | nr | 125 nr | nr | nr | 63 (56-70) | nr | nr | nr | nr | 59 (47.2) | 21 (16.8) | 45 (36) | nr | nr |
| Lubezky, 2010 ^33^ | 39 | 39 (100) | 0 (0) | 0 (0) | nr | nr | nr | nr | nr | nr | nr | nr | nr | nr | nr | nr |
| Majumder, 2019 ^35^ | 138 | 112 (81.2) | 26 (18.8) | nr | 74 (53.6) | 64 (46.4) | 68.7 | nr | nr | nr | nr | nr | nr | nr | nr | nr |
| Marchegiani, 2015  patterns ^37^ | 316 | nr | nr | 316 nr | 140 (44.3) | 176 (55.7) | 67 (39-88) | nr | nr | nr | nr | nr | nr | nr | nr | nr |
| Miller, 2011 ^39^ | 191 | 162 (84.8) | 29 (15.2) | 0 (0) | 94 (49) | 97 (51) | 68 | 2.9 (2-4) | nr | nr | nr | nr | nr | nr | Mean 2.2 | nr |
| Nagai, 2008 ^40^ | 42 | 28 (66.7) | 14 (33.3) | 0 (0) | nr | nr | nr | nr | nr | nr | nr | 5 (11.9) | 31 (73.8) | 6 (14.3) | nr | nr |
| Nakagohri, 2002 ^41^ | 16 | nr | nr | 16 nr | 11 (68.7) | 5 (31,3) | 61 (44-76) | nr | 11 (68.7) | 5 (31.3) | 0 | 0 | 6 (37.5) | 10 (62.5) | Mean 2.5 (0.8-4) | nr |
| Nakagohri, 2007 ^42^ | 14 | nr | nr | 14 nr | nr | nr | nr | nr | nr | nr | nr | 6 (48.2) | 8 (57.1) | 0 (0) | nr | nr |
| Nakagohri, 2010 ^43^ | 13 | 10 (77) | 3 (23) | 0 (0) | 11 (84.6) | 2 (15.4) | nr | nr | 13 (100) | 0 (0) | 0 (0) | 0 (0) | 13 (100) | 0 (0) | Mean 3.1 (1-6.4) | nr |
| Niedergethman, 2008 ^44^ | 29 | nr | nr | 29 nr | nr | nr | nr | nr | nr | nr | nr | nr | nr | nr | Median 2.8 (IQR 0.7-8.8) | nr |
| Park, 2011 ^45^ | 68 | nr | nr | 68 nr | 76 (73.8) | 27 (26.2) | 61.1 (32-77) | nr | nr | nr | nr | nr | nr | nr | Median 3.9 (IQR 1 - 16.5) | nr |
| Pea, 2017 ^47^ | 260 | 176 (67.7) | 84 (32.3) | 0 (0) | 131 (50.4) | 129 (49.6) | 69 (61-76) | nr | nr | nr | nr | 0 | 161 (62) | 0 (0) | 2 (1.5-3) | 35 (13.5) |
| Pfluger, 2022 ^48^ | 126 | 83 (65.9) | 41 (32.5) | 2 (1.6) | 64 (50.8) | 62 (49.2) | 69 (36-90) | nr | nr | nr | nr | 24 (19.0) | 10 (7.9) | 92 (73.0) | Median 2.2 (0.2-1.0) | 50 (39.7) |
| Ridtitid, 2016 ^49^ | 117 | 117 (100) | 0 (0) | 0 (0) | nr | nr | nr | nr | nr | nr | nr | 0 | 117 (100) | 0 (0) | Mean 2.7 (± 1.3) | nr |
| Salvia 2004 ^50^ | 57 | 57 (100) | 0 (0) | 0 (0) | nr | nr | 67.3 | nr | 28 (49.1) | 8 (14.0) | 21 diffuse (36.8) | 57 (100) | 0 (0) | 0 (0) | nr | 21 (36.8) |
| Sauvanet, 2014 ^51^ | 77 | nr | nr | 2 microinvasive (2.6)  75 nr | 40 (52) | 37 (48) | 59 (48-66) | nr | 36 (47) | 41 (53) | 0 (0) | 5 (7) | 54 (70) | 18 (23) | Median 2.5 (IQR 1.5-3) | 15 (19) |
| Schnelldorfer, 2008 ^52^ | 145 | 126 (86.9) | 19 (13.1) | 0 (0) | nr | nr | nr | nr | nr | nr | nr | nr | nr | nr | Mean 3.9 (SD± 0.3) | nr |
| Stauffer, 2009 ^53^ | 21 | 11 (52.4) | 10 (47.6) | 0 (0) | 4 (19) | 17 (81) | 71 (43-78) | 10 II 10 III 1 IV | nr | nr | nr | nr | nr | nr | nr | nr |
| Sugimachi, 2021 ^54^ | 35 | 23 (65.7) | 12 (34.3) | 0 (0) | nr | nr | nr | nr | 17 (48.6) | 12 (34.3) | 6 (17.1) diffuse | 11 (31.4) | 14 (40) | 10 (28.6) | nr | nr |
| Sugiyama, 2003 ^55^ | 19 | 13 (68.4) | 6 (31.6) | 0 (0) | 13 (59.1) | 9 (40.9) | 60 (46-77) | nr | nr | nr | nr | nr | nr | nr | nr | nr |
| Takahashi, 2006 ^56^ | 20 | 13 (65) | 4 (20) | 3 micro invasive (15) | 15 (75) | 5 (25) | 64 (42-76) | nr | 13 (65) | 6 (30) | 1 (5) diffuse | 3 (15) | 17 (85) | 0 (0) | Mean 3 (0.7-5) | 2 (10) |
| Tamura, 2014 ^57^ | 36 | 20 (55.6) | 16 (44.4) | 0 (0) | 20 (55.6) | 16 (44.4) | 69.4 | nr | 17 (47.2) | 17 (47.2) | 2 (5,5) diffuse | 36 (100) | 0 (0) | 0 (0) | nr | nr |
| Thomas, 2015 ^58^ | 17 | nr | nr | 17 nr | 5 (29.4) | 12 (70.6) | 64 | nr | 12 (70.6) | 5 (29.4) | 0 (0) | nr | nr | nr | Mean 3.1 | nr |
| Wada 2005 ^59^ | 75 | 61 (81.3) | 14 (18.7) | 0 (0) | 34 (45) | 41 (55) | 64.9 | nr | nr | nr | nr | nr | nr | nr | nr | nr |
| Winner, 2013 ^60^ | 155 | 121 (78.1) | 34 (21.9) | 0 (0) | nr | nr | nr | nr | nr | nr | nr | nr | nr | nr | nr | nr |
| Xourafas, 2015 ^62^ | 87 | 69 (79.3) | 18 (20.7) | 0 (0) | 35 (41) | 52 (59) | 67 (34-90) | nr | 56 (64.3) | 18 (20.7) | 13 (15) diffuse | 36 (41.2) | 51 (58.8) | 0 (0) | Median 2.5 (IQR 0.4-7) | 13 (15) |
| Yamaguchi, 2016 ^63^ | 45 | 40 (88.9) | 1 (2.2) | 4 (8.8) | nr | nr | nr | nr | nr | nr | nr | nr | nr | nr | nr | nr |
| Yogi, 2015 ^65^ | 118 | 84 (54.9) | 34 (22.2) | 0 (0) | nr | nr | nr | nr | nr | nr | nr | nr | nr | nr | nr | nr |
| Yokoyama, 2007 ^66^ | 85 | 67 (78.8) | 18 (21.2) | 0 (0) | nr | nr | nr | nr | nr | nr | nr | nr | nr | nr | nr | nr |

Supplementary Table 7: Surgical characteristics and final histopathology of patients undergoing resection for non-invasive IPMN (n = number of patients, % = percentage, cm = centimeter, LGD = low grade dysplasia, HGD = high grade dysplasia, Margin+ = positive surgical resection margin (≥LGD), nr = not reported)

| Study | Type of resection, n (%) | | | | | Highest grade of dysplasia | Margin+, n (%) |
| --- | --- | --- | --- | --- | --- | --- | --- |
|  | PD | LP | TP | Enucleation | Partial |  |  |
| Al Efishat, 2018 ^2^ | 203 (63.6) | 89 (27.9) | nr | 11 (5.4) |  | microinvasive | 169 (53) |
| Aleotti, 2022 ^3^ | 64 (48.1) | 51 (38.3) | 14 (10.5) | 3 (2.3) | 1 (0.7) | LGD | nr |
| Amini, 2022 ^4^ | 290 (64.6) | 132 (29.4) | 0 (0) | 0 (0) | 0 (0) | HGD | 80 (17.8) |
| Antoñanzas J, 2018 ^5^ | 7 (39) | 2 (11) | 5 (28) | 1 (6) | 3 (17) | LGD | 4 (22.2) |
| Blackham, 2017 ^6^ | 44 (44) | 30 (30) | 14 (14) | 0 (0) | 12 (12) | HGD | 33 (33) |
| Blair, 2022 ^7^ | 90 (71) | 37 (29) | nr | nr | nr | HGD | 30 (24) |
| Chari 2002 ^8^ | 35 (47.9) | 24 (32.9) | 13 (17.8) | 0 (0) | 0 (0) | HGD | 2 (2.7) |
| Chunhui, 2014 ^9^ | nr | nr | nr | nr | nr | HGD | nr |
| Crippa 2010 ^10^ | nr | nr | nr | nr | nr | LGD | nr |
| Cuillerier, 2000 ^11^ | 0 (0) | 0 (0) | 6 (23.1) | 0 (0) | 20 (76.9) | nr | 7 (26.9) |
| Dhar, 2018 ^12^ | 204 (61.8) | 99 (30) | 16 (4.8) | 1 (0.3) | 10 (3) | HGD | 65 (19.7) |
| Faitot, 2015 ^13^ | 0 (0) | 0 (0) | 0 (0) | 38 (100) | 0 (0) | nr | nr |
| Frankel, 2013 ^14^ | 125 (65.1) | 53 (27.6) | 0 (0) | 14 (7.3) | 0 (0) | HGD | 38 (19.8) |
| Fuji, 2011 ^15^ | 84 (100) | nr | nr | nr | nr | nr | nr |
| Fuji, 2022 ^16^ | nr | nr | nr | nr | nr | HGD | 28 (30.8) |
| Hirono, 2016 ^18^ | nr | nr | nr | nr | nr | HGD | 79 (45.9) |
| Hirono, 2020 ^19^ | nr | nr | nr | nr | nr | HGD | nr |
| Jang 2016 ^21^ | nr | nr | nr | nr | nr | HGD | nr |
| Kaiser, 2022 ^22^ | 0 (0) | 0 (0) | 0 (0) | 74 (100) | 0 (0) | HGD | 0 (0) |
| Kaiser, 2023 ^23^ | 32 (54.2) | 8 (13.5) | 5 (8.4) | 13 (22.0) | 1 (1.7) | HGD | 6 (10.2) |
| Kamata, 2018 ^24^ | nr | nr | nr | nr | nr | HGD | nr |
| Kang, 2014 ^25^ | nr | nr | nr | nr | nr | HGD | nr |
| S. Kim, 2008 ^26^ | nr | nr | nr | nr | nr | HGD | nr |
| H. Kim, 2022 ^27^ | nr | nr | 17 (3.9) | 413 (95.8) | nr | nr | 87 (20.2) |
| M. Kim, 2024 ^28^ | 72 (54.1) | 55 (41.3) | nr | nr | 6 (4.5) | LGD | nr |
| R. Kim, 2024 ^29^ | 481 (100) | 0 (0) | 0 (0) | 0 (0) | 0 (0) | HGD | 38 (7.9) |
| Kimura, 2017 ^30^ | 28 (39.4) | 36 (50.7) | 2 (2.8) | 0 (0) | 5 (7) | HGD | nr |
| Li, 2020 ^32^ | 73 (58.4) | 32 (25.6) | 6 (4.8) | 4 (3.2) | 10 (8) | nr | 14 (11.2) |
| Lubezky, 2010 ^33^ | nr | nr | nr | 0 (0) | 0 (0) | LGD | 8 (20.5) |
| Majumder, 2019 ^35^ | nr | nr | nr | 0 (0) | 0 (0) | HGD | nr |
| Marchegiani, 2015 ^37^ | 204 (64.5) | 69 (21.8) | 9 (2.8) | nr | 34 (10.7) | nr | 55 (17.4) |
| Miller, 2011 ^39^ | 117 (61) | 57 (30) | nr | 10 (5) | 7 (4) | HGD | 38 (19.9) |
| Nagai, 2008 ^40^ | nr | nr | nr | nr | nr | HGD | nr |
| Nakagohri, 2002 ^41^ | 6 (37.5) | 9 (56.2) | 0 (0) | 0 (0) | 1 (6.2) | nr | 0 (0) |
| Nakagohri, 2007 ^42^ | nr | nr | nr | nr | nr | nr | 2 (14.3) |
| Nakagohri, 2010 ^43^ | 1 (7.7) | nr | nr | nr | 12 (92.3) | HGD | 3 (23.1) |
| Niedergethmann, 2008 ^44^ | nr | nr | nr | nr | nr | nr | (0) |
| Park, 2011 ^45^ | nr | nr | nr | 0 (0) | 0 (0) | nr | (0) |
| Pea, 2017 ^47^ | 184 (71) | 73 (28) | 0 (0) | nr | 3 (1) | HGD | 42 (16.2) |
| Pfluger, 2022 ^48^ | 90 (71.4) | 33 (26.2) | 0 (0) | 0 (0) | 3 (2.4) | HGD | 90 (71.4) |
| Ridtitid, 2016 ^49^ | 63 (53.8) | 46 (39.3) | 4 (3.4) | 4 (3.4) | nr | HGD | 6 (5.1) |
| Salvia 2004 ^50^ | nr | nr | nr | nr | nr | LGD | 0 (0) |
| Sauvanet, 2014 ^51^ | 0 (0) | 0 (0) | 0 (0) | 47 (61) | 30 (3.9) | nr | nr |
| Schnelldorfer, 2008 ^52^ | nr | nr | nr | nr | nr | HGD | nr |
| Stauffer, 2009 ^53^ | 0 (0) | 0 (0) | 21 (100) | 0 (0) | nr | HGD | 0 (0) |
| Sugimachi, 2021 ^54^ | 21 (60) | 11 (31.4) | 3 (8.5) | 0 (0) | nr | HGD | nr |
| Sugiyama, 2003 ^55^ | nr | nr | nr | nr | nr | HGD | 2 (10.5) |
| Takahashi, 2006 ^56^ | 13 (65) | 5 (25) | 1 (5) | nr | 1 (5) | In situ Carcinoma | 0 (0) |
| Tamura, 2014 ^57^ | 17 (47.2) | 16 (44.4) | 3 (5.5) | nr | nr | HGD | nr |
| Thomas, 2015 ^58^ | 5 (29.4) | 0 (0) | 0 (0) | 12 (70.5) | nr | nr | nr |
| Wada, 2005 ^59^ | nr | nr | nr | nr | nr | HGD | 1 (1.9) |
| Winner, 2013 ^60^ | nr | nr | nr | nr | nr | HGD | nr |
| Xourafas, 2015 ^62^ | 67 (77) | 12 (13.8) | 1 (1.1) | 0 (0) | 7 (11.5) | HGD | 17 (19.5) |
| Yamaguchi, 2016 ^63^ | nr | nr | nr | nr | nr | HGD | 11 (24.4) |
| Yogi, 2015 ^65^ | nr | nr | nr | nr | nr | HGD | nr |
| Yokoyama, 2007 ^66^ | nr | nr | nr | nr | nr | HGD | nr |

Supplementary Table 8: Demographics and morphology of patients undergoing resection for IPMN-derived PC (n= number of patients, % = percentage, HGD = high grade dysplasia, y = year, MD-IPMN = main duct IPMN, BD-IPMN = branch duct IPMN, MT-IPMN = mixed type IPMN, IQR = interquartile range, cm = centimeter, nr = not reported)

| Study | Sample-size, n | Malignant, n (%) | | | HGD  n (%) | Sex, n (%) | | Median age, y (IQR) | IPMN location, n (%) | | | Morphological subtype, n (%) | | | | IPMN subtype, n (%) | | | | IPMN cyst size, cm | Tumor size, cm | Multi-  focality, n (%) |
| --- | --- | --- | --- | --- | --- | --- | --- | --- | --- | --- | --- | --- | --- | --- | --- | --- | --- | --- | --- | --- | --- | --- |
|  |  | IPMN derived PC | Micro-invasive | nr |  | M | F |  | Head/neck | Body/tail | other | MD-IPMN | BD-IPMN | MT- IPMN | NR | Colloid | Onco-cytic | Tubular | other |  |  |  |
| Abdalla, 2024 ^1^ | 217 | 217 (100) | 0 (0) | 0 (0) | 0 (0) | 116 (53.5) | 101 (46.5) | Mean 71 ± 13 | nr | nr | nr | nr | nr | nr | 217 (100) | nr | nr | nr | nr | nr | nr | nr |
| Chari, 2002 ^8^ | 40 | 40 (100) | 0 (0) | 0 (0) | 0 (0) | 25 | 15 | 67 (2) | nr | nr | nr | Nr | nr | nr | 40 (100) | 0 | 0 | 27 (67.5) | 13 (32.5) | nr | nr | nr |
| Chunhui, 2014 ^9^ | 12 | 12 (100) | 0 (0) | 0 (0) | 0 (0) | nr | nr | nr | nr | nr | nr | 7 (58.3) | 2 (16.7) | 3 (25) | 0 (0) | nr | nr | nr | nr | nr | nr | nr |
| Crippa, 2010 ^10^ | 118 | nr | nr | 118 (100) | 0 (0) | nr | nr | nr | nr | nr | nr | 39 | 17 | 62 | 0 (0) | nr | nr | nr | nr | nr | nr | nr |
| Cuillerier, 2000 ^11^ | 19 | 19 (100) | 0 (0) | 0 (0) | 0 (0) | nr | nr | nr | nr | nr | nr | nr | nr | nr | 19 (100) | nr | nr | nr | 19 (100) | nr | nr | nr |
| Fuji, 2022 ^16^ | 30 | 30 (100) | 0 (0) | 0 (0) | 0 (0) | nr | nr | nr | nr | nr | nr | Nr | nr | nr | 30 (100) | nr | nr | nr | 30 | nr | nr | nr |
| Habib, 2022 ^17^ | 213 | 213 (100) | 0 (0) | 0 (0) | 0 (0) | 122 (57.3 | 91 (42.7) | Mean 69.9 ± 10.1 | nr | nr | nr | 59 (27.7) | 62 (29.1) | 87 (40.8) | 5 (2.3) | 45 (21.1) | 0 (0) | 168 (78.9) | 0 (0) | nr | nr | nr |
| Hirono, 2016 ^18^ | 85 | 85 (100) | 0 (0) | 0 (0) | 0 (0) | nr | nr | nr | nr | nr | nr | nr | nr | nr | 85 (100) | 24 (28.2)) | 0 | 61 71.8) | 0 | nr | nr | nr |
| Hirono, 2020 ^19^ | 247 | 247 (100) | 0 (0) | 0 (0) | 0 (0) | nr | nr | nr | nr | nr | nr | Nr | nr | nr | 247 (100) | 71 (28.7) | 0 | 126 (51.0) | 50 (20.2) | nr | nr | nr |
| Hirono, 2024 ^20^ | 1143 | 1143 (100) | 0 (0) | 0 (0) | 0 (0) | nr | nr | nr | nr | nr | nr | 265 (23.2) | 227 (19.9) | 651 (57) | 0 (0) | 357 (33.9) | 0 | 697 (66.1) | 89 (7.8) | nr | nr | nr |
| Jang, 2016 ^21^ | 11 | 11 (100) | 0 (0) | 0 (0) | 0 (0) | nr | nr | nr | nr | nr | nr | 3 (27.3) | 0 | 8 (72.7) | 0 (0) | nr | nr | nr | nr | nr | nr | nr |
| Kaiser, 2023 ^23^ | 19 | 19 (100) | 0 (0) | 0 (0) | 0 (0) | 11 (57.9) | 8 (42.1) | 46.0 (44.0-48.5) | 10 (52.6) | 7 (36.9) | 2 diffuse (10.5) | 0 (0) | 1 (5.3) | 16 (84.2) | 2 (10.5) | nr | nr | nr | 19 | Median 4.5 (IQR 3.1-5.5 | nr | 2 (10.5) |
| Kamata, 2018 ^24^ | 42 | 42 (100) | 0 (0) | 0 (0) | 0 (0) | nr | nr | nr | nr | nr | nr | nr | nr | nr | 42 (100) | nr | nr | nr | nr | nr | nr | nr |
| Kang, 2014 ^25^ | 68 | 68 (100) | 0 (0) | 0 (0) | 0 (0) | nr | nr | nr | nr | nr | nr | nr | nr | nr | 68 (100) | 25 (36.8) | 5 (7.4) | 26 (38.2) | 12 (17.6) | nr | nr | nr |
| S. Kim, 2008 ^26^ | 28 | 28 (100) | 0 (0) | 0 (0) | 0 (0) | 20 | 8 | mean 63.8 ± 7.7 | nr | nr | nr | 25 (89.3) | 3 (10.7) | 0 | 0 (0) | nr | nr | nr | nr | Mean 1.6 (SD ±1.0) | Mean 4.9 (SD ±3.8) | nr |
| H. Kim, 2022 ^27^ | 117 | 117 (100) | 0 (0) | 0 (0) | 0 (0) | nr | nr | nr | 72 (61.5) | 33 (28.2) | 12 (10.3) diffuse | 25 (21.4) | 43 (36.8) | 49 (41.9) | 0 (0) | nr | nr | nr | nr | nr | nr | 9 (7.7) |
| M. Kim, 2024 ^28^ | 118 | 69 (58.5) | 0 (0) | 0 (0) | 49 (41.5) | 64 (54.2) | 54 (45.8) | mean 72.1 (41-91) | 74 (62.7) | 40 (33.9) | 4 diffuse (3.4) | 15 (12.7) | 100 (84.4) | 3 (2.5) | 0 (0) | nr | nr | nr | nr | Mean 3.0 ± 1.7) | nr | 24 (20.3) |
| Kimura, 2017 ^30^ | 27 | 27 (100) | 0 (0) | 0 (0) | 0 (0) | 16 (59.3) | 11 (40.7) | 71.85 ± 7.26 | 17 (63.0) | 9 (33.3) | 1 diffuse (3.7) | 13 (48.1) | 11 (40.7) | 3 (11.1) | 0 (0) | 9 (33.3) | 3 (11.1) | 15 (55.6) | 0 | nr | nr | 1 (3.7) |
| Kurahara, 2015 ^31^ | 11 | 5 (45.5) | 6 (54.5) | 0 (0) | 0 (0) | nr | nr | nr | nr | nr | nr | nr | nr | nr | 11 (100) | nr | nr | nr | nr | nr | nr | nr |
| Li, 2020 ^32^ | 93 | 0 (0) | 0 (0) | 93 (100) | 0 (0) | 61 | 32 (34.4) | 64 (56.5-71) | nr | nr | nr | 21 (22.6) | 13 (20.4) | 59 (63.4) | 0 (0) | 34 (36.6) | 7 7.5) | 18 19.4) | 34 (36.6) | nr | Median 40 (IQR 27.5-67.5) | nr |
| Lucocq, 2024 ^34^ | 415 | 415 (100) | 0 (0) | 0 (0) | 0 (0) | 227 (55) | 188 (45) | nr | 261 (62.9) | 111 (27) | 41 (9.9) diffuse  2 nr | 238 (57.3) | 61 (14.7) | 92 (22) | 24 (5.8) | 64 (15.4) | 20 (4.8) | 331 (79.8) | 0 (0) | nr | nr | nr |
| Marchegiani, 2015  ^36^  Oncocytic | 11 | 0 (0) | 0 (0) | 11 (100) | 0 (0) | nr | nr | nr | nr | nr | nr | nr | nr | nr | 11 (100) | nr | 11 (100) | nr | nr | nr | nr | 0 (0) |
| Marchegiani, 2015  ^37^  Patterns | 84 | 84 (100) | 0 (0) | 0 (0) | 0 (0) | 54 | 30 | 69.5 (47-92) | nr | nr | nr | nr | nr | nr | 84 (100) | 36 (42.9) | 12 (14.3) | 9 (10.7) | 26 gastric  1  mixed | nr | nr | nr |
| Marchegiani, 2019 ^38^ | 102 | 102 (100) | 0 (0) | 0 (0) | 0 (0) | 57 (55.9) | 45 (44.1) | 66 (24-84) | nr | nr | nr | 21 (20.6) | 11 (10.8) | 70 (68.6) | 0 (0) | 50 (48.9) | 2 (2.1) | 50 (48.9) | 0 | Median 2.3 (IQR 0.2-9.3) | nr | nr |
| Nagai, 2008 ^40^ | 30 | 30 (100) | 0 (0) | 0 (0) | 0 (0) | nr | nr | nr | nr | nr | nr | 10 (33.3) | 18 (60) | 2 (6.7) | 0 (0) | Nr | nr | nr | nr | nr | nr | nr |
| Nakagohri, 2007 ^42^ | 37 | 31 (84) | 6 (16) | 0 (0) | 0 (0) | nr | nr | nr | nr | nr | nr | 13 (35.1) | 24 (64.9) | 0 (0) | 0 (0) | Nr | nr | nr | nr | nr | nr | nr |
| Niedergethmann, 2008 ^44^ | 68 | nr | nr | 68 (100) | nr | nr | nr | nr | nr | nr | nr | nr | nr | nr | 68 (100) | Nr | nr | nr | nr | nr | Median 4 (IQR 0.4-15) | nr |
| Park, 2011 ^45^ | 35 | 35 (100) | 0 (0) | 0 (0) | 0 (0) | nr | nr | nr | nr | nr | nr | nr | nr | nr | 35 (100) | Nr | nr | nr | nr | nr | nr | nr |
| Partelli, 2010 ^46^ | 104 | 104 (100) | 0 (0) | 0 (0) | 0 (0) | 55 (21.9) | 49 (47.1) | 69 (61-74) | 69 (66.3) | 16 (15.4) | 19 whole gland (18.3) | 88 (84.6) | 16 (15.4) | 0 | 0 (0) | Nr | nr | nr | nr | Median 4.0 (2.5-6.0) | nr | nr |
| Salvia, 2004 ^50^ | 58 | 58 (100) | 0 (0) | 0 (0) | 0 (0) | nr | nr | 67.3 (58-72) | nr | nr | nr | 58 (100) | 0 | 0 | 0 (0) | Nr | nr | nr | nr | nr | nr | 30 (51.7) |
| Schnelldorfer, 2008 ^52^ | 63 | 63 (100) | 0 (0) | 0 (0) | 0 (0) | nr | nr | nr | nr | nr | nr | nr | nr | nr | 63 (100) | nr | nr | nr | nr | Mean 4.9 (SD ±0.6) | Mean 2.9 (SD ±0.3) | 3 (5%) |
| Sugimachi, 2021 ^54^ | 15 | 15 (100) | 0 (0) | 0 (0) | 0 (0) | 5 (33.3) | 10 (66.6) | 72.7 ±2.1 mean | 6 (40) | 7 (46.7) | 2 diffuse (13.3) | 4 (26.7) | 7 (46.7) | 4 (26.7) | 0 (0) | nr | nr | nr | nr | nr | nr | nr |
| Tamura, 2014 ^57^ | 19 | 12 (63.2) | 7 (36.8) | 0 (0) | 0 (0) | 11 (57.9) | 8 (42.1) | 69.0 ± 6.8 mean | 10 (52.6) | 7 (36.8) | 2 diffuse (10.5) | 19 (100) | 0 | 0 | 0 (0) | 8 (42.1) | 1 (5.3) | 9 (47.4) | 1 | nr | nr | nr |
| Wada, 2005 ^59^ | 25 | 25 (100) | 0 (0) | 0 (0) | 0 (0) | 14 (56) | 11 (44) | mean 65.0 ±2.5 | nr | nr | nr | nr | nr | nr | 25 (100) | nr | nr | nr | nr | nr | nr | nr |
| Winner, 2013 ^60^ | 28 | 28 (100) | 0 (0) | 0 (0) | 0 (0) | nr | nr | nr | nr | nr | nr | nr | nr | nr | 28 (100) | nr | nr | nr | nr | nr | nr | nr |
| Woo, 2008 ^61^ | 19 | 19 (100) | 0 (0) | 0 (0) | 0 (0) | 13 | 6 | mean 62.1 ±10 | nr | nr | nr | nr | nr | nr | 19 (100) | nr | nr | nr | nr | Mean 4.6 (SD±3.3) | | nr |
| Yamashige, 2025 ^64^ | 136 | 136 (100) | 0 (0) | 0 (0) | 0 (0) | nr | nr | 72 (64-77) | nr | nr | nr | nr | nr | nr | 136 (100) | 49 (36.0) | nr | 36 (26.5) | 51 gastric (37.5) | nr | nr | nr |
| Yogi, 2015 ^65^ | 28 | 28 (100) | 0 (0) | 0 (0) | 0 (0) | nr | nr | ns | nr | nr | nr | nr | nr | nr | 28 (100) | nr | nr | nr | nr | nr | nr | nr |
| Yokoyama, 2007 ^66^ | 15 | 15 (100) | 0 (0) | 0 (0) | 0 (0) | nr | nr | nr | nr | nr | nr | nr | nr | nr | 15 (100) | nr | nr | nr | nr | nr | nr | nr |

Supplementary Table 9: Surgical characteristics and final histopathology of patients undergoing resection for IPMN-derived PC (n = number of patients, % = percentage, PD = pancreatoduodenectomy, LP = left pancreatectomy, TP = total pancreatectomy, CP = central pancreatectomy, Margin+ = positive surgical resection margin (≥LGD), LVI = lymphovascular invasion, PNI = perineural invasion, nr = not reported)

| Study | Type of resection, n (%) | | | | Adjuvant therapy, n (%) | LNR (lymph nodes resected ), n (%) | N-stage, N+ n (%) | Margin+, n (%) | LVI, n(%) | PNI, n (%) |
| --- | --- | --- | --- | --- | --- | --- | --- | --- | --- | --- |
|  | PD | LP | TP | Other |  |  |  |  |  |  |
| Abdalla, 2024 ^1^ | 133 (61.3) | 27 (12.4) | 44 (20.3) | 13 (5.9) NR | 74 (34.1) | nr | 90 (41) | 27 (12) | nr | nr |
| Chari, 2002 ^8^ | 19 (47.5) | 8 (20.0) | 13 (32.5) | 0 | nr | nr | 12 (30.0) | 7 (17.5) | nr | 9 (22.5) |
| Chunhui, 2014 ^9^ | nr | nr | nr | nr | nr | nr | 9 (75.0) | nr | 3 (25.0) | 11 (91.7) |
| Crippa, 2010 ^10^ | nr | nr | nr | nr | nr | nr | 47 (39.8) | nr | nr | nr |
| Cuillerier, 2000 ^11^ | 0 | 0 | 4 (21.1) | 15 partial (78.9) | nr | nr | 8 (42.1) | 8 (42.1) | nr | nr |
| Fuji, 2022 ^16^ | nr | nr | nr | nr | 20 (66.7) | nr | 12 (40.0) | 3 (10.0) | nr | nr |
| Habib, 2022 ^17^ | 133 (62.4) | 39 (18.3) | 41 (19.2) | nr | 93 (43.7) | nr | 54 (25.4) N1, 49 (23.0) N2 | 40 (18.8) | 66 (31) | 120 (56.3) |
| Hirono, 2016 ^18^ | nr | nr | nr | nr | 45 (52.9) | nr | 33 (38.8) | 0 | nr | nr |
| Hirono, 2020 ^19^ | nr | nr | nr | nr | 88 (35.6) | nr | 76 (30.8) | nr | nr | nr |
| Hirono, 2024 ^20^ | 655 (57.3) | 300 (26.2) | 172 (15.0) | 16 (1.4) CP | 819 chemo; 333 completed (29.1), 486 AT after surgery (42.5) | nr | 336 (29.4) | nr | 511 (44.7) | 436 (38.1) |
| Jang, 2016 ^21^ | nr | nr | nr | nr | nr | nr | nr | nr | nr | nr |
| Kaiser, 2023 ^23^ | 8 (50) | 6 (31.6) | 5 (26.3) | 0 | 19 (100) recommended, nr completed | nr | 13 (68.4) | 8 (42.1) | nr | nr |
| Kamata, 2018 ^24^ | nr | nr | nr | nr | nr | nr | nr | nr | nr | nr |
| Kang, 2014 ^25^ | nr | nr | nr | nr | 68 (100) | nr | nr | nr | nr | nr |
| S. Kim, 2008 ^26^ | nr | nr | nr | nr | nr | nr | 8 (28.6) | 3 (10.7) | nr | nr |
| H. Kim, 2022 ^27^ | nr | nr | 18 (15.4) | 99 (84.6) | 56 (47.9) chemo , 45 (42.1) radio;     all 117 indicated to have adjuvant  chemo | nr | 32 (27.6) | 22 (18.8) | 23 (20.2) | 50 (43.9) |
| M. Kim, 2024 ^28^ | 75 (63.6) | 39 (33.1) | 0 | 4 (3.4) CP | nr | 15 (11.3) | 6 (4.5) | nr | nr | nr |
| Kimura, 2017 ^30^ | 15 (55.6) | 9 (33.3) | 3 (11.1) | 0 | nr | nr | 7 (25.9) | nr | nr | nr |
| Kurahara, 2015 ^31^ | nr | nr | nr | nr | nr | nr | nr | nr | nr | nr |
| Li, 2020 ^32^ | 67 (72.0) | 10 (10.8) | 9 (9.7) | 7 (7.5) CP | nr | nr | 14 (15.1) | 29 (31.2) | 18 (19.4) | 28 (30.1) |
| Lucocq, 2024 ^34^ | 234 (56) | 94 (23) | 87 (21) | 0 (0) | 250 (60.2) | nr | 193 (46.5) | 153 (36.9) | 216 (52) | 230 (55) |
| Marchegiani, 2015 ^36^ oncocytic | nr | nr | nr | nr | nr | nr | 2 (18) | nr | nr | nr |
| Marchegiani, 2015 ^37^ patterns | 47 (55.9%) | 23 (27.4%) | 13 (15.5%) | 1 (1.2) CP | 18 (21.4) chemo, 17 (20.2) chemo +radio | nr | nr | nr | nr | nr |
| Marchegiani, 2019 ^38^ | 59 (57.9) | 23 (22.5) | 20 (19.6) | 0 (0) | 19 (18.6) chemo, 5 of them + radio | nr | 44 (43.1) | 12 (11.8) | nr | nr |
| Nagai, 2008 ^40^ | nr | nr | nr | nr | nr | nr | nr | 0 | nr | nr |
| Nakagohri, 2007 ^42^ | nr | nr | nr | nr | nr | nr | 15 (40.5) | 17 (45.9)) | 16 (43.2) | 16 (43.2) |
| Niedergethmann, 2008 ^44^ | nr | nr | nr | nr | nr | nr | 28 (41.2) | 8 (11.8) | 21 (30.9) | 20 (29.4) |
| Park, 2011 ^45^ | nr | nr | nr | nr | nr | nr | 7 (20) | nr | nr | nr |
| Partelli, 2010 ^46^ | 69 (66.3) | 14 (13.5) | 19 (8.3) | 2 (1.9) CP | 39 (37.5) chemo and/or radio | 0 = 60 (57.7%), 0 < LNR ≤ 0.2 26(25%), >0.2 18 (17.3%)  (15 (9; 23)) | 44 (42.3) | 13 (12.5) | 25 (24.0) | 42 (40.4) |
| Salvia, 2004 ^50^ | nr | nr | nr | nr | nr | nr | 24 (41.4) | 6 (10.3) | nr | nr |
| Schnelldorfer, 2008 ^52^ | nr | nr | nr | nr | 28 (44.4) chemo, 23 (36.5) radio | nr | 24 (38.1) | nr | nr | 35 (55.5) |
| Sugimachi, 2021 ^54^ | 6 (40.0) | 6 (40.0) | 3 (20.0) | 0 | nr | nr | nr | nr | nr | nr |
| Tamura, 2014 ^57^ | 9 (47.4) | 6 (31.6) | 4 (21.1) | 0 | nr | nr | nr | nr | nr | nr |
| Wada, 2005 ^59^ | nr | nr | nr | nr | nr | nr | 8 (32.0) | nr | 2 (8.0) | 11 (44.0) |
| Winner, 2013 ^60^ | nr | nr | nr | nr | 12 (42.9) chemo | (15 (IQR 9–25), 4.3 (28.6 %)) | 8 (28.6) | nr | 8 (28.6) | 8 (28.6) |
| Woo, 2008 ^61^ | nr | nr | nr | nr | nr | nr | 1 (5.2) | nr | 1 (5.2) | 2 (10.5) |
| Yamashige, 2025 ^64^ | nr | nr | nr | nr | nr | nr | nr | nr | nr | nr |
| Yogi, 2015 ^65^ | nr | nr | nr | nr | nr | nr | nr | nr | nr | nr |
| Yokoyama, 2007 ^66^ | nr | nr | nr | nr | nr | nr | nr | nr | nr | nr |

Supplementary Table 10: Follow-up of patients undergoing resection for IPMN-derived PC (n = number, % = percentage, IQR = interquartile range, nr = not reported, y=years, m=months)

| Study | Median follow-up time, months (IQR) | Follow up sample size, n (%) | Overall recurrence, n (%) | Median time to recurrence, months (IQR) | Recurrence location, n (%) | | | | Recurrence type, n (%) | | | | | Secondary therapy, n (%) | Final histopathology, n (%) | Overall survival | Disease free survival |
| --- | --- | --- | --- | --- | --- | --- | --- | --- | --- | --- | --- | --- | --- | --- | --- | --- | --- |
|  |  |  |  |  | Systematic | Loco-regional | Extra-pancreatic not further specified | nr | Non-invasive | IPMN-derived PC | Meta-chronous PDAC | Metastatic | nr |  |  |  |  |
| Abdalla, 2024 ^1^ | 200 | 217 (100) | 78 (35.9) | 31 ±18 | 54 (24.9) | 24 (11.1 | 0 (0) | 0 (0) | nr | nr | nr | nr | 78 (35.9) | nr | nr | 1 y 78%, 3 y 43%, 5 y 33% | Mean 31 m (SD ± 18.0) |
| Chari, 2002 ^8^ | 42 | 40 (100) | 26 (65.0) | 18 ±3 | 17 (42.5) | 6 (15.0) | 0 | 3 (7.5) | nr | nr | nr | nr | 26 (65.0) | 18 (45.0) chemo  4 (10.0) surgery | nr | 5 y 36% | nr |
| Chunhui, 2014 ^9^ | 42 | 12 (100) | 0 | - | 0 | 0 | 0 | 0 | 0 | 0 | 0 | nr | 0 | nr | - | 3.5 y 100% | 3.5 y  100% |
| Crippa, 2010 ^10^ | nr | 114 (97) | 49 (43.0) | MD 20.6 (IQR:4-50), Mixed 18.5 (IQR:4-67), BD 28 (IQR 6-72) | nr | nr | 0 | 49 (43.0) | nr | nr | nr | nr | 49 (43.0) | nr | nr | nr | nr |
| Cuillerier, 2000 ^11^ | nr | 19 (100) | 13 (68.4) | nr | nr | nr | nr | 13 (68.4) | 0 | 13 (68.4) | nr | nr | 0 | nr | nr | 1 y 60.0%,  5 y 40% | nr |
| Fuji, 2022 ^16^ | nr | 30 (100) | 18 (60.0) | nr | 7 (23.3) | 11 (36.7) | nr | 0 | nr | nr | nr | nr | 18 (60.0) | 9  (30.0) surgery | nr | nr | nr |
| Habib, 2022 ^17^ | 36.6 (15.7-72.4) | 213 (100) | 92 (43.2) | local 21.6 (10.2-33.0)  systemic 11.4 (7.8-15.1) | 60 (28.2) | 32 (15.0) | nr | 0 | nr | nr | nr | nr | 92 (43.2) | nr | nr | 1 y 84.7%, 3 y 58.0%, 5 y 44.6% , | 48.3 months (36.9–59.8) |
| Hirono, 2016 ^18^ | nr | 85 (100) | 46 (54.1) | nr | 0 (0) | 6 (13.0) | 40 (47.1) | 0 | nr | nr | nr | nr | 46 (54.1) | 1 (1.2) chemo, 1 (1.2) palliative, 4 (4.7) surgery | 1 (1.2) HGD, 2 (2.4)  IC, 1 (1.2) PDAC | nr | nr |
| Hirono, 2020 ^19^ | nr | 247 (100) | 107 (43.3) | 71.4 | 3 (1.2) | 25 (10.1) | 79 (32.0) | 0 | nr | nr | nr | nr | 107 (43.3) | nr | nr | nr | 71.4 months |
| Hirono, 2024 ^20^ | 46.8 (4.9-236.2) | 1143 (100) | 484 (40.9) | 14.2 | 0 (0) | 94 (7.9) | 390 (33.0) | 0 | nr | nr | 27 (2.4) | 286 (25.0) | 171 (35.3) | 93 (8.1) surgery  299 (26.2) chemo  21 (1.8) radiation | nr | 5 y 63.7%, 10 y 49.2% | 5 y 57.8%, 10 y 52.1 % |
| Jang, 2016 ^21^ | nr | 11 (100) | 1 (9.1) | nr | nr | nr | 0 | 1 (9.1) | 0 | 1 (9.1) | nr | nr | 0 | 1 (9.1) palliative | nr | nr | nr |
| Kaiser, 2023 ^23^ | 26 (18.0-53.0) | 10 (52.6) | 6 (31.6) | nr | 4 (21.0) | 2 (10.5) | 0 | 0 | 0 | 6 (31.6) | nr | nr | 0 | nr | nr | 5 y 29% | nr |
| Kamata, 2018 ^24^ | nr | 41 (97.6) | 9 (22.0) | 12 (6-67) | 7 (17.1) | 2 (4.9) | 0 | 0 | 1 (2.4) | 7 (17.1) | 1 (2.4) | nr | 0 | nr | nr | nr | nr |
| Kang, 2014 ^25^ | nr | 68 (100) | 23 (33.8) | 18.1 (2.5-214.4) | nr | nr | 0 | 23 (33.8) | 0 | 0 | 2 (2.9) | nr | 21 (30.9) | 3 (4.4) surgery,1 (1.5) palliative | 2 (2.9) I-IPMN, 1 (1.5) PDAC | nr | 5 y 48.7% |
| S. Kim, 2008 ^26^ | nr | 28 (100) | 12 (42.9) | 30 (8-44) | 12 (42.9) | 0 | 0 | 0 | 0 | 12 (42.9) | nr | nr | 0 | nr | nr | 5 y 48.4 | 5 y 45.4 |
| H. Kim, 2022 ^27^ | 56.0 (0.4-223.0) | 117 (100) | 40 (34.2) | nr | 29 (72.5) | 11 (27.5) | 0 | 0 | nr | 40 (34.2) | nr | nr | nr | nr | nr | 5 y 48.4% | 5 y 45.5% |
| M. Kim, 2024 ^28^ | nr | 118 (100) | 42 (35.6) | nr | 0 | 42 (35.6) | 0 | 0 | 30 (10.2) | 12 (10.2) | nr | nr | 0 | nr | nr |  |  |
| Kimura, 2017 ^30^ | 55 (10-210) | 27 (100) | 13 (48.1) | nr | 0 | 0 | 0 | 13 (48.1) | nr | nr | nr | nr | 13 (48.1) | nr | nr | 5 y T1a 45%, T1b 42.7%, >T1c 25.4% | nr |
| Kurahara, 2015 ^31^ | nr | 11 (100) | 7 (63.6) | nr | 5 (45.5) | 2 (18.2) | 0 | 0 | 0 | 7 (63.6) | ns | nr | 0 | 2 (18.2) surgery | 2 (18.2) micro- invasive ipmn | 5 y 40% | nr |
| Li, 2020 ^32^ | 35 (19-63) | 93 (100) | 22 (23.7) | 23 (14-52) | nr | nr | 0 | 22 (23.7) | nr | nr | nr | nr | 22 (23.7) | nr | nr | 1 y 97.1%,  3 y 94.2%  5 y 91.3% | 1 y 75.3%, 3 y 74%,  5 y 71.4% |
| Lucocq, 2024 ^34^ | 78 | 415 (100) | 197 (47.5) | nr | 123 (36.9) | 74 (17.8) | 0 | 0 | nr | nr | nr | nr | 197 (47.5) | 95 (48.2) chemo  15 (7.6) radio  8 (4.1) surgery | nr | nr | nr |
| Marchegiani, 2015  ^36^  oncocytic | nr | 11 (100) | 6 (67) | 60.5 (36-135) | 0 | 2 (18.2) | 0 | 4 (36.4) | 1 (9.1) | 5 (45.5) | nr | nr | 0 | 3 (27.3) surgery  3 (27.3) conservative | 1 (9.1) benign  2 (18.2) I-IPMN | nr | nr |
| Marchegiani, 2015  ^37^  patterns | nr | 82 (97.6) | 37 (45.1) | 19 (1-169) | 15 (18.3) | 22 (26.8) | 0 | 0 | 1 (1.2) | 36 (43.9) | 0 | nr | 0 | 4 (4.9%) surgery | 3 (3.7)  I-IPMN, 1 (1.2) HGD | 5 y 49% | 5 y 61% |
| Marchegiani, 2019 ^38^ | 72 (5-318) | 102 (100) | 41 (40.2) | nr | 34 (33.3) | 7 (6.9) | 0 | 0 | nr | nr | nr | nr | 41 (40.2) | nr | nr | 5 y 65.3% | nr |
| Nagai, 2008 ^40^ | nr | 30 (100) | 12 (40.0) | nr | 4 (13.3) | 7 (23.3) | 0 | 1 (3.3) | nr | nr | nr | nr | 12 (40.0) | nr | nr | 5 y 57.6%, 10 y 43.2% | nr |
| Nakagohri, 2007 ^42^ | nr | 37 (100) | 17 (45.9) | nr | 0 | 0 | 0 | 17 (45.9) ns | nr | nr | nr | nr | 17 (45.9 | nr | nr | 5 y 40% | nr |
| Niedergethmann, 2008 ^44^ | 36 (100) | 68 (100) | 19 (27.9) | nr | 0 | 2 (2.9) | 0 | 17 (25) | nr | nr | nr | nr | 19 (27.9) | 17 (89.5) palliative  2 (2.9) surgery | 2 (2.9) invasive IPMN | 10 y 25% | nr |
| Park, 2011 ^45^ | 38.4 mean | 33 (94.3) | 12 (36.4) | nr | 7 (21.2) | 5 (15.2) | 0 | 0 | nr | nr |  | nr | 12 (36.4) | nr | nr | nr | nr |
| Partelli, 2010 ^46^ | 44 (17.7-96.9) | 104 (100) | 49 (47.1) | nr | 39 (37.5) | 10 (9.6) | 0 | 0 | nr | nr |  | nr | 49 (47.1) | nr | nr | 5 y 54.5% | nr |
| Salvia, 2004 ^50^ | 31 | 57 (98.3) | 7 (12.3) | nr | 2 (3.5) | 5 (8.8) | 0 | 0 | nr | nr | nr | nr | 7 (12.3) | 5 (8.8) surgery,  2 (3.5) palliative | nr | 5 y 60%, 10 y 50% | nr |
| Schnelldorfer, 2008 ^52^ | nr | 57 (90.5) | 33 (57.9) | nr | 26 (45.6) | 7 (12.3) | 0 | 0 | nr | nr | nr | nr | 33 (57.9) | 2  (3.5) surgery  2 (3.5) radiotherapy  16 (28.1) chemo | nr | 5 y 31% | 5 y 66% |
| Sugimachi, 2021 ^54^ | nr | 15 (100) | 8 (53.3) | nr | nr | nr | 0 | 8 (53.3) | nr | nr | nr | nr | 8 (53.3) | nr | nr | nr | 3 y 20.8% |
| Tamura, 2014 ^57^ | nr | 19 (100) | 8 (42.1) | nr | 6 (31.6) | 2 (10.5) | 0 | 0 | 1 (5.3) | 7 (36.8) | 0 | nr | 0 | 2 (3.5) surgery | 1 (5.3)  HGD  1 (5.3) Invasive | nr | nr |
| Wada, 2005 ^59^ | 27 ± 6 mean | 25 (100) | 12 (48.0) | mean 26±10 | nr | 1 (4.0) | 0 | 11 (44.0) | 0 | 12 (48.0) | nr | nr | 0 | 1 (2.1) surgery  11 (44.0) palliative | invasive cancer | 5 y 46% | nr |
| Winner, 2013 ^60^ | nr | 28 (100) | 9 (32.1) | 21.9 (12.1-46.1) | 3 (10.7) | 6 (21.4) | 0 | 0 | 1 (3.6) | 8 (28.6) | nr | nr | 0 | 6 (21.4) palliative  2 (7.1) surgery  1 (3.6) surveillance | 6 (21.4) PDAC  2 (7.1) I-IPMN  1 (3.6) benign IPMN | nr | 1 y 70%,  3 y 45% |
| Woo, 2008 ^61^ | 49 | 19 (100) | 3 (15.8) | 18.4 | 2 (10.6) | 1 (5.3) | 0 | 0 | nr | nr | nr | nr | 3 (15.8) | nr | nr | 5 y 34% | nr |
| Yamashige, 2025 ^64^ | 48.2 (29.8-80.5) | 136 (100)  for extrapancreatic, 119 (87.5) for remnant | 71 (52.2) | nr | nr | 22 (16.2) | 49 (36.0) | 0 | nr | nr | 6 (5.0) | nr | 65 (47.8) | nr | nr | nr | nr |
| Yogi, 2015 ^65^ | nr | 28 (100) | 16 (57.1) | nr | 16 (57.1) | 0 | 0 | 0 | 0 | 16 (57.1) | 0 | nr | - | nr | nr | nr | nr |
| Yokoyama, 2007 ^66^ | 30 (3-152) | 15 (100) | 1 (6.7) | 23 (13-23) | 1 (6.7) | nr | 0 | 0 | 0 | 1 (6.7) | 0 | nr | 0 | 1 (6.7) surgery | 1  6.7) invasive carcinoma | 5 y 66%, 10 y 36% | nr |

Supplementary Table 11: Overview of diagnostic tools used to evaluate recurrent disease in IPMN-derived PC (n=number of patients)

| Study | Radiologic follow-up only, n | Radiologic follow up and histologic confirmation, n |
| --- | --- | --- |
| Abdalla, 2024 ^1^ |  | 217 |
| Chari, 2002 ^8^ |  | 40 |
| Chunhui, 2014 ^9^ | 12 |  |
| Crippa, 2010 ^10^ | 114 |  |
| Cuillerier, 2000 ^11^ |  | 19 |
| Fuji, 2022 ^16^ |  | 30 |
| Habib, 2022 ^17^ | 213 |  |
| Hirono, 2016 ^18^ |  | 85 |
| Hirono, 2020 ^19^ |  | 247 |
| Hirono, 2024 ^20^ |  | 1143 |
| Jang, 2016 ^21^ | 11 |  |
| Kaiser, 2023 ^23^ | 10 |  |
| Kamata, 2018 ^24^ | 41 |  |
| Kang, 2014 ^25^ |  | 68 |
| S. Kim, 2008 ^26^ | 28 |  |
| H. Kim, 2022 ^27^ |  | 117 |
| M. Kim, 2024 ^28^ | 118 |  |
| Kimura, 2017 ^30^ | 27 |  |
| Kurahara, 2015 ^31^ |  | 11 |
| Li, 2020 ^32^ |  | 93 |
| Lucocq, 2024 ^34^ |  | 415 |
| Marchegiani, 2015 ^36^ (Oncocytic) |  | 11 |
| Marchegiani, 2015 ^37^ (patterns) | 82 |  |
| Marchegiani, 2019 ^38^ |  | 102 |
| Nagai, 2008 ^40^ | 30 |  |
| Nakagohri, 2007 ^42^ | 37 |  |
| Niedergethmann, 2008 ^44^ |  | 68 |
| Park, 2011 ^45^ |  | 33 |
| Partelli, 2010 ^46^ |  | 104 |
| Salvia, 2004 ^50^ | 57 |  |
| Schnelldorfer, 2008 ^52^ | 57 |  |
| Sugimachi, 2021 ^54^ | 15 |  |
| Tamura, 2014 ^57^ |  | 19 |
| Wada, 2005 ^59^ |  | 25 |
| Winner, 2013 ^60^ |  | 28 |
| Woo, 2008 ^61^ | 19 |  |
| Yamashige, 2025 ^64^ |  | 136 |
| Yogi, 2015 ^65^ |  | 28 |
| Yokoyama, 2007 ^66^ | 15 |  |
| **Total** | **886** | **3039** |

Supplementary Table 12: Predictors of recurrence following surgery for IPMN-derived PC (n = number of patients)

| **Invasive IPMN** | **Study** | **N** | **HR 95% CI** | **Outcome** |
| --- | --- | --- | --- | --- |
|  |  |  |  |  |
| 1. Tubular histological subtype | Hirono, 2024 ^20^ | 1143 | 1.56 (1.23-1.98) | RFS |
|  | Marchegiani, 2015 ^37^ | 84 | 2.7 (p=0.02) | RFS |
|  |  |  |  |  |
| 2. Lymph node metastases | Hirono, 2024 ^20^ | 1143 | 2.29 (1.82- 2.90) | RFS |
|  | H. Kim, 2022 ^27^ | 117 | 4.587 (1.980-10.630) | Cumulative risk of distant recurrence |

Supplementary Table 13: Follow-up of patients undergoing resection for non-invasive IPMN (n = number, % = percentage, IQR = interquartile range, nr = not reported, y=years)

| Study | Follow up sample size, n (%) | Median follow-up, months (IQR) | Total recurrence, n (%) | Overall recurrence, n (%) | | | Median time to recurrence, months (range) | Recurrence location, n (%) | | | Recurrence type, n (%) | | | Final histo-pathology, n (%) | Secondary therapy, n(%) | Overall survival rate |  |
| --- | --- | --- | --- | --- | --- | --- | --- | --- | --- | --- | --- | --- | --- | --- | --- | --- | --- |
|  |  |  |  | Progression persistent cyst, N (%) | De-Novo IPMN, N (%) | nr |  | Systemic | Loco-regional | nr | Non-invasive | Invasive | nr |  |  |  |  |
| Al Efishat, 2018 ^2^ | 319 (100) | 42 | 77 (24.1) | 10 (3.1) | 67 (21) | 0 (0) | 28 | 2 (0.6) | nr | 75 (23.5) | nr | 11 (3.4) | 66 (20.7) | 11 ipmn recurrence | 13 surgery  2 chemotherapy | nr |  |
| Aleotti, 2022 ^3^ | 118 (88.7) | 60 (14-123) | 13 (11.5) | nr | 13 (11.5) | 0 (0) | 44 (5-60) | 1 (0.8) | 12 (11) | 0 (0) | 12 (11) | 1 (0.8) | 0 (0) | 2; mixed ipmn LGD, 1 cyst LGD, 1 invasive +liver metastic, 9 BD-ipmn without WF/HRS | 13 surgery | nr |  |
| Amini, 2022 ^4^ | 449 (100) | 48.9 | 124 (27.6) | 73 (16.3) | 50 (11.1) | 1 (0.2) | nr | 9 (2) | 6 (1.3) | 109 (24.3) | 108 (24) | 16 (3.6) | 0 (0) | 7 PDAC | 7 surgery | 5y 87.2%, 10y  65.6% |  |
| Antoñanzas J, 2018 ^5^ | 18 (100) | 60 | 1 (5.5) | 1 (5.5) | 0 (0) | 0 (0) | 46 | nr | nr | 1 (5.5) | nr | nr | 1 (5.5) | nr | nr | nr |  |
| Blackham, 2017 ^6^ | 100 (100) | 35 (1-129) | 9 (9) | 2 (2) | 7 (7) | 0 (0) | 15 (7-72) | 2 (2) | 7 (7) | 0 (0) | 4 (4) | 5 (5) | 0 (0) | 1 progression into cholangiocarcinoma 1 recurrence malignancy remannt, 3 patients PDAC | 3 surgery  4 conservative  2 palliative | nr |  |
| Blair, 2022 ^7^ | 127 (100) | 68 (12-195) | 30 (23.6) | nr | nr | 30 (23.6) | nr | nr | nr | 30 (23.6) | nr | nr | 30 (23.6) | nr | 9 surgery  21 conservative | nr |  |
| Chari, 2002 ^8^ | 73 (100) | 36 | 5 (6.8) | nr | nr | 5 (6.8) | nr | 1 (1.3) | 4 (5.5) | 0 (0) | 2 (2.7) | 3 (4.1) | 0 (0) | nr | nr | 5Y 88% |  |
| Chunhui, 2014 ^9^ | 24 (100) | 42 | 0 (0) | nr | nr | 0 (0) | nr | nr | nr | 0 (0) | nr | nr | 0 (0) | nr | nr | 42 months 100% |  |
| Crippa, 2010 ^10^ | 263 (97) | 50 (2-233) | 10 (3.8) | nr | nr | 10 (3.8) | nr | nr | nr | 10 (3.8) | nr | nr | 10 (3.8) | nr | nr | nr |  |
| Cuillerier, 2000 ^11^ | 25 (96.2) | nr | 13 (52) | 2 (7.7) | nr | 11 (44) | nr | 10 (38) | 3 (11.5) | 0 (0) | nr | nr | 13 (52) | nr | nr | 1 y 100%, 5 y 100% |  |
| Dhar, 2018 ^12^ | 330 (100) | 36 | 34 (10.3) | 8 (2.4) | 26 (7.8) | 0 (0) | 22 | 10 (3) | 24 (7.2) | 0 (0) | 22 (6.7) | 12 (3.6) | 0 (0) | nr | nr | nr |  |
| Faitot, 2015 ^13^ | 38 (100) | 28 (8-73) | 2 (5.3) | nr | 2 (5.3) | 0 (0) | nr | nr | 2 (5.3) | 0 (0) | 2 (5.3) | 0 (0) | 0 (0) | nr | 2 surgery | nr |  |
| Frankel, 2013 ^14^ | 192 (100) | 46 | 40 (20.8) | 6 (3.1) | 32 (16.6) | 2 (1.0) | nr | nr | nr | 40 (20.8) | nr | 3 (1.6) | 37 (19.3) | 2 PDAC | 6 surgery | nr |  |
| Fuji, 2011 ^15^ | 84 (100) | 42.5 | 4 (4.8) | 0 (0) | 4 (4.8) | 0 (0) | nr | 0 (0) | 4 (4.8) | 0 (0) | 4 (4.8) | 0 (0) | 0 (0) | nr | nr | nr |  |
| Fuji, 2022 ^16^ | 61 (100) | 71 (37-103) | 8 (13.1) | nr | nr | 8 (13.1) | nr | 0 (0) | 8 (13.1) | 0 (0) | nr | nr | 8 (13.1) | nr | nr | nr |  |
| Hirono, 2016 ^18^ | 172 (100) | 53.5 (0.5-196) | 10 (5.8) | nr | nr | 10 (5.8) | 12.2 (2-72) | 2 (1.2) | 8 (4.7) | 0 (0) | nr | nr | 10 (5.8) | 2 HGD, 1 IGD, 1 PDAC | nr | nr |  |
| Hirono, 2020 ^19^ | 827 (100) | 0.2-241 | 48 (5.8) | 0 (0) | 48 (5.8) | 0 (0) | nr | 6 (0.7) | 42 (5.1) | 0 (0) | nr | nr | 48 (5.8) | nr | nr | nr |  |
| Jang 2016 ^21^ | 63 (100) | nr | 2 (3.2) | nr | nr | 2 (3.2) | nr | nr | 2 (3.2) | 0 (0) | nr | nr | 2 (3.2) | nr | nr | nr |  |
| Kaiser, 2022 ^22^ | 74 (100) | 87 (73-107) | 10 (13.5) | 3 (4.1) | 7 (9.5) | 0(0) | nr | nr | 10 (13.5) | 0 (0) | 10 (13.5) | 0 (0) | 0 (0) | low-grade MT-IPMN, ipmn associated pdac, 2 nr | 4 surgery  6 conservative | nr |  |
| Kaiser, 2023 ^23^ | 54 (91.5) | nr | 3 (5.1) | nr | 3 (5.1) | 0 (0) | (19-55) | 0 (0) | 3 (5.1) | 0 (0) | 2 (3.4) | 1 (1.7) | 0 (0) | MT  IPMN  CA | 1 surgery  2 conservative | nr |  |
| Kamata, 2018 ^24^ | 92 (100) | nr | 5 (5.4) | nr | nr | 5 (5.4) | 62 (12-69) | 1 (1.1) | 4 (4.3) | 0 (0) | 4 (12.5) | 1 (3.1) | 0 (0) | nr | nr | nr |  |
| Kang, 2014 ^25^ | 298 (100) | 44.4 (0.4-214) | 16 (5.2) | 3 (1.0) | 12 (4.0) | 1 (0.3) | 47.4 (0.4-213-5) | 6 (2.0) | 10 (3.4) | 0 (0) | nr | nr | 16 (5.2) | nr | nr | nr |  |
| S. Kim, 2008 ^26^ | 82 (100) | 29.2 | 8 (9.8) | nr | 8 (9.8) | 0 (0) | 25.5 (9-59) | 6 (7.3) | Nr | 2 (2.5) | nr | nr | 8 (9.8) | nr | nr | 5y 98.2% |  |
| H. Kim, 2022 ^27^ | 431 (100) | 56 (0.4-223) | 10 (2.3) | 4 (0.9) | 6 (1.4) | 0 (0) | nr | 2 (0.5) | 8 (1.8) | 0 (0) | nr | nr | 10 (2.3) | nr | 1 chemotp  2 palliative | 5 y LGD 89% 5 y HGD 84% |  |
| M. Kim, 2024 ^28^ | 133 (100) | nr | 21 (15.8) | 15 (11.3) | 6 (4.5) | 0 (0) | nr | nr | nr | 21 (15.8) | 21 (15.8) | 0 (0) | 0 (0) | nr | nr | nr |  |
| R. Kim, 2024 ^29^ | 481 (100) | 54 (0.2-127.2) | 72 (15.0) | nr | nr | 72 (15.0) | 36 | nr | 72 (15.0) | 0 (0) | nr | nr | 72 (15.0) | 4 PDAC | 16 surgery  56 conservative | nr |  |
| Kimura, 2017 ^30^ | 71 (100) | 55 (10-210) | 0 (0) | nr | nr | 0 (0) | nr | nr | nr | 0 (0) | nr | nr | 0 (0) | nr | nr | 5 y HGD 100% |  |
| Li, 2020 ^32^ | 125 (100) | nr | 12 (9.6) | nr | nr | 12 (9.6) | nr | nr | nr | 12 (9.6) | nr | nr | 12 (9.6) | nr | nr | 1y/3y/5y 100% |  |
| Lubezky, 2010 ^33^ | 38 (97.4) | 50 (12-102) | 3 (9.3) | 1 (3.1) | 2 (6.2) | 0 (0) | 24 | 1 (3.1) | 2 (6.2) | 0 (0) | 2 (6.2) | 1 (3.1) | 0 (0) | nr | 2 surgery  1 palliative | 5 y 92%, % |  |
| Majumder, 2019 ^35^ | 138 (100) | 48 | 6 (4.3) | nr | nr | 6 (4.3) | nr | nr | Nr | 6 (4.3) | 0 (0) | 6 (4.3) | 0 (0) | nr | nr | nr |  |
| Marchegiani, 2015 ^37^ | 299 (94.6) | nr | 28 (9.4) | nr | nr | 28 (9.4) | 52 (1-236) | nr | nr | 28 (9.4) | 22 (7.4) | 6 (2) | 0 (0) | nr | nr | 5 y 85% |  |
| Miller, 2011 ^39^ | 191 (100) | 66 | 32 (16.7) | 1 (0.5) | 31 (16.2) | 0 (0) | 36 | nr | nr | 32 (16.7) | 29 (15.2) | 3 (1.6) | 0 (0) | invasive cancer + lymph invasion,  1 microinvasive, 5 LGD, 2 HGD, 2 invasive, 4 no ipmn | 12 surgery | nr |  |
| Nagai, 2008 ^40^ | 42 (100) | 54 (1-267) | 0 (0) | nr | nr | 0 (0) | nr | nr | nr | 0 (0) | nr | nr | 0 (0) | nr | nr | 5 y 100%;  10 y 78.1%, |  |
| Nakagohri, 2002 ^41^ | 16 (100) | 78 (33-146) | 1 (6.3) | nr | 1 (6.3) | 0 (0) | nr | 0 (0) | 1 (6.3) | 0 (0) | 1 (6.3) | 0 (0) | 0 (0) | nr | 1 conservative | nr |  |
| Nakagohri, 2007 ^42^ | 14 (100) | 28 (1-153) | 1 (7.1) | 1 (7.1) | 0 (0) | 0 (0) | nr | 1 (7.1) | nr | 0 (0) | nr | 1 (7.1) | nr | nr | nr | 5 y 87%,  10 y 80% |  |
| Nakagohri, 2010 ^43^ | 13 (100) | 81 (24-164) | 0 (0) | 0 (0) | nr | nr | nr | nr | nr | 0 (0) | nr | nr | 0 (0) | nr | nr | 1 y 100%  3 y 92%  5 y 92%  10 y 83% |  |
| Niedergethmann, 2008 ^44^ | 29 (100) | 36 (1-124) | 4 (13.8) | nr | nr | 4 (13.8) | nr | nr | 1 (3.4) | 3 (10.3) | 1 (3.4) | nr | 3 (10.3) | CIS | 1 surgery | 10 y 90% |  |
| Park, 2011 ^45^ | 68 (100) | 36 | 1 (1.5) | nr | 1 (1.5) | 0 (0) | 8 | nr | 1 (1.5) | 0 (0) | nr | nr | 1 (1.5) | nr | nr | nr |  |
| Pea, 2017 ^47^ | 260 (100) | 52 (38-69) | 50 (19.2) | 13 (5) | 37 (14.2) | 0 (0) | nr | 7 (2.7) | 43 (16.5) | 0 (0) | nr | nr | 50 (19.2) | 2 lgd+ margin, 3hgd+  3md, 3 bd, 7 mixed | 13 surgery | nr |  |
| Pfluger, 2022 ^48^ | 124 (98.4) | 114 (12-204) | 35 (28.2) | 11 (8.9) | 15 (12.1) | 9 nr (7.3) | nr | 0 (0) | 35 (28.2) | 0 (0) | 19 (15.3) | 16 (12.9) | 0 (0) | 13 pdac,  6 ipmn | 12 surgery | nr |  |
| Ridtitid, 2016 ^49^ | 102 (87.2) | 53.9 | 8 (7.8) | nr | nr | 8 (7.8) | 21.5 | nr | 8 (7.8) | 0 (0) | 8 (7.8) | nr | nr | nr | nr | nr |  |
| Salvia 2004 ^50^ | 57 (100) | 40 | 1 (1.7) | nr | nr | 1 (1.7) | 60 | nr | 1 (1.7) | 0 (0) | nr | 1 (1.7) | 0 (0) | carcinoma in situ | 1 surgery | 5 y 100%,  10 y 100% |  |
| Sauvanet, 2014 ^51^ | 75 (97.4) | 50 (29-85) | 7 (9.3) | 3 (4) | 4 (5.3) | 0 (0) | nr | nr | Nr | 7 (9.3) | 7 (9.3) | 0 (0) | 0 (0) | 2 non invasive, 1 microinvasive | 3 surgery | nr |  |
| Schnelldorfer, 2008 ^52^ | 143 (98.6) | nr | 11 (7.7) | nr | nr | 11 (7.7) | nr | nr | 11 (7.7) | nr | 8 (5.6) | 3 (2.1) | 0 (0) | nr | 1 surgery  3 chemo  7 conservative | 5 y 94% |  |
| Stauffer, 2009 ^53^ | 21 (100) | 30 (2-59) | 0 (0) | nr | nr | 0 (0) | nr | nr | Nr | 0 (0) | nr | nr | 0 (0) | nr | nr | 6 months 100%  1y-/2y/3y- 95% |  |
| Sugimachi, 2021 ^54^ | 35 (100) | nr | 3 (8.6) | nr | nr | 3 (8.6) | nr | 1 (2.8) | 2 (5.7) | 0 (0) | nr | nr | 3 (8.6) | nr | nr | nr |  |
| Sugiyama, 2003 ^55^ | 19 (100) | mean 37.2 (10.8-81.6) | 1 (5.3) | nr | 1 (5.3) | 0 (0) | nr | nr | 1 (5.3) | 0 (0) | 1 (5.3) | nr | 0 (0) | nr | nr | nr |  |
| Takahashi, 2006 ^56^ | 20 (100) | 56.7 (7.3-159) | 2 (10) | nr | nr | 2 (10) | nr | 2 (10) | nr | 0 (0) | nr | 2 (10) | 0 (0) | Minimally invasive MD IPMN | 1 surgery  1 chemotherapy | nr |  |
| Tamura, 2014 ^57^ | 36 (100) | 53 (1-209) | 4 (11.1) | 2 (5.5) | 2 (5.5) | 0 (0) | 34.5 (19-97) | nr | 4 (11.1) | 0 (0) | 2 (5.5) | 2 (5.5) | 0 (0) | 2 HGD, 1 IPMN-derived PC, 1 PDAC | 4 surgery | 5 y 90% |  |
| Thomas, 2015 ^58^ | 17 (100) | 33.6 | 0 (0) | nr | nr | 0 (0) | nr | nr | nr | 0 (0) | nr | nr | 0 (0) | nr | nr | nr |  |
| Wada, 2005 ^59^ | 75 (100) | 31 | 1 (1.3) | nr | nr | 1 (1.3) | 36 | 0 (0) | 1 (1.3) | 0 (0) | 1 (1.3) | Nr | 0 (0) | nr | nr | 5 y 100% |  |
| Winner, 2013 ^60^ | 155 (100) | 32 | 15 (9.7) | nr | 15 (9.7) | 0 (0) | nr | 1 (0.6) | 14 (9.0) | 0 (0) | 13 (8.4) | 2 (1.3) | (0) | LGD, PDAC, IPMN-derived PC, | 3 surgery  8 conservative  3 nr | nr |  |
| Xourafas, 2015 ^62^ | 87 (100) | 39.4 | 14 (16.1) | 6 (6.9) | 8 (10) | 0 (0) | 28 | 0 (0) | 14 (16.1) | 0 (0) | nr | 14 (16.1) | nr | nr | 14 conservative | nr |  |
| Yamaguchi, 2016 ^63^ | 45 (100) | nr | 5 (11.1) | 5 (11.1) | nr | 0 (0) | nr | nr | nr | 5 (11.1) | nr | nr | 5 (11.1) | nr | nr | nr |  |
| Yogi, 2015 ^65^ | 118 (100) | 46.6 (6-216) | 7 (5.9) | nr | nr | 7 (5.9) | nr | nr | nr | 7 (5.9) | 3 (2.5) | 4 (3.4) | 0 (0) | nr | nr | nr |  |
| Yokoyama, 2007 ^66^ | 85 (100) | 48 (2-215 and 25-204) | 4 (4.7) | 4 (4.7) | 0 (0) | 0 (0) | nr | nr | nr | 4 (4.7) | 0 (0) | 4 (4.7) | 0 (0) | 3 non-invasive IPMN,  1 IPMN-derived PC | 4 surgery | 5 y 100%  10 y 100% |  |

Supplementary Table 14: Overview of diagnostic tools used to evaluate recurrent disease in non-invasive IPMN (n=number of patients).

| Study | Radiologic follow-up only, n | Radiologic follow-up and histologic confirmation, n |
| --- | --- | --- |
| Al Efishat, 2018 ^2^ |  | 319 |
| Aleotti, 2022 ^3^ |  | 118 |
| Amini, 2022 ^4^ |  | 449 |
| Antoñanzas J, 2018 ^5^ | 18 |  |
| Blackham, 2017 ^6^ | 100 |  |
| Blair, 2022 ^7^ | 127 |  |
| Chari, 2002 ^8^ |  | 73 |
| Chunhui, 2014 ^9^ | 24 |  |
| Crippa, 2010 ^10^ | 263 |  |
| Cuillerier, 2000 ^11^ |  | 25 |
| Dhar, 2018 ^12^ |  | 330 |
| Faitot, 2015 ^13^ |  | 38 |
| Frankel, 2013 ^14^ |  | 192 |
| Fuji, 2011 ^15^ | 84 |  |
| Fuji, 2022 ^16^ |  | 61 |
| Hirono, 2016 ^18^ |  | 172 |
| Hirono, 2020 ^19^ |  | 827 |
| Jang, 2016 ^21^ | 63 |  |
| Kaiser, 2022 ^22^ | 74 |  |
| Kaiser, 2023 ^23^ | 54 |  |
| Kamata, 2018 ^24^ | 92 |  |
| Kang, 2014 ^25^ |  | 298 |
| S. Kim, 2008 ^26^ | 82 |  |
| H. Kim, 2022 ^27^ |  | 431 |
| M. Kim, 2024 ^28^ | 133 |  |
| R. Kim, 2024 ^29^ | 481 |  |
| Kimura, 2017 ^30^ | 71 |  |
| Li, 2020 ^32^ |  | 125 |
| Lubezky, 2010 ^33^ | 38 |  |
| Majumder, 2019 ^35^ | 138 |  |
| Marchegiani, 2015 (patterns) ^37^ | 299 |  |
| Miller, 2011 ^39^ |  | 191 |
| Nagai, 2008 ^40^ | 42 |  |
| Nakagohri, 2002 ^41^ | 16 |  |
| Nakagohri, 2007 ^42^ | 14 |  |
| Nakagohri, 2010 ^43^ | 13 |  |
| Niedergethmann, 2008 ^44^ |  | 29 |
| Park, 2011 ^45^ |  | 68 |
| Pea, 2017 ^47^ |  | 260 |
| Pfluger, 2022 ^48^ | 124 |  |
| Ridtitid, 2016 ^49^ |  | 102 |
| Salvia, 2004 ^50^ | 57 |  |
| Sauvanet, 2014 ^51^ | 75 |  |
| Schnelldorfer, 2008 ^52^ | 143 |  |
| Stauffer, 2009 ^53^ | 21 |  |
| Sugimachi, 2021 ^54^ | 35 |  |
| Sugiyama, 2003 ^55^ | 19 |  |
| Takahashi, 2006 ^56^ | 20 |  |
| Tamura, 2014 ^57^ |  | 36 |
| Thomas, 2015 ^58^ | 17 |  |
| Wada, 2005 ^59^ |  | 75 |
| Winner, 2013 ^60^ |  | 155 |
| Xourafas, 2015 ^62^ | 87 |  |
| Yamaguchi, 2016 ^63^ | 45 |  |
| Yogi, 2015 ^65^ |  | 118 |
| Yokoyama, 2007 ^66^ | 85 |  |
| **Total** | **2954** | **4492** |

**Supplementary Figures**
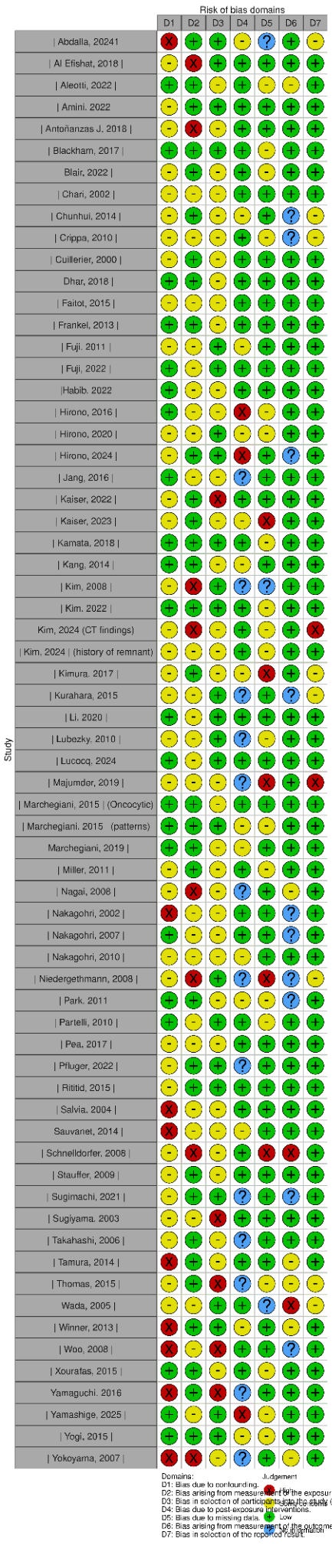


Supplementary Figure 1: Risk of Bias assessment of all included studies


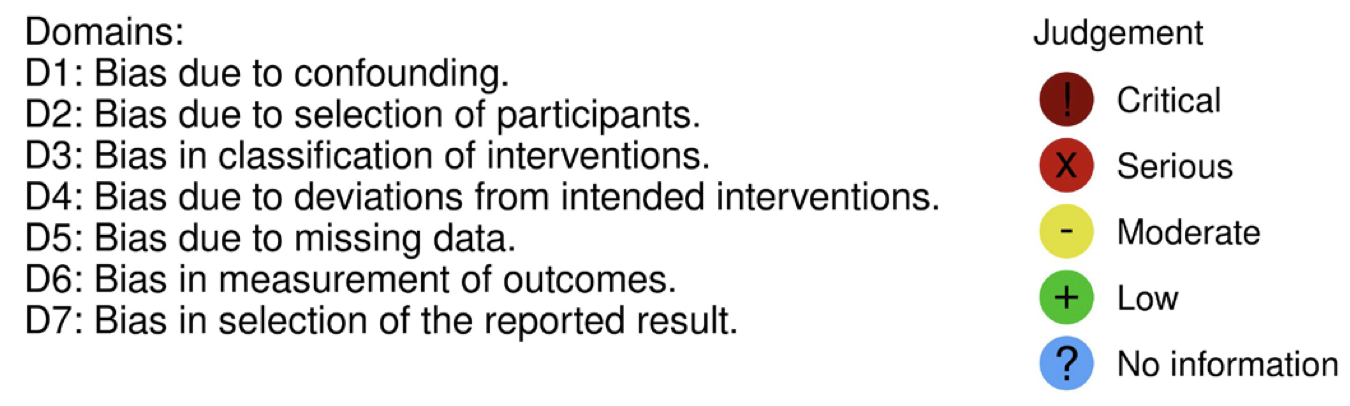


Supplementary Figure 2: Forrest plots of a 1-, b 3-, c 5- and d 10y recurrence-free-survival following surgical resection of IPMN-derived PC.


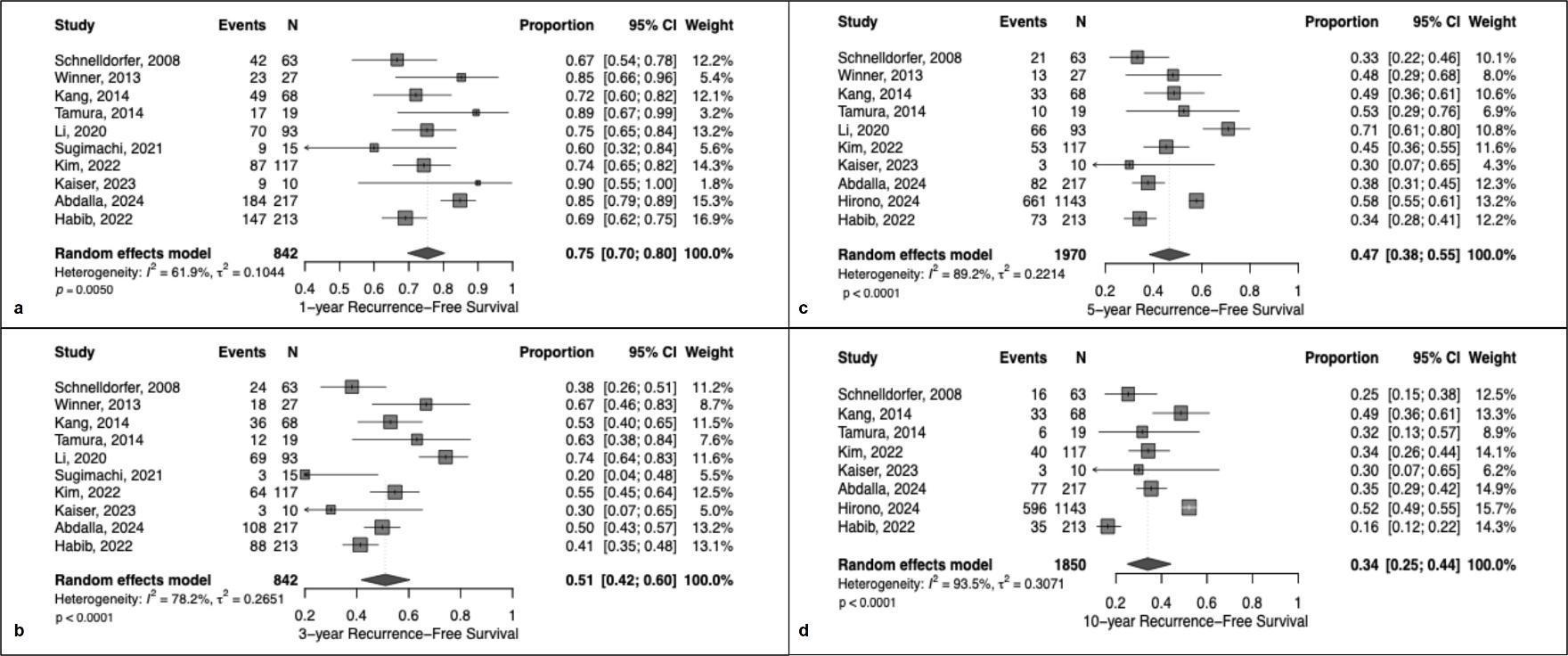


Supplementary Figure 3: Forrest plots displaying results of the sensitivity analysis performed as a “Leave-One-Out” analysis for **a** pooled 5-year RFS and **b** pooled 10-year RFS.


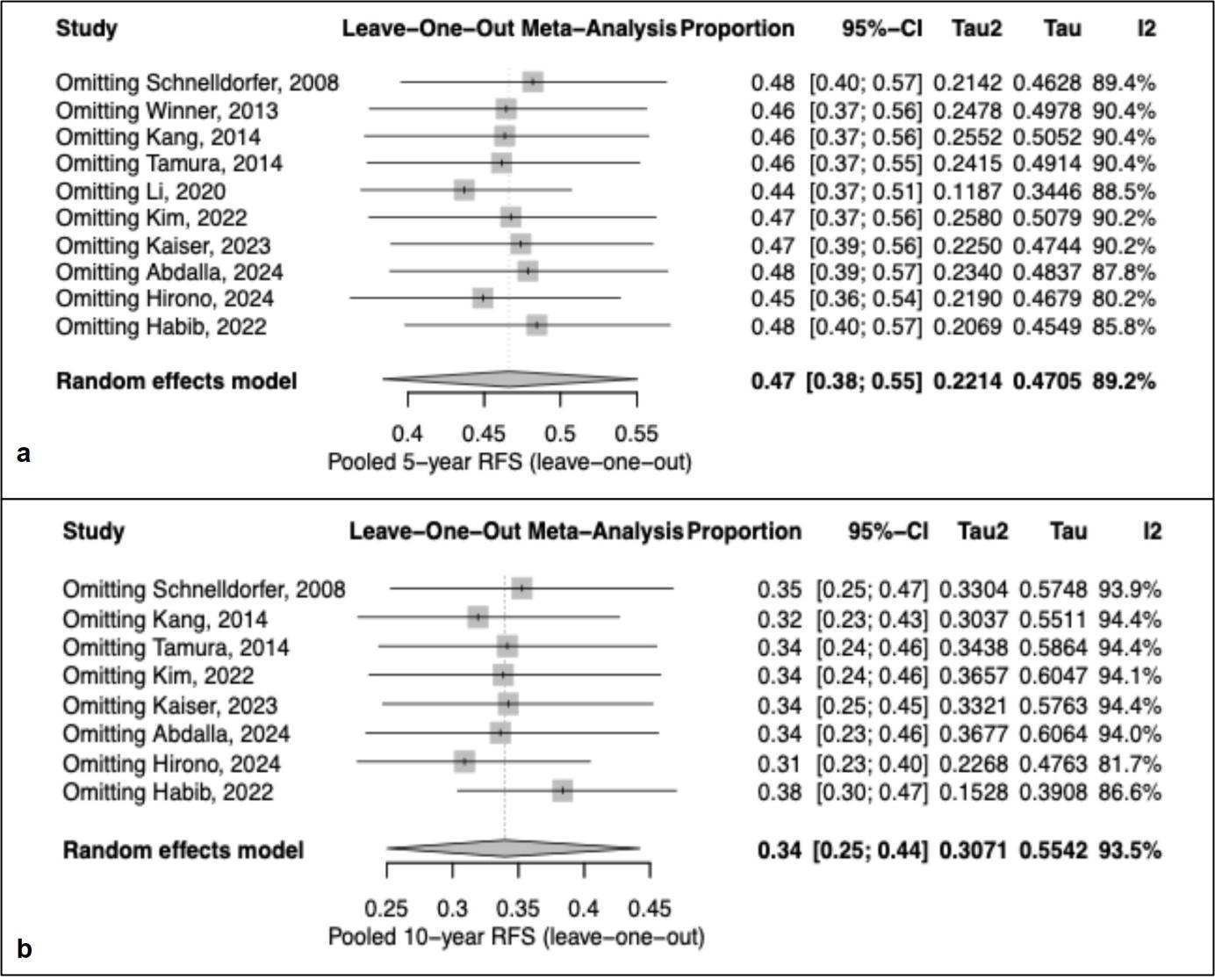


Supplementary Figure 4: Forrest plots visualizing results of the subgroup analysis performed by stratification of publication year (< 2020 vs. ≥ 2020) for **a** 5-year RFS and **b** 10-year RFS.


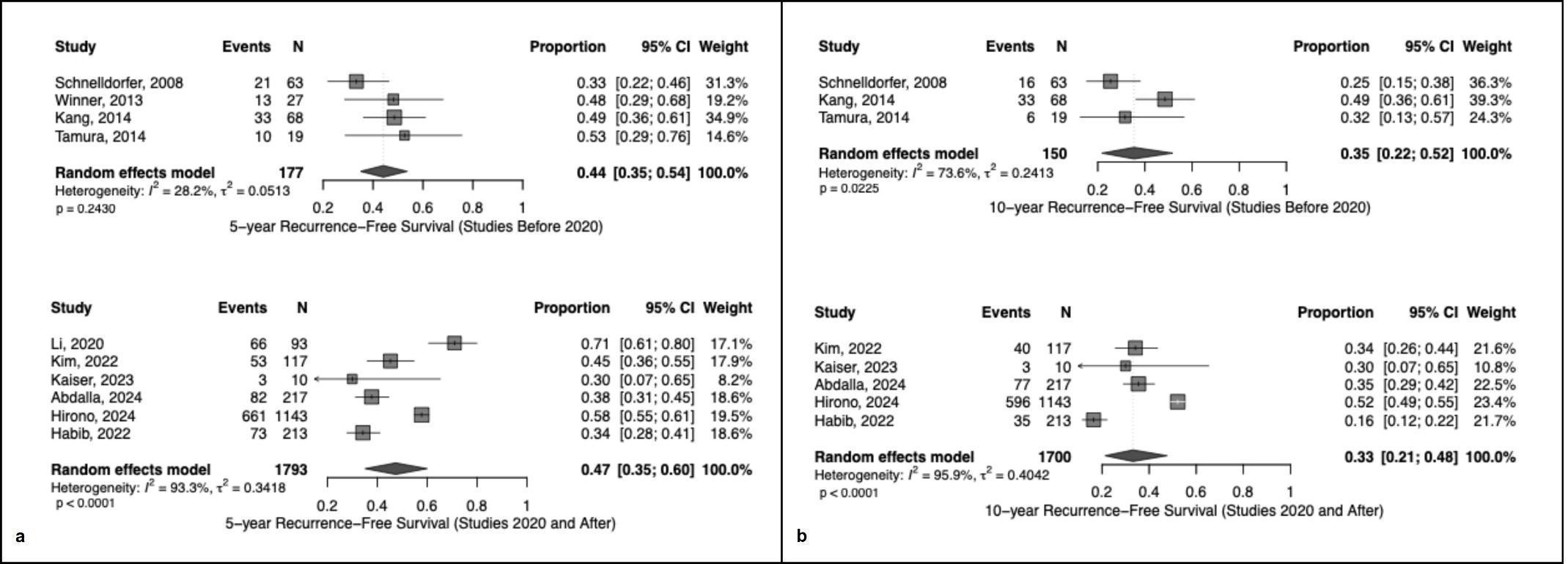


Supplementary Figure 5: Forrest plots visualizing results of the single center subgroup analysis after exclusion of multicenter data (Li,2020^32^ and Hirono, 2024^20^) for **a** 5-year RFS and **b** 10-year RFS.


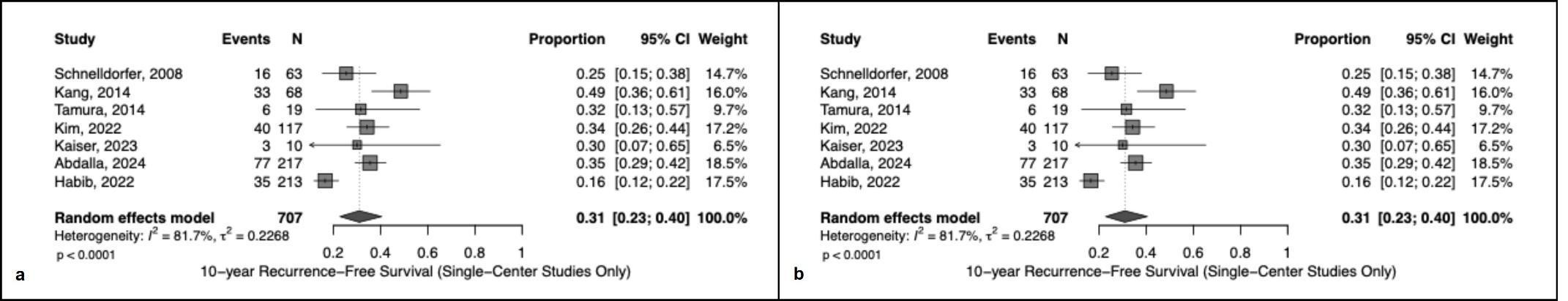


Supplementary Figure 6: Forrest plots visualizing results of the risk of bias stratified subgroup analysis after exclusion of all data derived from studies rated with a high risk of bias^1,20,23,52,57,60^ for **a** 1-year RFS, **b** 3-year RFS, **c** 5-year RFS and **d** 10-year RFS.

**
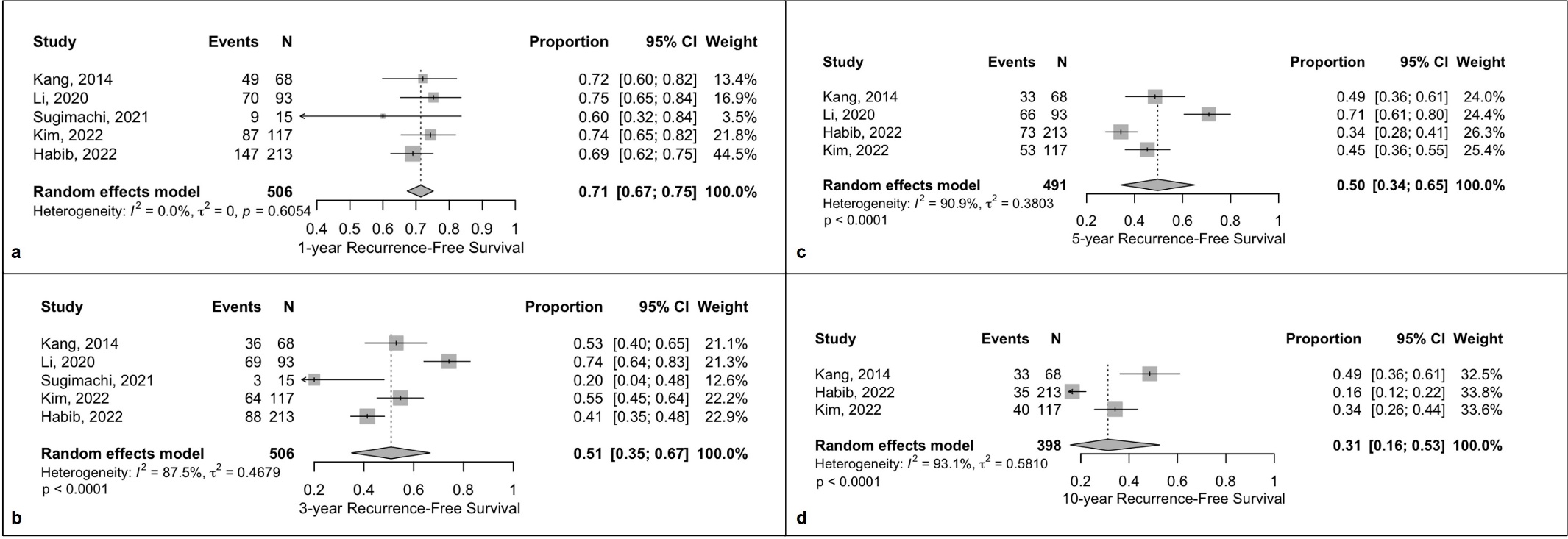
**

**Supplementary References**

1. Abdalla TSA, Duhn J, Klinkhammer-Schalke M, et al. Oncological Outcomes and Patterns of Recurrence after the Surgical Resection of an Invasive Intraductal Papillary Mucinous Neoplasm versus Primary Pancreatic Ductal Adenocarcinoma: An Analysis from the German Cancer Registry Group of the Society of German Tumor Centers. *Cancers (Basel)*. May 26 2024;16(11)doi:10.3390/cancers16112016

2. Al Efishat M, Attiyeh MA, Eaton AA, et al. Progression Patterns in the Remnant Pancreas after Resection of Non-Invasive or Micro-Invasive Intraductal Papillary Mucinous Neoplasms (IPMN). *Ann Surg Oncol*. Jun 2018;25(6):1752-1759. doi:10.1245/s10434-018-6445-2

3. Aleotti F, Crippa S, Belfiori G, et al. Pancreatic resections for benign intraductal papillary mucinous neoplasms: Collateral damages from friendly fire. *Surgery*. Oct 2022;172(4):1202-1209. doi:10.1016/j.surg.2022.04.036

4. Amini N, Habib JR, Blair A, et al. Invasive and Noninvasive Progression After Resection of Noninvasive Intraductal Papillary Mucinous Neoplasms. *Ann Surg*. Aug 1 2022;276(2):370-377. doi:10.1097/sla.0000000000004488

5. Antoñanzas J, Cienfuegos JA, Hurtado-Pardo L, et al. Intraductal papillary mucinous neoplasm (IPMN) of the pancreas: clinicopathological features and long-term outcomes following a pancreatectomy. *Rev Esp Enferm Dig*. Dec 2018;110(12):768-774. doi:10.17235/reed.2018.5646/2018

6. Blackham AU, Doepker MP, Centeno BA, et al. Patterns of recurrence and long-term outcomes in patients who underwent pancreatectomy for intraductal papillary mucinous neoplasms with high grade dysplasia: implications for surveillance and future management guidelines. *HPB*. 2017;19(7):603-610. doi:10.1016/j.hpb.2017.03.007

7. Blair AB, Beckman RM, Habib JR, et al. Should non-invasive diffuse main-duct intraductal papillary mucinous neoplasms be treated with total pancreatectomy? *HPB (Oxford)*. May 2022;24(5):645-653. doi:10.1016/j.hpb.2021.09.013

8. Chari ST, Yadav D, Smyrk TC, et al. Study of recurrence after surgical resection of intraductal papillary mucinous neoplasm of the pancreas. *Gastroenterology*. Nov 2002;123(5):1500-7. doi:10.1053/gast.2002.36552

9. Yuan C, Xiu D, Tao M, et al. Data analysis of 36 cases with intraductal papillary mucinous neoplasm of the pancreas for their clinicopathological features, diagnosis, and treatment. *Chinese Medical Journal*. 2014;127(23):4087-4091. doi:doi:10.3760/cma.j.issn.0366-6999.20142024

10. Crippa S, Fernández-Del Castillo C, Salvia R, et al. Mucin-producing neoplasms of the pancreas: an analysis of distinguishing clinical and epidemiologic characteristics. *Clin Gastroenterol Hepatol*. Feb 2010;8(2):213-9. doi:10.1016/j.cgh.2009.10.001

11. Cuillerier E, Cellier C, Palazzo L, et al. Outcome after surgical resection of intraductal papillary and mucinous tumors of the pancreas. *Am J Gastroenterol*. Feb 2000;95(2):441-5. doi:10.1111/j.1572-0241.2000.01764.x

12. Dhar VK, Merchant NB, Patel SH, et al. Does Surgical Margin Impact Recurrence in Noninvasive Intraductal Papillary Mucinous Neoplasms?: A Multi-institutional Study. *Ann Surg*. Sep 2018;268(3):469-478. doi:10.1097/sla.0000000000002923

13. Faitot F, Gaujoux S, Barbier L, et al. Reappraisal of pancreatic enucleations: A single-center experience of 126 procedures. *Surgery*. 2015;158(1):201-210. doi:10.1016/j.surg.2015.03.023

14. Frankel TL, LaFemina J, Bamboat ZM, et al. Dysplasia at the surgical margin is associated with recurrence after resection of non-invasive intraductal papillary mucinous neoplasms. *HPB (Oxford)*. Oct 2013;15(10):814-21. doi:10.1111/hpb.12137

15. Fujii T, Kanda M, Kodera Y, et al. Comparison of pancreatic head resection with segmental duodenectomy and pylorus-preserving pancreatoduodenectomy for benign and low-grade malignant neoplasms of the pancreatic head. *Pancreas*. Nov 2011;40(8):1258-63. doi:10.1097/MPA.0b013e318220b1c0

16. Fuji T, Umeda Y, Takagi K, et al. Optimal surveillance of intraductal papillary mucinous neoplasms of the pancreas focusing on remnant pancreas recurrence after surgical resection. *BMC Cancer*. 2022/05/29 2022;22(1):588. doi:10.1186/s12885-022-09650-w

17. Habib JR, Kinny-Köster B, Amini N, et al. Predictors, Patterns, and Timing of Recurrence Provide Insight into the Disease Biology of Invasive Carcinomas Arising in Association with Intraductal Papillary Mucinous Neoplasms. *J Gastrointest Surg*. Nov 2022;26(11):2311-2320. doi:10.1007/s11605-022-05428-4

18. Hirono S, Kawai M, Okada K-i, et al. Long-term surveillance is necessary after operative resection for intraductal papillary mucinous neoplasm of the pancreas. *Surgery*. 2016;160(2):306-317. doi:10.1016/j.surg.2016.04.007

19. Hirono S, Shimizu Y, Ohtsuka T, et al. Recurrence patterns after surgical resection of intraductal papillary mucinous neoplasm (IPMN) of the pancreas; a multicenter, retrospective study of 1074 IPMN patients by the Japan Pancreas Society. *Journal of Gastroenterology*. 2020/01/01 2020;55(1):86-99. doi:10.1007/s00535-019-01617-2

20. Hirono S, Higuchi R, Honda G, et al. Is multidisciplinary treatment effective for invasive intraductal papillary mucinous carcinoma? *Ann Gastroenterol Surg*. Sep 2024;8(5):845-859. doi:10.1002/ags3.12790

21. Jang DK, Ryu JK, Chung KH, et al. Risk Factors for Progression or Malignancy in Main-Duct and Mixed-Type Intraductal Papillary Mucinous Neoplasm of the Pancreas. *Pancreas*. Aug 2016;45(7):1027-31. doi:10.1097/mpa.0000000000000592

22. Kaiser J, Alhalabi KT, Hinz U, et al. Enucleation for low-grade branch duct intraductal papillary mucinous neoplasms: Long-term follow-up. *Surgery*. 2022;172(3):968-974. doi:10.1016/j.surg.2022.04.035

23. Kaiser J, Hackert T, Hinz U, et al. Surgery for intraductal papillary mucinous neoplasms in young patients: High-risk population. *Surgery*. 2023;174(2):330-336. doi:10.1016/j.surg.2023.04.045

24. Kamata K, Takenaka M, Minaga K, et al. Value of additional endoscopic ultrasonography for surveillance after surgical removal of intraductal papillary mucinous neoplasms. *Digestive Endoscopy*. 2018;30(5):659-666. doi:<https://doi.org/10.1111/den.13176>

25. Kang MJ, Jang JY, Lee KB, Chang YR, Kwon W, Kim SW. Long-term prospective cohort study of patients undergoing pancreatectomy for intraductal papillary mucinous neoplasm of the pancreas: implications for postoperative surveillance. *Ann Surg*. Aug 2014;260(2):356-63. doi:10.1097/sla.0000000000000470

26. Kim SC, Park KT, Lee YJ, et al. Intraductal papillary mucinous neoplasm of the pancreas: clinical characteristics and treatment outcomes of 118 consecutive patients from a single center. *J Hepatobiliary Pancreat Surg*. 2008;15(2):183-8. doi:10.1007/s00534-007-1231-8

27. Kim HS, Han Y, Kang JS, et al. Fate of Patients With Intraductal Papillary Mucinous Neoplasms of Pancreas After Resection According to the Pathology and Margin Status: Continuously Increasing Risk of Recurrence Even After Curative Resection Suggesting Necessity of Lifetime Surveillance. *Ann Surg*. Oct 1 2022;276(4):e231-e238. doi:10.1097/sla.0000000000004478

28. Kim MC, Kim JH, Jeon SK, Kang HJ. CT findings and clinical effects of high grade pancreatic intraepithelial neoplasia in patients with intraductal papillary mucinous neoplasms. *PLoS One*. 2024;19(4):e0298278. doi:10.1371/journal.pone.0298278

29. Kim RC, Perri G, Rocha Castellanos DM, et al. Natural History of the Remnant Pancreatic Duct after Pancreatoduodenectomy for Non-Invasive Intraductal Papillary Mucinous Neoplasm: Results from an International Consortium. *Ann Surg*. Sep 3 2024;doi:10.1097/sla.0000000000006519

30. Kimura K, Amano R, Ymazoe S, et al. The Clinical Indications for Limited Surgery of Intraductal Papillary Mucinous Neoplasms of the Pancreas. *World J Surg*. May 2017;41(5):1358-1365. doi:10.1007/s00268-016-3824-3

31. Kurahara H, Maemura K, Mataki Y, et al. Predictors of early stages of histological progression of branch duct IPMN. *Langenbecks Arch Surg*. Jan 2015;400(1):49-56. doi:10.1007/s00423-014-1259-6

32. Li Y, Zhu Z, Peng L, Jin Z, Sun L, Song B. The pathological features and prognoses of intraductal papillary mucinous neoplasm and mucinous cystic neoplasm after surgical resection: a single institution series. *World Journal of Surgical Oncology*. 2020/11/04 2020;18(1):287. doi:10.1186/s12957-020-02063-8

33. Lubezky N, Ben-Haim M, Nakache R, et al. Clinical presentation can predict disease course in patients with intraductal papillary mucinous neoplasm of the pancreas. *World J Surg*. Jan 2010;34(1):126-32. doi:10.1007/s00268-009-0269-y

34. Lucocq J, Haugk B, Joseph N, et al. Invasive intraductal oncocytic papillary neoplasms (IOPN) and adenocarcimoma arising from intraductal papillary mucinous neoplasms (A-IPMN) of the pancreas: comparative analysis of clinicopathological features, patterns of recurrence and survival: a multicentre study. *HPB (Oxford)*. Nov 2024;26(11):1421-1428. doi:10.1016/j.hpb.2024.07.410

35. Majumder S, Philip NA, Singh Nagpal SJ, et al. High-Grade Dysplasia in Resected Main-Duct Intraductal Papillary Mucinous Neoplasm (MD-IPMN) is Associated with an Increased Risk of Subsequent Pancreatic Cancer. *Official journal of the American College of Gastroenterology | ACG*. 2019;114(3)

36. Marchegiani G, Mino-Kenudson M, Ferrone CR, Warshaw AL, Lillemoe KD, Fernández-del Castillo C. Oncocytic-type intraductal papillary mucinous neoplasms: a unique malignant pancreatic tumor with good long-term prognosis. *J Am Coll Surg*. May 2015;220(5):839-44. doi:10.1016/j.jamcollsurg.2015.01.051

37. Marchegiani G, Mino-Kenudson M, Ferrone CR, et al. Patterns of Recurrence After Resection of IPMN: Who, When, and How? *Ann Surg*. Dec 2015;262(6):1108-14. doi:10.1097/sla.0000000000001008

38. Marchegiani G, Andrianello S, Dal Borgo C, et al. Adjuvant chemotherapy is associated with improved postoperative survival in specific subtypes of invasive intraductal papillary mucinous neoplasms (IPMN) of the pancreas: it is time for randomized controlled data. *HPB*. 2019;21(5):596-603. doi:10.1016/j.hpb.2018.09.013

39. Miller JR, Meyer JE, Waters JA, et al. Outcome of the pancreatic remnant following segmental pancreatectomy for non-invasive intraductal papillary mucinous neoplasm. *HPB (Oxford)*. Nov 2011;13(11):759-66. doi:10.1111/j.1477-2574.2011.00354.x

40. Nagai K, Doi R, Kida A, et al. Intraductal papillary mucinous neoplasms of the pancreas: clinicopathologic characteristics and long-term follow-up after resection. *World J Surg*. Feb 2008;32(2):271-8; discussion 279-80. doi:10.1007/s00268-007-9281-2

41. Nakagohri T, Asano T, Kenmochi T, Urashima T, Ochiai T. Long-term surgical outcome of noninvasive and minimally invasive intraductal papillary mucinous adenocarcinoma of the pancreas. *World J Surg*. Sep 2002;26(9):1166-9. doi:10.1007/s00268-002-6254-3

42. Nakagohri T, Kinoshita T, Konishi M, Takahashi S, Gotohda N. Surgical outcome of intraductal papillary mucinous neoplasms of the pancreas. *Ann Surg Oncol*. Nov 2007;14(11):3174-80. doi:10.1245/s10434-007-9546-x

43. Nakagohri T, Kinoshita T, Konishi M, et al. Inferior head resection of the pancreas for intraductal papillary mucinous neoplasms. *J Hepatobiliary Pancreat Sci*. Nov 2010;17(6):798-802. doi:10.1007/s00534-009-0173-8

44. Niedergethmann M, Grützmann R, Hildenbrand R, et al. Outcome of invasive and noninvasive intraductal papillary-mucinous neoplasms of the pancreas (IPMN): a 10-year experience. *World J Surg*. Oct 2008;32(10):2253-60. doi:10.1007/s00268-008-9692-8

45. Park J, Lee KT, Jang TH, et al. Risk factors associated with the postoperative recurrence of intraductal papillary mucinous neoplasms of the pancreas. *Pancreas*. Jan 2011;40(1):46-51. doi:10.1097/MPA.0b013e3181f66b74

46. Partelli S, Fernandez-Del Castillo C, Bassi C, et al. Invasive intraductal papillary mucinous carcinomas of the pancreas: predictors of survival and the role of lymph node ratio. *Ann Surg*. Mar 2010;251(3):477-82. doi:10.1097/SLA.0b013e3181cf9155

47. Pea A, Yu J, Rezaee N, et al. Targeted DNA Sequencing Reveals Patterns of Local Progression in the Pancreatic Remnant Following Resection of Intraductal Papillary Mucinous Neoplasm (IPMN) of the Pancreas. *Ann Surg*. Jul 2017;266(1):133-141. doi:10.1097/sla.0000000000001817

48. Pflüger MJ, Griffin JF, Hackeng WM, et al. The Impact of Clinical and Pathological Features on Intraductal Papillary Mucinous Neoplasm Recurrence After Surgical Resection: Long-Term Follow-Up Analysis. *Ann Surg*. Jun 1 2022;275(6):1165-1174. doi:10.1097/sla.0000000000004427

49. Ridtitid W, DeWitt JM, Schmidt CM, et al. Management of branch-duct intraductal papillary mucinous neoplasms: a large single-center study to assess predictors of malignancy and long-term outcomes. *Gastrointest Endosc*. Sep 2016;84(3):436-45. doi:10.1016/j.gie.2016.02.008

50. Salvia R, Fernández-del Castillo C, Bassi C, et al. Main-duct intraductal papillary mucinous neoplasms of the pancreas: clinical predictors of malignancy and long-term survival following resection. *Ann Surg*. May 2004;239(5):678-85; discussion 685-7. doi:10.1097/01.sla.0000124386.54496.15

51. Sauvanet A, Gaujoux S, Blanc B, et al. Parenchyma-Sparing Pancreatectomy for Presumed Noninvasive Intraductal Papillary Mucinous Neoplasms of the Pancreas. *Annals of Surgery*. 2014;260(2):364-371. doi:10.1097/sla.0000000000000601

52. Schnelldorfer T, Sarr MG, Nagorney DM, et al. Experience with 208 resections for intraductal papillary mucinous neoplasm of the pancreas. *Arch Surg*. Jul 2008;143(7):639-46; discussion 646. doi:10.1001/archsurg.143.7.639

53. Stauffer JA, Nguyen JH, Heckman MG, et al. Patient outcomes after total pancreatectomy: a single centre contemporary experience. *HPB (Oxford)*. Sep 2009;11(6):483-92. doi:10.1111/j.1477-2574.2009.00077.x

54. Sugimachi K, Mano Y, Matsumoto Y, et al. Neutrophil-to-lymphocyte Ratio as a Predictor of Malignancy of Intraductal Papillary Mucinous Neoplasms. *Anticancer Res*. Mar 2021;41(3):1663-1669. doi:10.21873/anticanres.14929

55. Sugiyama M, Abe N, Tokuhara M, et al. Magnetic resonance cholangiopancreatography for postoperative follow-up of intraductal papillary-mucinous tumors of the pancreas. *Am J Surg*. Mar 2003;185(3):251-5. doi:10.1016/s0002-9610(02)01371-5

56. Takahashi H, Nakamori S, Nakahira S, et al. Surgical outcomes of noninvasive and minimally invasive intraductal papillary-mucinous neoplasms of the pancreas. *Ann Surg Oncol*. Jul 2006;13(7):955-60. doi:10.1245/aso.2006.05.043

57. Tamura K, Ohtsuka T, Ideno N, et al. Treatment strategy for main duct intraductal papillary mucinous neoplasms of the pancreas based on the assessment of recurrence in the remnant pancreas after resection: a retrospective review. *Ann Surg*. Feb 2014;259(2):360-8. doi:10.1097/SLA.0b013e3182a690ff

58. Thomas E, Matsuoka L, Alexopoulos S, Selby R, Parekh D. Laparoscopic Hand-Assisted Parenchymal-Sparing Resections for Presumed Side-Branch Intraductal Papillary Mucinous Neoplasms. *J Laparoendosc Adv Surg Tech A*. Aug 2015;25(8):668-71. doi:10.1089/lap.2014.0669

59. Wada K, Kozarek RA, Traverso LW. Outcomes following resection of invasive and noninvasive intraductal papillary mucinous neoplasms of the pancreas. *Am J Surg*. May 2005;189(5):632-6; discussion 637. doi:10.1016/j.amjsurg.2005.01.020

60. Winner M, Epelboym I, Remotti H, et al. Predictors of recurrence in intraductal papillary mucinous neoplasm: experience with 183 pancreatic resections. *J Gastrointest Surg*. Sep 2013;17(9):1618-26. doi:10.1007/s11605-013-2242-1

61. Woo SM, Ryu JK, Lee SH, et al. Survival and prognosis of invasive intraductal papillary mucinous neoplasms of the pancreas: comparison with pancreatic ductal adenocarcinoma. *Pancreas*. Jan 2008;36(1):50-5. doi:10.1097/MPA.0b013e31812575df

62. Xourafas D, Tavakkoli A, Clancy TE, Ashley SW. Noninvasive intraductal papillary mucinous neoplasms and mucinous cystic neoplasms: Recurrence rates and&#xa0;postoperative imaging follow-up. *Surgery*. 2015;157(3):473-483. doi:10.1016/j.surg.2014.09.028

63. Yamaguchi J, Kaneoka Y, Maeda A, Takayama Y, Onoe S, Isogai M. Positive surgical margins in surgically treated unifocal and multifocal IPMN. *Int J Surg*. Apr 2016;28:51-5. doi:10.1016/j.ijsu.2016.02.065

64. Yamashige D, Hijioka S, Shimizu Y, et al. Clinical impact of epithelial types on postoperative outcomes for intraductal papillary mucinous neoplasms: a multicenter retrospective study. *Journal of Gastroenterology*. 2025/05/01 2025;60(5):658-670. doi:10.1007/s00535-025-02225-z

65. Yogi T, Hijioka S, Imaoka H, et al. Risk factors for postoperative recurrence of intraductal papillary mucinous neoplasms of the pancreas based on a long-term follow-up study: proposals for follow-up strategies. *J Hepatobiliary Pancreat Sci*. Oct 2015;22(10):757-65. doi:10.1002/jhbp.280

66. Yokoyama Y, Nagino M, Oda K, et al. Clinicopathologic features of re-resected cases of intraductal papillary mucinous neoplasms (IPMNs). *Surgery*. Aug 2007;142(2):136-42. doi:10.1016/j.surg.2007.03.006
